# Comprehensive review of the evidence regarding the effectiveness of community–based primary health care in improving maternal, neonatal and child health: 4. child health findings

**DOI:** 10.7189/jogh.07.010904

**Published:** 2017-06

**Authors:** Paul A Freeman, Meike Schleiff, Emma Sacks, Bahie M Rassekh, Sundeep Gupta, Henry B Perry

**Affiliations:** 1 Independent consultant, Seattle, Washington, USA; 2 University of Washington School of Public Health, Seattle, Washington, USA; 3 Department of International Health, Johns Hopkins Bloomberg School of Public Health, Baltimore, Maryland, USA; 4 The World Bank, Washington, District of Columbia, USA; 5 Medical Epidemiologist, Lusaka, Zambia

## Abstract

**Background:**

This paper assesses the effectiveness of community–based primary health care (CBPHC) in improving child health beyond the neonatal period. Although there has been an accelerated decline in global under–5 mortality since 2000, mortality rates remain high in much of sub–Saharan Africa and in some south Asian countries where under–5 mortality is also decreasing more slowly. Essential interventions for child health at the community level have been identified. Our review aims to contribute further to this knowledge by examining how strong the evidence is and exploring in greater detail what specific interventions and implementation strategies appear to be effective.

**Methods:**

We reviewed relevant documents from 1950 onwards using a detailed protocol. Peer reviewed documents, reports and books assessing the impact of one or more CBPHC interventions on child health (defined as changes in population coverage of one or more key child survival interventions, nutritional status, serious morbidity or mortality) among children in a geographically defined population was examined for inclusion. Two separate reviews took place of each document followed by an independent consolidated summative review. Data from the latter review were transferred to electronic database for analysis.

**Results:**

The findings provide strong evidence that the major causes of child mortality in resource–constrained settings can be addressed at the community level largely by engaging communities and supporting community–level workers. For all major categories of interventions (nutritional interventions; control of pneumonia, diarrheal disease and malaria; HIV prevention and treatment; immunizations; integrated management of childhood diseases; and comprehensive primary health care) we have presented randomized controlled trials that have consistently produced statistically significant and operationally important effects.

**Conclusions:**

This review shows that there is strong evidence of effectiveness for CBPHC implementation of an extensive range of interventions to improve child health and that four major strategies for delivering these interventions are effective.

This paper concentrates on the effectiveness of community–based primary health care (CBPHC) in improving the health of children beyond the neonatal period. In 2015, the global mortality rate for children younger than 5 years of age (referred to hereafter as under–5 mortality) was 42.5 per 1000 live births, a decline from 90.4 per 1000 live births in 1990 [1]. Although there has been an accelerated decline in global under–5 mortality since 2000, mortality rates remain high in much of sub–Saharan Africa and in some south Asian countries where under–5 mortality is also decreasing more slowly [1]. Following the neonatal period (when 45% of under–5 deaths occur currently), the major causes of mortality in children are pneumonia (26% of deaths in this age group), diarrhea (18%), and malaria (12%) [2]. Undernutrition is a cause of 45% of all under–5 deaths [3].

Essential interventions for child health at the community level have been identified as: promotion of breastfeeding and complementary feeding, supplementation with vitamin A and zinc, immunizations, co–trimoxazole for HIV–positive children, education on the safe disposal of feces and hand washing, distribution and promotion of insecticide–treated bed nets (ITNs) or indoor residual spraying (IRS) or both; detection and treatment or referral of children with severe acute undernutrition; and detection and treatment of pneumonia, malaria and diarrhea without danger signs and referral if danger signs appear [4]. It has been estimated that scaling up these interventions with an essential package of community–based interventions would avert 1.5 million deaths of children 1–59 months each year [1].

Our review aims to contribute further to this knowledge by examining how strong is the evidence for community–based primary health care (CBPHC) and exploring in greater detail what specific activities appear to be effective. Our concern is not just to strengthen the evidence about which interventions work at the community level but who does them and how, what conditions facilitate effectiveness, and what kinds of community–based approaches appear to be most effective. What characteristics do effective CBPHC activities share, and how strong is the evidence that partnerships between communities and health systems are required in order to improve child and maternal health?

The purpose of this paper is to summarize the evidence regarding the effectiveness of CBPHC for improving child health beyond the neonatal period.

## METHODS

Our review aims to provide a comprehensive review of documents from 1950 onwards assessing the effectiveness of projects, programs and research studies (hereafter referred to as projects) using a detailed protocol. We examined peer–reviewed articles, reports and books assessing the impact of one or more CBPHC interventions on child health (coverage of a key evidence–based child survival indicator, nutritional status, serious morbidity, or mortality), among children in a geographically defined population. Two independent reviews were carried out and followed by an independent consolidated summative review. Data from the latter review were transferred to an electronic database for analysis. Data analysis took place using EPI INFO version 3.5.4 (Epi Info, US Centers for Disease Control and Prevention, Atlanta, Georgia, USA).

Only those assessments which had clear documentation of the intervention(s) and their impact on child health where included. Outcome measures included were changes in the population coverage of one or more evidence–based interventions; change in nutritional status (as measured by anthropometry, anemia, or assessment of micro–nutrient deficiency); change in the incidence or in the outcome of serious, life–threatening morbidity (such as pneumonia, diarrhea, malaria, and low–birth weight); and change in mortality (infant, 1–4 year, and under–5 mortality). Further details regarding the methodology are reported elsewhere in this series [5].

## RESULTS

### General findings

**Figure 1:**
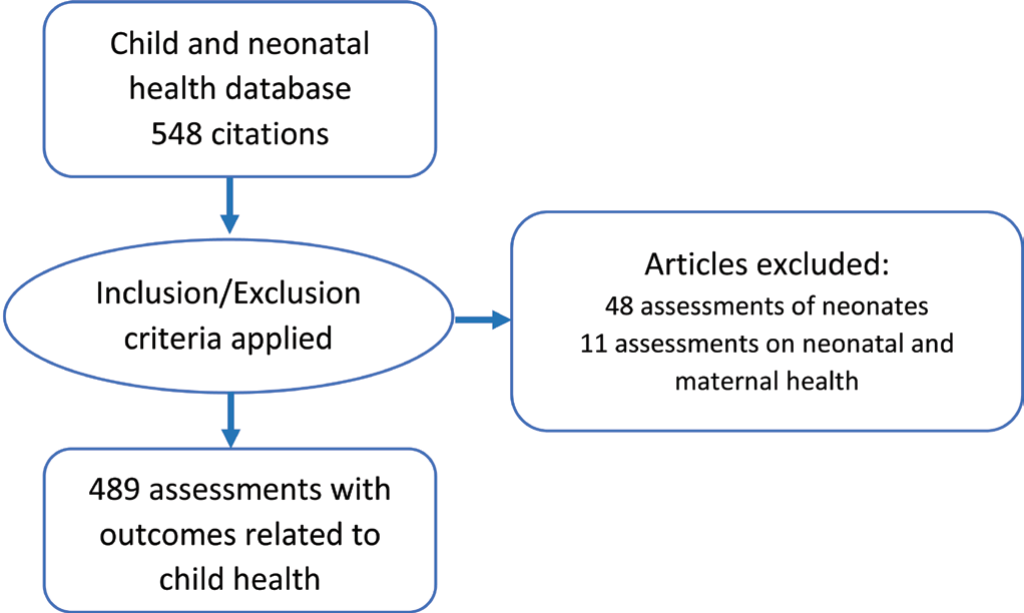
Flowchart of selection of assessments for child health review.

There were 548 assessments included in our database for neonates and 1–59 month–old children. The age of the study population was clearly documented as less than one month in 48 of these assessments. In another 12 assessments the intervention was found to focus on neonatal and maternal health. An analysis of these assessments is reported in the other papers in this series focusing on maternal and neonatal health and not reported here [6,7]. The remaining 489 assessments (**Figure 1**) focused predominately on children beyond the neonatal period, but many also include neonates. The complete bibliography of these assessments in contained in Appendix S1 in **Online Supplementary Document(Online Supplementary Document),** and are indicated in parenthesis with a prefix S throughout this paper.

**Table 1** below lists the most common child interventions described in these 489 assessments. All but 5 of the 129 projects that were classified as providing “primary health care” also implemented one or more of the other interventions shown in **Table 1**. Some categories of child interventions had a relatively small number of assessments and so have been grouped as Other Interventions in **Table 1**. These Other interventions are not analyzed in detail in this paper. Other intervention categories not included above and included in the “Others” group in **Table 1** focused on trachoma prevention, tuberculosis, community organizations, financing, training and use of radios.

**Table 1:** Leading categories of child health interventions included in assessments

Intervention area	No.*	Percentage (n = 489)
Any nutrition–related activity (growth monitoring, breastfeeding promotion, complementary feeding promotion, or provision of micronutrients)	255	52.2
Diarrhea prevention or treatment	183	37.4
Diarrhea prevention and treatment	98	20.0
Diarrhea prevention only	48	9.8
Diarrhea treatment only	30	6.1
Malaria prevention or treatment	150	30.3
Malaria prevention and treatment	91	18.6
Malaria prevention only	27	5.5
Malaria treatment only	11	2.2
Immunizations	132	27.0
Primary health care	129	26.4
Integrated Management of Childhood Illness	110	22.5
Pneumonia prevention or treatment	108	22.1
Pneumonia prevention and treatment	46	9.4
Pneumonia prevention only	19	3.9
Pneumonia treatment only	40	8.2
HIV prevention or HIV/AIDS treatment	42	8.6
HIV prevention and HIV/AIDS treatment	13	2.7
HIV prevention only	24	4.9
HIV/AIDS treatment only	2	0.0
Other	24	4.9

*The sum of this column exceeds 489 since many assessments described more than one intervention.

**Table 2** shows the frequency of assessments according to the number of interventions implemented (not including “primary health care” and counting Integrated Management of Childhood Illness as one intervention). Although half (52%) of the assessments described projects with only one intervention and another quarter (21%) contained only two, one quarter contained three or more.

**Table 2:** Number of intervention category areas among projects that focused on children beyond the neonatal period

Number of interventions per project	Frequency	Percentage (%)
1	243	51.6
2	97	21.3
3 to 4	76	16.6
5 to 7	49	10.5
Projects with interventions categorized as “Other”	24	4.9
Total	489	100.0

Below we provide an analysis of the interventions for children beyond the neonatal period grouped according to the categories listed in **Table 1**. The full list of studies reviewed and referred to in the parentheses in the text below can be found in Appendix S2 in **Online Supplementary Document(Online Supplementary Document)**, where the assessments in our review that are cited here can be identified from the number in brackets in the text.

**Table 3** outlines the types of study methodologies used for these 489 studies. One–half (52%) are controlled studies and one–quarter (26%) are uncontrolled, before–after comparisons. Other types of study methodologies make up the other quarter of assessments. These various study methodologies are spread fairly evenly across the major intervention categorical areas listed in **Table 1** (data not shown).

**Table 3:** Type of study methodology used among child health assessments

Type of study	Frequency	Percentage (%)
Randomized, controlled	177	36.6
Non–randomized, controlled	74	15.3
Uncontrolled, before–after	127	26.3
Case–control, cross–sectional	15	3.1
Cross–sectional	45	9.3
Descriptive	27	5.6
Non–study activity	24	4.3
**Total**	489	100.0

Space limitations prevent us from a detailed analysis of all 489 assessments (as presented in Appendix S1 in **Online Supplementary Document(Online Supplementary Document)**). We focus on those assessments that have the strongest study designs and greatest size of significant effects effects (these are presented in Appendix S2 in **Online Supplementary Document(Online Supplementary Document)**). The remaining assessments in our review had similar effects unless otherwise stated.

### Findings specific to pneumonia and diarrhea

Pneumonia is the leading single cause of under–5 mortality globally, accounting for 18% of deaths [2]. Diarrhea is a major cause of child mortality and morbidity globally and is responsible for 9% of deaths of children younger than 5 years of age [2]. Under the Integrated Global Action Plan for Pneumonia and Diarrhea (GAPPD), actions to address pneumonia and diarrhea are integrated according to a Treat, Protect and Prevent framework [8]. We will follow this framework in presenting our findings.

#### Treat

This part of the framework includes diagnosis, screening, triage and treatment. Our review includes five randomized controlled studies (RCTs) that all showed operationally important and statistically significant reductions in child mortality as a result of community health worker (CHW) treatment of pneumonia with antibiotics – reductions in the range of 13% to 60% [S1–5]. Throughout this article we will be referencing assessments from our database with numbers in brackets, preceded by an S prefix, to distinguish them from the references cited in the list of references at the end of this article. The number in brackets with an S prefix refers to the number of the assessment in Appendix S2 in **Online Supplementary Document(Online Supplementary Document)**. Many other assessments – mainly non–randomized controlled, uncontrolled and case–control studies – also observed significant operationally important decreases in pneumonia–specific mortality in children aged less than 5 years, ranging from 28% to 69% [S6–11]. Two other RCTs demonstrated that CHWs can decrease the clinical severity of pneumonia significantly by treating respiratory infections at the community level through implementing good–quality case management [S12, S13]. Over 20 other studies showed decreases in child pneumonia–specific incidence or mortality but as their pneumonia case management was part of Integrated Management of Childhood Illnesses (IMCI) or Primary Health Care (PHC), they will be discussed under those sections below. Co–trimoxazole was the antibiotic most commonly used by CHWs in these studies.

#### Protect

Under this component are good health practices from birth: exclusive breastfeeding during the first six months of life, adequate complementary feeding, and vitamin A supplementation. Several RCTs demonstrated the efficacy of community–based vitamin A supplementation in reducing pneumonia mortality. In one, vitamin A supplementation decreased pneumonia–specific child mortality by 26% [S14]. In another study, the incidence of pneumonia was decreased through vitamin A supplementation by 44% [S15]. Zinc supplementation and promotion of hand washing provided by CHWs were each also found to significantly decrease the incidence of both pneumonia and diarrhea [S16, S17]. In one randomized controlled trial assessment, a community–based integrated nutrition program apparently not including vitamin A or zinc supplementation demonstrated a decreased incidence of pneumonia [S18]. Studies of vitamin A and zinc supplementation will be presented in more detail under the nutrition section below. Further studies have demonstrated the strong efficacy of zinc supplementation in reducing the incidence, severity and/or duration of diarrheal episodes in children [S19–24].

#### Prevent

This component includes vaccinations, hand washing with soap, safe drinking water and sanitation, reducing household air pollution, HIV prevention and co–trimoxazole prophylaxis for HIV–infected and HIV–exposed children. Education of community members about diarrheal disease was a common activity carried out by trained CHWs, usually by visiting households or meeting with community groups. Randomized controlled trials found that community education focused specifically on the importance of proper disposal of animal feces from living areas produced decreases in the incidence of childhood diarrhea [S25, S26]. Randomized controlled trial assessments of education of caregivers about hand washing along with the provision of soap also decreased childhood diarrhea to an even greater degree than those mentioned in the previous sentence [S27–31]. Teaching mothers to use oral rehydration solution at home along with education about good household sanitation practices – whether by nurses working at the community level or by CHWs – was also effective [S32–34].

Purification of water within the household with sodium hypochlorite or another locally produced purifying agent was found effective in reducing childhood diarrhea in several studies [S35–38]. Solar sterilization of water was demonstrated as an effective approach to decrease the incidence of childhood diarrhea [S39–41]. Water filters such as BioSand and Lifestraw Family Filter that remove particulate matter were similarly effective in reducing the *E. coli* concentration in water and decreasing episodes of diarrhea [S42, S43]. The efficacy of community–based interventions concerning immunizations, HIV and nutrition are presented later in the respective sections.

### Findings specific to malaria

Malaria is one of the three commonest causes of child mortality in those countries where it is endemic. In Africa, malaria is the cause of 15% of under–5 mortality [2]. Major community–based interventions for malaria prevention and treatment include: distribution of insecticide–treated bed nets (ITNs), household residual spraying, antimalarial treatment within the patient’s household (HH) or in the community by CHWs, and intermittent preventive treatment (IPT) of malaria with anti–malarial medication. Community–based diagnosis of cases of malaria by CHWs may be based on clinical signs only or assisted by a rapid diagnostic test (RDT). **Table 4** presents illustrative randomized controlled trials from our database.

**Table 4:** Randomized controlled trails of community–based malaria prevention and treatment projects focusing on children

Intervention	Type of outcome	Population size of study area	Specific outcome	Effect compared to control	Statistical significance	Reference number*
**Distribution of impregnated bed nets with community education:**						
Distribution with education	Mortality	5000–10 000 children in each arm	Mortality among children 1–7 y; mortality among children 1 mo–4 y; all–cause (1 to <5 y) mortality	Decreased by 25%; decreased by 18%; decreased by 33%	0.01; 0.05; 0.01	[S44], [S45], [S46]
Distribution with education	Mortality	2260 children 6 mo to <6 y	Malaria–specific mortality among children 1 to <5 y	Decreased by 30%	0.05	[S47]
Distribution with education	Coverage and mortality	Children in 160 villages	Percentage of children 0 to <5 y sleeping under an ITN; child mortality	Increased by 72%; decreased by 12%	0.01; 0.05	[S48], [S48]
Distribution with education	Coverage and morbidity	Children in 8 villages	ITN coverage to all households; *A. gambiensis* density	Increased by 99%; decreased by 99%	0.001, 0.001	[S49], [S49]
LLITN given plus training given to head of household	Morbidity	Children in 2015 households	Percentage of children 0 to <5 y with malaria	Decreased by 38%	0.05	[S50]
Distribution without education	Morbidity	219 children in 16 villages	Percentage of patients with fever	Decreased by 72%	<0.001	[S51]
Distribution with education (CHW going house to house)	Coverage	1400 children	Percentage of children sleeping under an ITN	Increased by 27%	0.05	[S52]
Community health network to support LLITN distribution	Coverage	11 villages	Percentage of total population using ITN at time of a 6–month follow up	Increased by 32% (in children 0 to <5 y)	0.001	[S53]
Education via CHW at HH level and community women’s groups	Coverage	40 villages	Percentage of total population sleeping under an ITN	Increased by 49%	<0.001	[S54]
**Community and household malaria treatment and prophylaxis:**						
Treatment with chloroquine by mothers	Mortality	5385 children 0 to <5 y	All–cause child mortality	Decreased by 41%	0.003	[S55]
Training CHWs to treat malaria using an RDT	Accuracy of diagnosis	1457 children 0 to 15 y	Percentage of children treated unnecessarily with ACT	Decreased by 45%	0.001	[S56]
CHW treatment of malaria (based on RDT results), with AL (and also treatment with amoxicillin if symptoms of pneumonia present)	Morbidity	11 400 children 6 mo to <5 y	Percentage of febrile children who received AL; percentage of children diagnosed with pneumonia who received early appropriate treatment	Decreased by 77%; increased by 53%	<0.0001; <0.001	[S57], [S57]
CHW treatment of malaria with ACT (and also treatment with amoxicillin if symptoms of pneumonia present)	Morbidity	609 children 4–59 mo	Percentage of children receiving prompt and appropriate antibiotics	Increased by 34%	<0.001	[S58]
HH treatment of malaria (using an RDT) by CHW plus monthly IPT for 3 mo	Coverage of chemo–prophylaxis; morbidity	500 children 1–10 y (one–half also received IPT)	Incidence of RDT–confirmed malaria in HH + IPT group compared with HH– only group; coverage of children by 3 doses of IPT	Reduced by 85% (compared with HH only group); oncreased by 97%	0.01; 0.001	[S59], [S60]
IPT [Sulfadoxine–pyrimethamine at 3,9, and 15 mo (at time of routine immunization)	Coverage of chemo–prophylaxis	600 children 3 mo of age	Protective efficacy during the intervention period (among children 3–18 mo)	Increased by 22%	<0.0001	[S61]

ACT– Artemisinin combination therapy, AL– Artemether–lumefantrine, BCC– behavior change communication, CHW– Community health worker, IPT– Intermittent preventive treatment, HH– Household, ITN– insecticide–treated bed net, LLIN– Long–lasing insecticide–treated bed net, mo – month(s), RDT– Rapid diagnostic test, WAZ: weight–for–age Z score, WHZ – weight–for–height Z score, y – year(s)

*See Appendix S2 in **Online Supplementary Document(Online Supplementary Document)**.

As shown in **Table 4**, there are now a number of randomized controlled trials of community–based interventions for malaria prevention and control that have shown operationally important programmatic effects, with some showing marked mortality impacts. These assessments demonstrate strong evidence of the effectiveness of community–based approaches to the prevention and control of malaria. The interventions presented include use of CHWs involved in house–to–house and group implementation strategies, treatment of malaria within the community by CHWs and mothers, engagement of women’s groups, and malaria control provided by mobile teams from peripheral health facilities.

There were several other assessments that provided evidence in support of the community–based distribution of impregnated bed nets for prevention of malaria [S62–68]. A commonly used approach which produced operationally important outcomes was combining the distribution of ITNs with measles vaccination at the time of mobile clinic outreach sessions [S69–72]. Combining distribution of ITNs with malaria treatment was also effective [S73–75]. Several studies provided evidence that impregnated curtains have some effectiveness in reducing all–cause child mortality [S76, S77]. Some other studies focused on the use of ITNs but did not show as strong evidence individually [S78–83]. Studies which include prevention and treatment of malaria with Integrated Community Case Management of Childhood Illness (IMCI) or with other integrated approaches (such as Care Groups and Primary Health Care) will be presented later in this paper.

The assessments included in **Table 4** above present important aspects of the community–based treatment of malaria. Kidane et al. [S55], by showing that mothers in a remote area of Ethiopia (Tigray) with minimal training could decrease child mortality by diagnosing and treating malaria themselves, illustrated the importance of adapting interventions to local community circumstances as well as the importance of community capacity building. Other studies presented in **Table 4** provide good evidence that CHWs can diagnose and treat malaria in the community in association with the initial management of pneumonia in the same child at the same time [S57, S58]. Several other studies also demonstrated effective treatment of malaria by CHWs in the community alone or in combination with the treatment of concurrent diarrhea or pneumonia [S84–92].

While many of these studies of malaria treatment demonstrated a reduction in malaria–related morbidity or an improvement in CHW performance outcomes related to malaria, some demonstrated important decreases in overall child mortality as well [S93,94]. The cost-effectiveness of combining malaria and pneumonia treatment was studied. However, the findings were inconclusive [S95]. The demonstration of the capacity of CHWs to accurately diagnose malaria using RDTs is also an important finding [S56].

**Table 4** also demonstrates the operational effectiveness of community–level IPT provided by CHWs [S59–61]. Several studies have demonstrated evidence of the important role that other members of the community can play in malaria prevention. School teachers, for instance, can provide IPT with a demonstrable impact on child mortality [S96]. However, the assessment reporting this result, although reporting significant operationally important outcomes, did not provide an adequate description of the intervention and therefore the finding needs to be interpreted with caution.

Trained traditional healers and drug vendors can effectively educate mothers about malaria prevention and early treatment [S97, 98]. Some other studies that focused on malaria treatment or IPT at the community level had results that were consistent with our findings above but the strength of evidence was not as strong [S99–111].

### Findings specific to human immunodeficiency virus infection

There were fewer studies specifically on HIV/AIDS prevention and control at the community level. One study demonstrated that community–level treatment with co–trimoxazole of HIV–infected adults led to a reduction of 77% in the mortality of their originally HIV–negative, under–10 year–old household members. The provision of the drug and the monitoring of activities were provided by community members [S112]. Several studies reported on community–based HIV testing. One study found that among persons taking antiretroviral therapy, contacts that were visited at home were much more likely to undergo HIV testing than persons seen only at the health clinic [S113]. The prevention of mother–to–child transmission (PMTCT) was the most commonly studied HIV intervention in the assessments reviewed. As PMTCT is discussed in our maternal health paper, only a few examples will be mentioned here. In one study, the probability of survival of children to 18 months of age was 84% higher, compared to those in the control group, when HIV–positive mothers received antiretroviral medication as part of a comprehensive integrated program for HIV exposed infants [S114]. Household visits by CHWs, immunizations and growth monitoring were a part of this project.

The role of household visiting by CHWs was often found to be important for HIV–control projects. In one project, intensive follow–up care by CHWs at the homes of HIV–infected mothers led to much greater compliance with PMTCT and also with antenatal and postnatal care. Initiation of anti–retroviral therapy (ART) for HIV– infected infants was also earlier [S115]. Similarly, CHW home visiting was found to lead to a statistically significant 27% increase in identification of HIV–exposed and infected infants and attendance at health facilities [S116]. Community household visits by midwives who gave counseling and nevirapine to HIV–positive mothers and advised them to give nevirapine to their newborns within 72 hours of birth were found to decrease mother–to–child transmission of HIV by 60% [S117]. Community–based adherence support for 982 children on antiretroviral treatment was found to lead to 60% more children achieving virological suppression than children in the control group (*P* = 0.01) [S118].

In many NGO–led child survival projects included in our review, education about HIV/AIDS with or without PMTCT was part of the project, along with many other interventions, and virtually all of them showed marked increases in knowledge about HIV infection.

### Findings specific to immunizations

Immunizations against infectious diseases are well–established as an essential PHC intervention for child health. We have disaggregated the community–based assessments in our database under the areas of activity below.

#### Promotion and uptake through CHWs or others in routine systems

Community–based interventions involving CHWs reaching to the household level to promote participation in immunization activities and CHWs mobilizing communities have played a key role in producing high rates of population coverage for immunizations throughout the world. Peer education provided by CHWs visiting households, by community members recruited just for this purpose, by female community health education workers, and by members of mobile health teams coming from health facilities have contributed to greatly increased immunization coverage rates for children [S119–123].

Establishment of village networks of trained traditional birth attendants and female CHWs was effective. These CHWs promoted immunizations, use of health facilities, and household diarrhea management with oral rehydration solution (ORS) and also carried out growth monitoring of children. Their activities led to a 150% increase in the coverage of 12–23 month–old children with full immunization [S124].

#### Village–level approaches to community mobilization

Promotion of community participation through education of village leaders, teachers, and extension workers (who in turn educated community members) was found effective, increasing full immunization completion coverage levels by 50% [S125]. Mass media using TV, radio, newspapers and leaflets, distributed and explained by community–level workers, significantly increased community awareness about immunizations with mothers. Those mothers who had increased awareness were much more likely to take their children to be vaccinated [S126]. In Lao PDR, community–based workshops promoting attendance for vaccination significantly increased all childhood vaccinations [S127].

#### Promotion of immunizations through microcredit programs

A case–controlled study of community health education campaigns associated with microcredit programs were found to greatly increase fully immunization coverage [S128].

#### Health Days

National Immunization Days, in which community mobilization and immunization at peripheral service points followed up by immunization at the household for those who did not come to the service point led to significant decreases in the incidence of acute flaccid paralysis [S129]. Annual vaccination weeks with household visits by CHWs increased vaccination completion rates from 30% to 53% [S130].

#### Household vaccination strategies

A case–controlled study of peer education provided by CHWs visiting households, promoting community involvement, and providing immunizations, vitamin A supplementation and growth monitoring led to not only to greatly increased immunization coverage but also to a 58% decrease in under–5 mortality compared to controls [S131]. House–to–house administration of polio vaccine significantly increased polio vaccination rates [S132].

### Findings specific to nutrition

Undernutrition contributes to 45% of under–5 mortality globally [3] and therefore is a major concern. In this section our review findings will be categorized into four areas: protein–energy undernutrition (usually assessed by anthropometry), breastfeeding (BF), complementary feeding (CF), and micronutrient supplementation.

#### Protein–energy undernutrition

**Table 5** presents the findings from randomized controlled and non–randomized controlled studies with statistically significant and operationally large effects compared to controls with protein–energy undernutrition. **Table 5** demonstrates that undernutrition can be addressed successfully at the community level through health education involving CHWs visiting households, regular monitoring of child growth in the community, and supplementation with ready–to–use therapeutic food (RUTF). Albendazole supplementation to mothers also was found to have an important effect on child growth. Even for depressed mothers with HIV, well–organized programs improved the nutrition of their children. Group learning programs associated with small loans (that may have enabled mothers to obtain more nutritious foods for their children) also improved child nutrition. Many other integrated programs were also demonstrated to contribute to good child nutrition. These will be covered below in the final section on integrated programs.

**Table 5:** Studies of community–based interventions addressing protein energy undernutrition

Intervention	Type of outcome	Population size of study area	Specific Outcome	Effect compared to control	Statistical significance	Reference number*
**Randomized controlled assessments:**						
Home–based distribution of RUTF for children with severe acute, malnutrition	Change in nutritional Status	1178 10–60–mo–old malnourished and wasted children	Attainment of WHZ≥2 without edema or relapse	Increased by 33%	0.001	[S133]
Education plus micronutrient–fortified milk–based cereal household supplementation	Change in nutritional status	104 infants each in 3 different groups [Supplementation only, counselling only, and control)	Percentage of children with a mean weight gain of 250 g or more	14% more (in supplemental group compared to control group)	0.01	[S134]
Nutrition and hygiene education with growth monitoring at community level	Change in nutritional status	Children 0 to <5 y from 55 randomly selected households	Mean WAZ in older children, mean WAZ in younger children	Increased by 10%; Increased by 36%	0.05; 0.001	[S135]
Albendazole 600 mg every 6 mo provided at household level	Change in nutritional status; morbidity	610 children 18 mo of age who were treated for two years	Prevalence of stunting; prevalence of fecal worms	Decreased by 9%; Decreased by 14%	0.001; 0.001	[S136]
Home visits by CHWs to reduce alcohol use, promote BF, child nutrition, and perinatal HIV regimen compliance	Change in nutritional status	644 depressed mothers and their children 0 to<6 mo	Mean LAZ scores for children 0 to <6 mo	Increased by 7%	0.034	[S137]
Paraprofessional home visits with provision of health education about BF, child nutrition, HIV, PMTCT, and mental health	Change in nutritional status	24 township neighborhoods	Mean WHZs for children	Increased by 19%	0.001	[S138]
**Non–randomized controlled interventions:**						
Home visits from community health agent facilitators to provide education and monthly growth monitoring	Change in nutritional status	14 374 children, 0 to <5 y	Undernutrition in children 0–35 mo	Decreased by 27%	0.05	[S139]
Albendazole 400mg distributed to households with mothers at 12 and 23 weeks of pregnancy	Change in nutritional status	4998 mothers and their children, 0 to<6 mo	Mortality rate in infants during their first 6 mo of life	Decreased by 41%	0.01	[S140]
Using CHWs in a nutritional demonstration (Hearth) program (mothers are trained by participation in cooking nutritious food for children)	Change in nutritional status	1200 children, 3–48 mo	Percentage of children with normal weight for age; percentage of children with severe undernutrition	Increased by 10%; decreased by 18%	0.02; 0.02	[S141], [S141]
Facilitated group learning sessions on maternal and child health with small loans given to mothers	Change in nutritional status	200 children 0 to<3 y	Mean HFA children 12 to 24 mo	Increased by 48%	0.01	[S142]

BF – breastfeeding, HFA – height for age, HIV – human immunodeficiency virus, LAZ – length–for–age Z score, mo – month(s), PMTCT – prevention of mother–to–child transmission, RUTF – ready–to–use–therapeutic food, WHZ – weight–for–height Z score, y – year(s)

*See Appendix S2 in **Online Supplementary Document(Online Supplementary Document)**.

Other controlled interventions with smaller effect sizes and statistically significant results at the *P* < 0.05 level also were very informative. In Vietnam, among children aged less than 15 months with a weight–for–age Z score of <–2, the Hearth approach along with de–worming significantly improved growth when compared to controls who received only deworming [S143]. The Hearth approach is a process of identifying local “positive deviant” women who have well–nourished children. Mothers of malnourished children are also identified and they are guided through a process of learning how positive deviants care and feed their children and applying this knowledge in the care of their own children through hands–on cooking sessions using locally available foods [S143].

In a non–randomized controlled project that was implemented over a five–year period, the hypothesis was tested that younger siblings of older children with severe undernutrition whose undernutrition had been overcome using the Hearth approach should have better nutrition than similar children whose mothers had not been exposed to the Hearth program. Outcomes were compared for 10 different 3–month age groupings of younger siblings (6–8, 9–11, 12–14, etc.). For younger siblings whose older sibling had been severely malnourished and whose mother had been exposed to the Hearth approach (the intervention group), the younger sibling mean weight for age Z score was always higher than the older sibling (*P* = 0.005 or less in all age groups). For the control group (children with an older sibling who had been moderately malnourished, mildly malnourished, or of normal weight and whose mother had not been exposed to the Hearth program) the same comparison with younger siblings was carried out. The mean weight for age Z score of the younger siblings was always lower for mildly malnourished and normal weight children than their older sibling (*P* < 0.05 for all but one age group, 6–8 months). This study provides evidence regarding the wider family effects of nutritional education [S144]. However any conclusions need to be guarded due to the limited size of the populations studied. These results would need to be repeated in further similar studies.

The benefits of promotion of agriculture and voucher programs on childhood nutrition have also been demonstrated. In a population including 130 000 children younger than 5 years of age in Nepal, promotion of increased household production of food through training Village Model Farmers, and subsequently village women, led over a 2–year period to a decrease of 10% in the prevalence of underweight in children aged 0 to 4 years [S145]. A community development and livestock promotion project in Nepal for 307 children produced similar results. Although the results in the latter study were not statistically significant after 2 years, the intervention group was more likely to have indoor access to water, treat their water and have a latrine. Longer participation in the program was strongly associated with a better mean height–for–age score (*P* < 0.00001) [S146].

Giving vouchers to mothers along with health education and a community household health package was found not to result in statistically significant improved child nutrition in the short term but if the program for those children was extended for 2 years more until the children were aged 8 to 10 years, then the mean height for weight Z scores of these children increased by 23% (*P* = 0.029) compared to controls of the same age [S147]. Other studies demonstrated a statistically significant association of mothers receiving vouchers with greater use of nutrition monitoring at the community level and improved nutrition of their children [S148–151].

#### Breastfeeding and complementary feeding

Exclusive breastfeeding (BF) during the first 6 months of age with continued BF through the first two years of life is an important contributor to good childhood nutrition, reduced morbidity, and improved mortality in resource–constrained settings. Promotion of exclusive breastfeeding for the first 6 months of life has been estimated to be one of the most effective preventive strategy for saving the lives of young children in low–income settings [9]. Complementary feeding (CF) to supplement breastfeeding is needed from 6 months of age onwards for children to sustain normal growth. Findings from randomized and non–randomized controlled community–based assessments included in our review are presented in **Table 6****.**

**Table 6:** Community–based projects that promoted breastfeeding and complementary feeding in children

Intervention	Type of outcome	Population size of study area	Specific outcome	Effect compared to control	Statistical significance	Reference number*
**Randomized controlled interventions**						
Breastfeeding:						
Training of 1 CHW per village to promote exclusive BF	Change in health–related practice	1115 mothers and their children 0 to <6 mo	Percentage of children exclusively breastfed to <6 mo of age	Increased by 38%	0.05	[S151]
Home counselling by trained CHWs	Change in health–related practice	1597 mothers and their children, 0 to <6 mo	Percentage of children exclusively breastfed to <6 mo of age	Increased by 63%	0.001	[S152]
Home visits by trained women during the postnatal period	Change in health–related practice	175 mothers and their children 0 to <6 mo	Percentage of children exclusively breastfed to <6 mo of age	Increased by 16%	0.001	[S153]
Peer counsellors from community educated pregnant mothers in breastfeeding	Change in health–related practice	726 pregnant women and their children 0 to <6 mo	Exclusive breastfeeding, to <6 mo of age	Increased by 64%	0.01	[S154]
Complementary feeding:						
CHW education of mothers about CF during home visits	Change in nutritional status	118 infants	Prevalence of stunting	Decreased by 10%	<0.05	[S155]
**Non–randomized controlled trials:**						
Training of mothers in essential nutrition by community outreach workers	Change in health–related practice	320 infants 0 to <6 mo in 8 districts	Percentage of children exclusively breastfed until 6 mo of age	Increased by 22%	0.001	[S156]
Provision of fortified CF at households along with education by CHWs	Change in nutritional status	Children 9–14m in the catchment areas of 10 health clinics	Odds of being underweight after being enrolled in the program for one year	Decreased by 75%	0.007	[S157]
**Uncontrolled before–after studies:**						
Formation of community health clubs and provision of health education by CHWs	Change in health–related practice	1000 children 0 to <5 y and their mothers	Early initiation of BF; Exclusive BF in children 0–6 mo	Increased by 50%; increased by 60%	0.001; 0.001	[S158], [S158]
Hearth program, CF education by CHWs, nutrition revolving fund established to aid mothers to buy chickens to provide protein for children plus small income	Change in nutritional status	1700 children 0 to <3 y	Prevalence of normal WFA children; prevalence of severe malnutrition	Compared to baseline, increased by 13%; decreased by 17%	0.001; 0.001	[S159], [S159]

BF – breastfeeding, CF – complementary feeding, CHW – community health worker, mo – month(s), WFA –weight for age, y – year(s)

*See Appendix S2 in **Online Supplementary Document(Online Supplementary Document)**.

The data from **Table 6** indicate that exclusive breastfeeding can be effectively promoted at the community level by CHWs, by trained home peer counsellors, by community outreach health professionals from the nearest health facility, and by mothers’ community health clubs. Of note is that the strongest effects were found when the CHWs and home peer counselors rather than more highly trained health professionals reaching out from local health facilities were doing the education. Education about complementary feeding was found to produce statistically significant improvements in mean height and weight. The Hearth approach mentioned in the section on protein energy undernutrition was also found to be effective in undernourished children younger than 15 months of age, in the study cited in **Table 5** [S141], and in other studies with similar results [S143, S159, S160].

#### Micronutrient supplementation

Types of micronutrient supplementation that were included in projects whose assessments qualified for our review included vitamin A, zinc, iron and multivitamins. **Table 7** contains details about randomized and non–randomized controlled studies that have been included in this review and that have operationally important effects.**Table 7** shows that vitamin A supplementation provided at the household level to mothers, to newborns, and especially to children 6–59 months of age leads to decreased child mortality. Even fortifying market monosodium glutamate with vitamin A leads to a decrease in the rate of xerophthalmia (a condition of eye dryness and eventual scarring produced by vitamin A deficiency) and all–cause child mortality. It also decreases child mortality from pneumonia and measles.

**Table 7:** Studies of micronutrient supplementation at the community level

Intervention	Type of outcome	Population size of study area	Specific outcome	Effect compared to control	Statistical significance	Reference number*
**Randomized controlled interventions:**						
Vitamin A supplementation:						
Supplemental vitamin A 8333 IU weekly and E at the household level	Mortality	7764 children, 0 to <5 y	Risk of death in girls; risk of death in boys	Decreased by 59%; Decreased by 48%	0.01; 0.04	[S161], [S161]
Maternal vitamin A 3330 IU daily and folate supplementation	Mortality	3389 pregnant women and children	Perinatal, and neonatal mortality	Decreased by 20%	0.01	[S162]
Vitamin A (200 000 IU for 12–59 mo–old children, 100 000 IU for 6–11 mo–old children, and 50 000 IU –5m) in a single dose	Mortality	3786 children, 0 to <5 years	1–59 mo mortality	Decreased by 26%	0.05	[S14]
Vitamin A every 4 mo (60 000 IU)	Mortality	28 630 children, 6–72 mo	1–59 mo mortality; case fatality rate for measles	Decreased by 30%; decreased by 76%	0.05; 0.001	[S163], [S163]
Vitamin A 200 000 IU every 6 mo for 18 mo	Morbidity	12 109 children, 9–72 mo	Incidence of night blindness	Decreased by 50%	0.001	[S164]
Vitamin A 200 000 IU for 12–59 mo–old children and 100 000 IU for 1–11m–old children every 4 mo	Mortality	9200 children, 0 to <5 y	1–59 mo mortality	Decreased by 19%	0.05	[S165]
Vitamin A 60 000 IU every 4 mo	Mortality	28 630 children, 6–72 mo	1–59 mo mortality in females	Decreased by 90%	0.0001	[S166]
Vitamin A 200 000 IU for 1–3 mo–old children at 1–3 mo of age and again 6–8 mo later	Mortality	25 000 children, 0 to <5 y	1–59 mo mortality	Decreased by 34%	0.01	[S167]
Infants received 24 000 IU of vitamin A on days 1 and 2 after delivery	Mortality	5786 newborns	Mortality during the 1st 6m of life	Decreased by 22%	0.02	[S168]
Vitamin A given at birth (50 000 IU)	Mortality	7953 newborns	All–cause infant mortality	Decreased by 15%	0.045	[S169]
Vitamin A 200 000 IU for 12–59 mo–old children and 100 000 IU for 1–11 mo–old infants	Morbidity	1405 children, 6–47 mo	Incidence of acute respiratory infection in normal children.	Increased by 8%	0.05	[S170]
Vitamin A 200 000 IU for 12–59 mo–old children and 100 000 IU for 1–11 mo– old infants twice a year and accompanied by nutrition education	Change in nutritional status	720 children 0–36 mo	Prevalence of stunting	Decreased by 11%	0.01	[S171]
Zinc supplementation:						
Vitamin A 200 000 IU as one dose plus 10 mg zinc 6 days a week	Morbidity	148 children, 6–72 mo	Prevalence of malaria	Decreased by 32%	<0.001	[S172]
Zinc (70 mg) weekly for one year	Morbidity	809 children, 6–18 mo	Incidence of pneumonia	Decreased by 44%	0.01	[S83]
Daily supplementation with 10 mg of zinc	Mortality	21 274 children, 12–48 mo for 485 days	Relative risk of all–cause mortality in children 12–48 mo	Decreased by 18%	0.045	[S173]
Daily supplementation with 10 mg of zinc	Morbidity	854 children 6–48 mo	Incidence of diarrhea in children 0 to < 2 y	Decreased by 25%	0.001	[S174]
Zinc 20mg zinc daily for 15 d (for children with diarrhea)	Morbidity	139 children 6–35 mo	Duration of persistent diarrhea	Decreased by 28%	0.01	[S175]
Iron supplementation:						
Iron, folate and zinc supplementation: iron (12.5 mg), folic acid (5 µg) zinc (10mg) daily	Morbidity	Children, 1 to <6 mo	Risk of severe morbidity (from severe malaria) and death in groups that received iron	Increased by 12%	0.02	[S176]
Sale to households of “Sprinkles” (a powder to sprinkle on top of food) containing iron and B vitamins	Morbidity	561 children, 0 to <5 y	Prevalence of anemia	Decreased by 19%	0.001	[S177]
Daily home fortification with micronutrient powder containing iron for 2 mo	Change in nutritional status	1103 children, 0 to <5 y	Mean hemoglobin concentration	Increased by 7%	0.001	[S178]
Multivitamin and mineral powder (MMP) supplement: 2 sachets 2 times a week (compared to 2 sachets MMP daily and controls)	Morbidity	115 children, 0 to <5 y in each of the 3 groups	Prevalence of anemia, compliance with MMP supplement	Decreased by 32% in daily MMP; 200% greater in 2 times a week group compared to daily	0.001; 0.001	[S179]
**Non–randomized controlled interventions:**						
Vitamin A supplementation:						
Fortification of monosodium gluconate sold in markets with vitamin A	Morbidity	5755 children 0 to <5 y	Prevalence of Bitot’s spots; mortality	Decreased by 600%; mortality rate among pre–school children in the control villages was 1.8 times greater than that for children in intervention villages	0.0001; 0.001	[S180], [S180]
Education on weaning practices, Vitamin A provision to children, Provision of iron to mothers, immunizations, door–to–door visits from CHWs	Mortality	6663 children, 0–35 mo and 14 551 women	All–cause mortality among children 6–35 mo; pneumonia–specific mortality among children 6–35 mo	Decreased by 32%; decreased by 53%	0.001; 0.001	[S181], [S181]

Daily zinc supplementation decreased all–cause mortality in children 12–48 months of age, but not to the same extent as vitamin A. A decrease in the incidence of diarrhea in children receiving zinc has also been demonstrated in other controlled studies [S174, S175]. Of particular note is that in one study of children 1 to <6 months of age in a malaria–prone area, the risk of death or severe morbidity increased significantly in those who received iron supplementation [S176]. While other studies in non–malaria–endemic areas confirmed the value of iron supplementation for treating anemia, this finding provides reason for caution in providing iron supplementation to children aged 1 to <6 months of age in malaria–endemic areas.

### Findings specific to integrated approaches to child health

Children present with a variety of common diseases even when one disease such as malaria may predominate in a particular area. Undernutrition is a common risk factor for childhood infections [10,11]. Opportunities to update immunization status need to be taken at every opportunity to prevent serious childhood infections. Mothers may lose confidence in CHWs and CHWs may lose confidence in themselves if CHWs have to turn patients away because they can only deal with one disease entity (or if they do not have the capacity to treat any illnesses). Therefore, for the most cost–effective and efficient use of resources and for increasing the confidence of mothers in CHWs and CHWs in themselves, it is important that services provided be integrated as much as practical for the benefit of all. To do this, a range of integrated approaches have been developed at the community level, and available assessments of the projects have been included in our review.

#### Integrated Management of Childhood Illness (IMCI) and Integrated Community Case Management (iCCM)

Integrated Management of Childhood Illness (IMCI) integrates the prevention and treatment of all childhood illness at health facilities. Its community component, called Community IMCI (or C–IMCI), usually consists of preventive activities and early recognition of potentially serious acute illness that can be performed in the community by trained CHWs going door–to–door and meeting with groups, usually without treatment of illnesses other than ORS for diarrhea. CHWs are taught to recognize children with danger signs and refer or even escort patients to the nearest health facility for treatment. CHWs also facilitate outreach activities from the local health center such as immunizations.

Integrated Community Case Management (iCCM) enables CHWs to diagnose and treat serious acute illnesses of childhood (acute respiratory infection, diarrhea, malaria and in some cases acute malnutrition).

For iCCM to be effective, CHWs need to be well–trained, to have the confidence and support of their community, to be well–linked to their local health facility staff for referral of patients, to receive regular supervision to maintain their skills, and to be well–supplied with the drugs and equipment necessary to perform their tasks [12]. These CHWs often also have community health education roles, perform household visiting, and may also be responsible for such activities as promotion and distribution of ITNs. Studies of IMCI and iCCM are often concerned with maintaining the quality of all the above tasks. **Table 8** summarizes the findings of assessments of C–IMCI and iCCM interventions.The studies described in **Table 8** show that iCCM can be implemented successfully at the community level and indeed may lead to a decrease in under–5 mortality. A large assessment of children younger than 5 years of age in 15 districts in Rwanda with complete mortality data further supports this. This assessment found that the number of children receiving community–based treatment for diarrhea and pneumonia increased significantly in the 1–year period after iCCM implementation, from 0.83 cases/1000 child–months to 3.80 cases/1000 child–months (*P* < 0.001) and from 0.25 cases/1000 child–months to 5.28 cases/1000 child–months (*P* < 0.001), respectively. On average, total under–5 mortality rates declined significantly by 38% (*P* < 0.001), and health facility use declined significantly by 15%. These decreases were significantly greater than expected based on baseline trends [S192].

**Table 8:** Studies of the effectiveness of Community–Integrated Management of Childhood Illnesses (C–IMCI) and Integrated Community Case Management (iCCM)

Intervention	Type of outcome	Population size of study area	Specific outcome	Effect compared to control	Statistical significance	Reference number*
**Randomized controlled trials:**						
CHWs trained as part of the family and community activities associated with IMCI, as well as health system strengthening	Mortality; change in nutritional status	The catchment areas of 10 health facilities (175 000 persons)	All–cause mortality 0 to <5 y; prevalence of exclusive breast feeding 0 to <6 mo	Decreased by 13.4%; Increased by 10.1%	0.01; 0.05	[S182]
**Non–randomized controlled trials:**						
Linkage of CHWs with local health facilities and provision of training to CHWs	Coverage; change in nutritional status	Children 0 to <2 y in a population of 160 000	Percentage of children 12–23 mo fully immunized; percentage of children receiving at least five meals per day	Increased by 21%; increased by 32%	0.05; 0.05	[S183]
Awareness seminars conducted during the first year for leaders of all villages followed 1 y later by similar seminars for extension workers and teachers	Coverage; change in nutritional status	Women of child–bearing age and their children in villages with a total population of 18 000	Percentage of children with full immunization coverage; percentage of children with severe undernutrition	Increased by 50%; decreased by 27%	0.001; 0.05	[S184]
CHWs trained in iCCM	Mortality	Children <5 y in villages with a total population of 14 000	Under–5 mortality	Decreased by 38%	0.003	[S185]
On–site monthly supervision on C–IMCI by trained supervisors of Health Extension Workers (HEWs)	Quality of care	500 HEWs assessed	Quality of case management over two years (percentage of cases that were correctly classified, treated, and followed–up within two days of initiating treatment)	Increased by 200%	0.04	[S186]
C–IMCI with 2 HEWs working at a community health post	Quality of care	87 HEWS	Correct prescription of anti–malarial medications in comparison to HEWs working in a vertical malaria control program	Increased by 10%	0.05	[S187]
Drug sellers trained in iCCM protocols	Quality of care	Sick children who made 7667 visits to 44 trained drug sellers	Correct treatment of common illnesses	Increased by 27%	0.001	[S188]
Peer support groups among CHWs trained in iCCM	Coverage	1575 children in 6 districts	Number of sick children treated for ARI, malaria, and diarrhea (compared to CHWs trained in iCCM without peer support groups)	Increased by 167%	0.001	[S189]
CHWs trained in iCCM	Coverage	306 190 children 6 mo to <5 y	Number of sick children treated for ARI, malaria, diarrhea	Increased by 23%	0.05	[S190]
CHWs trained in iCCM	Coverage	38 009 children <5 y	Percentage of children sleeping under ITNS	Increased by 33%	0.01	[S191]

ARI – acute respiratory infection, HEW – health extension workers, ITN – insecticide–treated bed nets, mo – month(s), y – year(s)

*See Appendix S2 in **Online Supplementary Document(Online Supplementary Document)**.

In many parts of rural Uganda with limited access to trained health staff, up to 50% of cases of childhood illnesses are managed by drug sellers. One study in which private drug sellers were trained to treat patients using iCCM protocols revealed a strong adherence to the iCCM protocol in terms of testing, examining and treating children. On follow up evaluation after training, 88% of children diagnosed with diarrhea received ORS. 88% of children presenting with a fever received a RDT for malaria and 94% of children who were diagnosed as RDT–positive received artemisinin combination therapy. Of those who were diagnosed with pneumonia, 91% of them received amoxicillin treatment. Overall performance (defined as correct treatment) showed a 27% (*P* = 0.001) increase compared with baseline levels [S188]. The other studies cited in **Table 8** demonstrate that monthly community–level supervision by trained supervisors from the local health facility can lead to maintenance of CHW skills in iCCM diagnosis and treatment and that iCCM leads to more children receiving treatment for these common illnesses [S186, S189–191].

#### Care Groups

Care Groups were included in the review through the publication of the results of the evaluation of several projects. A Care Group is a group of 10–15 community volunteers who act as community–based health educators. The Care Group meets every two weeks with a project facilitator for two hours or so to learn some new education messages. Each volunteer is responsible for regularly visiting 10–15 of her neighbors, sharing the new messages they just learned. With this structure and basic approach, scaling up is readily possible [13,14].

In a 5–year Care Group project in Sofala Province in Mozambique, the project area was divided into two sub–areas (A and B) since project activities began several years later in Area B after activities in Area A had begun. Major improvements were achieved across most indicators of child health comparing baseline with endline findings. Key outcomes were that the overall proportion of children with undernutrition (WAZ<–2.0 SD) decreased by 6% in Area A and by 10% in Area B; insecticide–treated bed net (ITN) use increased by 45% in Area A and by 71% in Area B; rates of exclusive breastfeeding increased by 60% in Area A and 25% in Area B; the percentage of children 9–23m of age who ate three or more meals per day increased from by 42% in Area A and by 20% in Area B. Based on findings obtained with the Lives Saved Tool (LiST), the project saved an estimated 6848 lives and the cost per life saved, the cost per disability–adjusted life year (DALY) averted, and the annual cost per beneficiary were US$ 441, US$ 14.72 and US $2.78, respectively [S193].

Another Care Group project in the rural part of the Chokwe District in Mozambique also incorporated a community–based vital events registry system as part of the activities of the Care Groups. The assessment of this project demonstrated not only the efficacy of Care Groups but also the quality of a community–based vital events registration system. This assessment demonstrated that the Care Group approach resulted in a 49% decrease in the infant mortality rate and a 42% decrease in the under–5 mortality rate over the five year period of project implementation, confirmed by an independent retrospective morality assessment based on maternal birth histories [S194]. Similar results were found in another Care Group project in our database in Burundi [S195].

#### Integrated community–based primary health care (CBPHC)

Primary health care (PHC) includes the provision of a comprehensive range of essential preventive and treatment actions aimed at meeting all the common health needs of community members (especially those of women of childbearing age and children but also of men and older women) using practical and affordable approaches. For integrated CBPHC to be effective at the community level outside of health facilities, CHWs need to have good linkages to the local health facility to which patients with severe illness, injuries and uncommon or more severe illnesses can be referred and where mothers can give birth. Services such as immunizations that require outreach from health facilities also need to be provided at the community level in order to make essential services readily available. Our review includes a number of community–based PHC programs that are presented in **Table 9**.

**Table 9:** Primary health care programs that have strong community–based components

Intervention	Type of outcome	Population size of study area	Specific outcome	Effect compared to control	Statistical significance	Reference number*
Randomized controlled assessments:						
PHC with full range of child health services provided by CHWs plus outreach services.	Change in nutritional status	788 children 6–23 mo	Height–for–age Z score, Weight–for–age Z score	Increased by 24%, increased by 14%	0.018, 0.05	[S196]
PHC nurses posted in communities without CHWs	Mortality	2000 children <5 y	Under–5 mortality	Decreased by 54%	0.05	[S197]
PHC promoting community involvement with volunteer CHWs and well–trained Community Health Officers	Mortality	51 407 children <5 y	Mortality of children exposed to intervention for more than 2 y	Decreased by 60%	0.001	[S198]
PHC with full range of child health services provided by CHWs plus outreach services	Mortality	6663 children 0–35 mo, 14 551 women	All–cause mortality in children 6–35 mo. Pneumonia– specific mortality in children 6–35 mo	Decreased by 32%. Decreased by 53%.	0.001	[S199]
Non–randomized controlled assessments:						
Census–based PHC with frequent visits by CHWs to all households, distribution of vitamin A, provision of growth monitoring, education, immunizations, and transport assistance when referral needed	Mortality	15 406 (total population of intervention area)	All–cause under–5 mortality	Decreased by 52%	0.001	[S200]
Peer education, referral, and promotion of community involvement in planning, implementing, and evaluating services provided by volunteer CHWs	Mortality	36 000 children <5 y	All–cause under–5 mortality	Decreased by 58%	0.0001	[S201]
PHC with outreach, health education, supplemental feeding, immunizations, curative treatment, TB control, support of TBAs	Mortality	2700 children aged 0–6 y	All–cause under–5 mortality; stunting	Decreased by 67%; reduced by 28% in children 48–59m	0.0001, 0.001	[S202], [S203]
PHC provided at a health center with community outreach by trained health assistants	Mortality	887 persons in health center catchment area	Crude mortality of all age groups over a time period of 10 y until 1951	Decreased by 24%	0.001	[S204]

CHW – community health worker, mo – month(s), PHC – primary health care, TBA – traditional birth attendant, y – year(s)

*Appendix S2 in **Online Supplementary Document(Online Supplementary Document)**.

**Table 9** demonstrates that primary health care with strong community–based components can decrease under–5 mortality. Promotion of community involvement and training/deployment of CHWs is also shown to be a recurring element of these successful programs. Assessments S196–198 are three studies from the Navrongo experiment in Ghana. In the Navrongo experiment in Ghana, there were four groups compared: (1) community health nurses alone–called Community Health Officers, (2) community volunteers and community mobilization without community health nurses; (3) both community health nurses and community volunteers with community mobilization, and (4) a control group. The group that only had community volunteers did not reduce child mortality but did significantly improve child nutrition [S196]. The community–based nurses provided curative care and were effective in decreasing child mortality but did not improve child nutrition or contraceptive coverage [S197]. The best results were achieved when nurses worked with community volunteers and mobilized community members improving child mortality, child nutrition and contraceptive use, together with a 15% improvement in contraceptive coverage [S198].

The census–based, impact–oriented (CBIO) methodology includes mapping and community registering to ensure that all beneficiaries are documented and included in the project information system so that they are included in all community–based PHC programs [S200, S201]. The CBIO approach was pioneered in Haiti in the 1970s. Assessment by retrospective maternal birth histories and household anthropometric surveys demonstrated a 68% reduction in under–5 mortality and reduced prevalence of stunting compared to national rural indicators [S202, S203].

The last assessment in **Table 9** is the earliest one in our database and was reported in 1951 [S204]. It was carried out at a time when there had not yet been many experiences with CHWs and when CHWs were used only for health promotion and referral for provision of health services at a health center.

One important study in our database that does not lend itself to incorporation into **Table 9** is the Narangwal Project, which pioneered many elements of CBPHC [S205]. It operated from 1967 to 1973 in the rural Punjab of Northern India. The nutrition and health–care aspects of this study are of direct relevance to CBPHC and child health. There were four cells in the nutrition aspect of this non–randomized controlled study: (A) a nutrition–only cell, (B) a health–care–only cell, (C) a combined nutrition–and–health–care cell, and (D) a control cell (in which routine government services without outreach were provided). Promotion of community participation was a key aspect of the design of this study. Each cell contained approximately 200–300 children. Child nutrition services included growth monitoring and promotion as well as food supplementation twice daily. The child health care services included infectious disease surveillance and early treatment, immunizations, and education concerning disease prevention. In the nutrition study, mortality rates were significantly reduced during the perinatal, neonatal, post–neonatal, and 12–23 month age groups in both the nutrition cell as well as in the nutrition + health care cell compared to the control cell. In addition, the weight–for–age and height–for–age of children beyond 17 months of age were significantly greater in the nutrition cell and in the nutrition + health care cell compared to control cell [S205].

Key CBPHC aspects of this project were that Family Health Workers provided treatment in the home for dehydration from diarrhea and for childhood pneumonia. The children 0–3 years of age with pneumonia who were treated with penicillin had a 42% reduced risk of overall mortality [S206]. Other key findings based on a qualitative review of data were that: one–on–one education of mothers was essential for improving practices related to breastfeeding, infant feeding, rehydration and feeding of sick infants and also for overcoming traditional beliefs about not feeding a child with diarrhea; weekly home visits were necessary in order to achieve a reduction in infant mortality; delegation of services as far to the periphery as possible improved coverage and effectiveness; rehabilitation of malnourished children through special feeding programs was best accomplished at home or near the home; having a curative health care service was an essential element of building trust, and developing a quality health care program required active community participation and building trust with the community [S207].

Several other assessments included in our database are of particular note since they document the evidence of the long–term benefits of CBPHC projects on child health. These projects are:

- The ICDDR,B MCH–FP Program in Matlab, Bangladesh (a maternal/child health and family planning research field site for the International Centre for Diarrheal Disease Research, Bangladesh/Centre for Health, Population and Nutrition);
- The Hôpital Albert Schweitzer in Deschapelles, Haiti;
- The Jamkhed Comprehensive Health Project in Jamkhed, India; and,
- SEARCH (Society for Education, Action and Research in Community Health) in Gadchiroli, India.

These projects are discussed in detail elsewhere in this supplement [15].

## DISCUSSION

This review provides strong evidence that overall the major causes of child mortality in developing countries can be addressed at the community level outside of health facilities by working with communities and community–level workers. For all categories of interventions, we have presented findings from randomized and non–randomized controlled trials in our database that have consistently produced statistically significant and operationally important effects. In many cases the outcomes observed have been changes in the most objective and meaningful indicator: mortality.

Some assessments, mostly unpublished child survival project evaluations, relied on before/after study designs without a comparison group, measuring changes in population coverage of key child survival interventions. In virtually all cases, the changes in coverage over a 4–5 year period were quite pronounced, particularly in comparison to much smaller changes in coverage in the regional or national population, as a review of a set of these projects has demonstrated [16]. They have generally produced statistically significant and operationally important results. Other less rigorous assessments of the effectiveness of CBPHC in improving child health were not included in this article due to space limitations, but they also provide evidence supporting our major findings presented here.

Our findings regarding the effectiveness of specific community–based interventions for improving child health are similar to those reported in other reviews [4,17]. The provision of iron to children in malaria–endemic areas, whether through community–based approaches or otherwise, may have harmful effects so it not recommended at this time. However, this is the only evidence we have identified in which implementation of CBPHC intervention led to a less than favorable effect. However, it is important to note that this finding pertains to the biomedical interaction of iron on children exposed to malaria rather than on the effectiveness of CBPHC as a strategy for improving child health. At the community level the total number of interventions being implemented – even in a comprehensive primary health care approach – may be spread amongst several CHWs working in a team each of whom may do only one or two interventions. Consequently, evidence about the effectiveness of one or two interventions implemented by individuals, usually CHWs, is consistent with “community–based primary health care.”

We have not addressed here three important questions: (1) who are the community–level workers who implemented the interventions, (2) what particular resources do they need in order to deliver the interventions, and (3) what are the conditions that would need to be met in order to scale up these interventions under routine conditions. Answering these questions is beyond the scope of this paper, and few assessments really address these questions, unfortunately. The degree to which the assessments included here represent efficacy studies (that is, project implementation under ideal field conditions) as opposed to effectiveness studies (implementation under routine field conditions) cannot be adequately explored here. However, it is clear that appropriately trained, supervised and supported CHWs along with engaged communities are needed to achieve effectiveness, and these conditions appear to have been met in the projects included in our review.

This review demonstrates that four major strategies for delivering community–based primary health care interventions are effective and commonly used in projects that have improved child health. These strategies are (1) house–to–house visitation by CHWs; (2) community case management of childhood illness, (3) use of participatory women’s groups; and (4) outreach services provided in the community by mobile teams based at peripheral health centers. CHWs visit households to educate child caregivers about prevention and manage common illnesses. Through following well–developed protocols, CHWs link community members to their nearest health facility for management of serious illness or follow up. These strategies are discussed in detail elsewhere in this series from the perspective of CBPHC strategies for improving maternal, neonatal as well as child health [18].

Many assessments included in our review support the importance of community engagement. A systematic review of child survival programs has found that programs working collaboratively with the community can lead to cost–effective transformation and lasting behavior change that produces improved health outcomes [19]. As a result of such engagements, the knowledge that community members have about what works locally is more likely to be shared with health program staff because they have a shared responsibility for program planning, implementation and evaluation. Without being a stakeholder, community members may see programs as imposed from the outside and not responsive to their needs. Without community engagement, programs may not produce the best outcomes that might otherwise be achieved through strong community engagement.

While we have made every effort to include all relevant studies that meet our criteria, some important studies have escaped our screening process. One such study concerns the use of pre–referral rectal artesunate [20]. In a randomized controlled trial in Bangladesh, Ghana and Tanzania, patients aged 6 to 72 months with suspected severe malaria who could not be treated orally were allocated randomly to receive a single rectal dose of artesunate (n = 8954) or placebo (n = 8872) before referral to a clinic where antimalarial injections could be given. In patients who had not reached a clinic within 6 hours, half of whom had not reached a clinic within 15 hours, pre–referral artesunate significantly reduced death or permanent disability by half (1.9% in the intervention group compared to 3.8% in the control group).

Several studies included in our review confirmed the effectiveness of Integrated Community Case Management (iCCM) (**Table 8**). However, several recent evaluations published since the end–point of publications selected for our review (31 December 2015) have found that iCCM, when implemented at scale, has not expanded coverage of key child survival interventions or reduced under–5 mortality, partly because of shortcomings related to training, supervision and drug stock outs [21] and low levels of care seeking [22,23]. Perhaps CHWs trained in iCCM are not able to make frequent home visits and therefore unable to give sufficient attention to educating mothers about warning signs for which they should seek care or to earn their confidence. Their broader job responsibilities beyond iCCM, including providing curative care for adults and family planning for women, as well as the large size of their catchment areas (sometimes more than 2 hours away from their health post) make frequent home visits virtually impossible.

As the Narangwal project demonstrated four decades ago, the provision of some curative care builds community trust in the CHWs providing it. It also facilitates referral to local health facilities as needed. However this trust is difficult to develop if the CHWs are not in regular contact with all households and if community members are not convinced that the CHWs are well trained and competent. One particularly important recent example of the effectiveness achieved by meeting these conditions occurred in Yirimadjo, Mali [S93]. The intervention included CHW active case finding, user fee removal, infrastructure development, community mobilization and prevention programming. After three years of the intervention, the hazard of under–5 mortality in the intervention area was one tenth that of baseline (HR 0.10 *P* < 0.0001), the prevalence of febrile illness of children younger than 5 years of age was significantly lower, from 38% at baseline to 23% at endline (*P* = 0.0009) and the percentage of children starting an effective antimalarial with 24 hours of symptom onset was nearly twice that reported at baseline (*P* = 0.0195).

The assessments from the Navrongo project in Ghana [S196–198] demonstrate that the best results were achieved when the community nurses worked in conjunction with trained community volunteers and community mobilization. The particular processes of community mobilization focused on working through the traditional community structure and engaging persons with a leadership role within the community. While the community–based nurses did have some impact on child mortality through their provision of prompt curative treatment, they did not have significant impact on contraceptive use or on child nutrition that require a high level of trust between community members and providers that can be achieved by community participation and door–to–door provision of support and health education [24]. A more recent evaluation of the extension of this program across Ghana indicates that an ongoing systematic approach with regular planning, monitoring and supervision of health workers, and close collaboration with community leaders needs to be followed to produce lasting results at scale [25].

For CBPHC to be most effective it must reach all households, including the poorest families, all mothers, those households far away, and those who are members of religious or ethnic minorities. In our review, the census–based, impact–oriented (CBIO) approach and Care Groups have demonstrated the importance of registering and visiting frequently all households with mothers and children, as more recent evidence has also demonstrated [26,27]. The Care Group approach has achieved excellent results at low cost [14] and is currently being implemented in many priority countries [13].

The following essential interventions for child health that can be provided at the level of the community and/or health post by CHWs have been identified [1]:

- Promote breastfeeding (including exclusive breastfeeding during the first six months of life) and appropriate complementary feeding beginning at 6 months of age
- Provide vitamin A and zinc supplementation
- Provide co–trimoxazole for HIV–positive children
- Educate families on safe disposal of children’s stools and hand washing
- Distribute and promote use of ITNs or IRs or both
- Detect and refer children with severe acute malnutrition
- Prevent, diagnose and treat of pneumonia, malaria and diarrheal diseases with early referral of those children with danger signs of serious disease.

The strong and consistent evidence that we have presented in this paper clearly demonstrates that all these Essential Interventions can be delivered at the community level with favorable population–level results for children.

The findings from this review also provide strong evidence that the four key strategies of delivering community–based interventions are effective approaches for achieving implementation effectiveness through CBPHC. These strategies are: (1) house–to–house visitation by CHWs; (2) community case management of childhood illness, (3) use of participatory women’s groups; and (4) outreach services provided in the community by mobile teams based at health centers. We have also presented evidence that community participation and mobilization make a strong contribution to intervention effectiveness.

### Study limitations

Some of the studies included in our review lacked sufficient information about the assessment methodology, about the role of community members and other implementation strategies, as well as about the outcomes themselves. This sometimes made it difficult to assess the strength of the evidence and to draw firm conclusions. We worked to mitigate this limitation by, in some cases, following up with the authors of these assessments.

Due to space limitations not all 489 assessments of the effectiveness of CBPHC in improving child health could be cited in this analysis. However, the findings of the assessments not specifically cited here are consistent with and supportive of those that were cited.

As is well–known, project failures and serious challenges encountered in program implementation are rarely described in open–access documents or in the scientific literature. This means that a serious publication bias is present and should be recognized. Nonetheless, publication bias does not negate the value of the numerous assessments that have been included in our review that demonstrate effectiveness of CBPHC in improving child health. The consistency of findings across many assessments in relationship to most interventions is such that we are convinced that the general findings with respect to each specific intervention are valid.

We acknowledge that there may be some assessments that qualified for our review that were not picked up by our screening procedures. However, we do not think that the inclusion of any articles we might have missed would alter the overall findings from our review. In addition, we are aware that there are important findings in papers published after December 2015 that did not fit the timeline of our review, but we have highlighted them in the discussion.

Our review has identified several areas of further study that are needed to address gaps in current knowledge to improve the implementation of child health programs at the community level. These areas are:

- Effectiveness studies of the implementation of community based interventions at scale in large populations in routine settings for 5 or more years;
- Effectiveness studies on how best to involve communities in the monitoring, implementation and evaluation of these settings.

As can be readily seen from the tables in this paper there is a clear lack of assessments of studies of interventions in large populations at scale. In the final paper of this series [28] the Expert Panel highlights the need for more evidence from programs delivered at scale. Similarly, while we have provided evidence that many interventions can be implemented successfully at the community level, the actual results produced in the field depend on how well community members “own” and therefore use the interventions provided in a sustainable manner. How to best do this needs further investigation.

Given the heterogeneity of (1) the types of interventions implemented, (2) the manner in which they were implemented, and (3) the outcome measures used to assess outcomes, it is not possible to make any definitive statements about the strength of the evidence or the magnitude of effect for any specific intervention or any specific approach to implementation, or how any given intervention or implementation approach compares with another in terms of effectiveness. Moreover, addressing the important issue of how to most effectively integrate interventions into a balanced package of services so that the demands for implementation of one intervention do not override the requirements for implementation of another intervention is beyond the scope of this paper, as is the important issue of how to strengthen health systems more broadly to better support the implementation of effective CBPHC interventions for improving child health.

Nonetheless, consistent with the purpose of our overall review of the effectiveness of CBPHC in improving MNCH, our overall findings strongly support the conclusion that (1) CBPHC can in fact be effectively implemented at the community level to improve child health and (2) robust community–based delivery systems are needed in order for the evidence–based interventions currently known and those that will be developed can reach their full potential.

## CONCLUSIONS

We have presented the evidence of effectiveness of a broad range of community–based interventions for improving the health of children 1–59 months of age. Health systems that are capable of achieving universal coverage of these interventions in high–mortality settings are clearly needed. Achieving this capability will require strong support for the health system as well as a strong commitment to a well–trained and well–supported CHW cadre in sufficient numbers. Understanding the conditions that need to be met in order for these interventions to be effective at scale in routine settings in priority countries and ensuring that these conditions are met will be the major challenge in the decade to come.
