# Supplementary material for: Comprehensive review of the evidence regarding the effectiveness of community–based primary health care in improving maternal, neonatal and child health: 4. child health findings
Source: J Glob Health. 2017 Jun 28;7(1):010904. doi: 10.7189/jogh.07.010904 (PMC5491948; doi:10.7189/jogh.07.010904)
Supplement: Online Supplementary Document [file jogh-07-010904-s001.pdf]

Online Supplementary Document

Freeman et al. Comprehensive review of the evidence regarding the effectiveness of community–based primary health care in improving maternal, neonatal and child health: 4. child health findings.

J Glob Health 2017;7:010904

Appendix S1

Bibliography of Assessments Involving Children 1-59 Months of Age

Aaby, P., Roth, A., Ravn, H., Napirna, B. M., Rodrigues, A., Lisse, I. M., . . . Benn, C. S. (2011). Randomized trial of BCG vaccination at birth to low-birth-weight children: beneficial nonspecific effects in the neonatal period? J Infect Dis, 204(2), 245-252. doi:10.1093/infdis/jir240

Abbey, M., Bartholomew, L. K., Pappoe, M., & van den Borne, B. (2015). Treating fever in children under 5 years of age: caregiver perceptions of community health worker services in Dangme West district, Ghana. Int Health, 7(6), 455-463. doi:10.1093/inthealth/ihv027

AbdelRahman, S. H., Albashir, I. M., Hussein, S. A., Ahmed, M. E., Alfadil, S. M., & Mohamed, N. (2007). BDN programmes and the effect of medical students' interventions to promote child health in Sudan. East Mediterr Health J, 13(6), 1319-1329.

Abdulla, S., Schellenberg, J. A., Nathan, R., Mukasa, O., Marchant, T., Smith, T., . . . Lengeler, C. (2001). Impact on malaria morbidity of a programme supplying insecticide treated nets in children aged under 2 years in Tanzania: community cross sectional study. BMJ, 322(7281), 270-273. doi:http://dx.doi.org/10.1136/bmj.322.7281.270

Adu-Afarwuah, S., Lartey, A., Brown, K. H., Zlotkin, S., Briend, A., & Dewey, K. G. (2007). Randomized comparison of 3 types of micronutrient supplements for home fortification of complementary foods in Ghana: effects on growth and motor development. Am J Clin Nutr, 86(2), 412-420.

Adventist Development Relief Agency/Cambodia. (2006). Child survival XVII - Final evaluation.

Adventist Development Relief Agency/Guinea. (2005). Child survival XVI: Final Evaluation Siguiri Prefecture

Adventist Development Relief Agency/Nicaragua. (2006). Final Evaluation: Child Survival XVII.

Afari, E. A., Nkrumah, F. K., Nakana, T., Sakatoku, H., Hori, H., & Binka, F. (1995). Impact of primary health care on child morbidiy and mortality. Central African Journal of Medicine, 41(5), 148-153.

Afsana, K., Haque, M. R., Sobhan, S., & Shahin, S. A. (2014). BRAC's experience in scaling-up MNP in Bangladesh. Asia Pac J Clin Nutr, 23(3), 377-384. doi:10.6133/apjcn.2014.23.3.22

Agarwal, D. K., Bhatia, B. D., & Agarwal, K. N. (1993). Simple approach to acute respiratory infection in rural under five children. Indian Pediatr, 30(5), 629-635.

Ahmed, N. U., Zeitlin, M. F., Beiser, A. S., Super, C. M., & Gershoff, S. N. (1993). A longitudinal study of the impact of behavioural change intervention on cleanliness, diarrhoeal morbidity and growth of children in rural Bangladesh. Soc Sci Med, 37(2), 159-171. doi:http://dx.doi.org/10.1016/0277-9536(93)90452-a

Ahmed, S., Kim, M. H., Dave, A. C., Sabelli, R., Kanjelo, K., Preidis, G. A., . . . Abrams, E. J. (2015). Improved identification and enrolment into care of HIV-exposed and -infected infants and children following a community health worker intervention in Lilongwe, Malawi. J Int AIDS Soc, 18(1), 19305. doi:10.7448/IAS.18.1.19305

Ajayi, I. O., Browne, E. N., Garshong, B., Bateganya, F., Yusuf, B., Agyei-Baffour, P., . . . Pagnoni, F. (2008). Feasibility and acceptability of artemisinin-based combination therapy for the home management of malaria in four African sites. Malar J, 7, 6. doi:10.1186/1475-2875-7-6

Alam, D. S., Yunus, M., El Arifeen, S., Chowdury, H. R., Larson, C. P., Sack, D. A., . . . Black, R. E. (2011). Zinc treatment for 5 or 10 days is equally efficacious in preventing diarrhea in the subsequent 3 months among Bangladeshi children. J Nutr, 141(2), 312-315. doi:10.3945/jn.110.120857

Alderman, H. (2007). Improving nutrition through community growth promotion: Longitudinal study of the nutrition and early child development program in Uganda. World Development, 35(8), 1376-1389. doi:http://dx.doi.org/10.1016/j.worlddev.2007.04.003

Alderman, H., Ndiaye, B., Linnemayr, S., Ka, A., Rokx, C., Dieng, K., & Mulder-Sibanda, M. (2008). Effectiveness of a community-based intervention to improve nutrition in young children in Senegal: a difference in difference analysis. Public Health Nutr, 12(5), 667-673. doi:10.1017/S1368980008002619

Ali, M., Emch, M., Tofail, F., & Baqui, A. H. (2001). Implications of health care provision on acute lower respiratory infection mortality in Bangladeshi children. Soc Sci Med, 52(2), 267-277. doi:http://dx.doi.org/10.1016/s0277-9536(00)00120-9

Alisjahbana, A., Williams, C., Dharmayanti, R., Hermawan, D., Kwast, B. E., & Koblinsky, M. (1995). An integrated village maternity service to improve referral patterns in a rural area in West-Java. Int J Gynaecol Obstet, 48 Suppl, S83-94. doi:http://dx.doi.org/10.1016/0020-7292(95)02323-5

Allen, S. J., Snow, R. W., Menon, A., & Greenwood, B. M. (1990). Compliance with malaria chemoprophylaxis over a five-year period among children in a rural area of The Gambia. J Trop Med Hyg, 93(5), 313-322.

Alonso, P. L., Lindsay, S. W., Armstrong, J. R., Conteh, M., Hill, A. G., David, P. H., . . . et al. (1991). The effect of insecticide-treated bed nets on mortality of Gambian children. Lancet, 337(8756), 1499-1502. doi:http://dx.doi.org/10.1016/0140-6736(91)93194-e

Ameha, A., Karim, A. M., Erbo, A., Ashenafi, A., Hailu, M., Hailu, B., . . . Betemariam, W. (2014). Effectiveness of supportive supervision on the consistency of integrated community cases management skills of the health extension workers in 113 districts of Ethiopia. Ethiop Med J, 52 Suppl 3, 65-71.

Amouzou, A., Morris, S., Moulton, L. H., & Mukanga, D. (2014). Assessing the impact of integrated community case management (iCCM) programs on child mortality: Review of early results and lessons learned in sub-Saharan Africa. J Glob Health, 4(2), 020411. doi:10.7189/jogh.04.020411

Aquino, R., de Oliveira, N. F., & Barreto, M. L. (2009). Impact of the family health program on infant mortality in Brazilian municipalities. Am J Public Health, 99(1), 87-93. doi:10.2105/AJPH.2007.127480

Arifeen, S. E., Blum, L. S., Hoque, D. M., Chowdhury, E. K., Khan, R., Black, R. E., . . . Bryce, J. (2004). Integrated Management of Childhood Illness (IMCI) in Bangladesh: early findings from a cluster-randomised study. Lancet, 364(9445), 1595-1602. doi:10.1016/S0140-6736(04)17312-1

Arifeen, S. E., Hoque, D. M., Akter, T., Rahman, M., Hoque, M. E., Begum, K., . . . Black, R. E. (2009). Effect of the Integrated Management of Childhood Illness strategy on childhood mortality and nutrition in a rural area in Bangladesh: a cluster randomised trial. Lancet, 374(9687), 393-403. doi:10.1016/S0140-6736(09)60828-X

Arole, M., & Arole, R. (1994). Jamkhed: A comprehensive rural health project. Hong Kong: The Macmillan Press.

Asling-Monemi, K., Tabassum Naved, R., & Persson, L. A. (2008). Violence against women and the risk of under-five mortality: analysis of community-based data from rural Bangladesh. Acta Paediatr, 97(2), 226-232. doi:10.1111/j.1651-2227.2007.00597.x

Aubel, J., Toure, I., & Diagne, M. (2004). Senegalese grandmothers promote improved maternal and child nutrition practices: the guardians of tradition are not averse to change. Soc Sci Med, 59(5), 945-959. doi:10.1016/j.socscimed.2003.11.044

Awasthi, S., Pande, V. K., & Fletcher, R. H. (2000). Effectiveness and cost-effectiveness of albendazole in improving nutritional status of pre-school children in urban slums. Indian Pediatr, 37(1), 19-29.

Awor, P., Wamani, H., Tylleskar, T., & Peterson, S. (2015). Drug seller adherence to clinical protocols with integrated management of malaria, pneumonia and diarrhoea at drug shops in Uganda. Malar J, 14, 277. doi:10.1186/s12936-015-0798-9

Ayuku, D., Embleton, L., Koech, J., Atwoli, L., Hu, L., Ayaya, S., . . . Braitstein, P. (2014). The government of Kenya cash transfer for orphaned and vulnerable children: cross-sectional comparison of household and individual characteristics of those with and without. BMC Int Health Hum Rights, 14, 25. doi:10.1186/1472-698X-14-25

Azad, K., Barnett, S., Banerjee, B., Shaha, S., Khan, K., Rego, A. R., . . . Costello, A. (2010). Effect of scaling up women's groups on birth outcomes in three rural districts in Bangladesh: a cluster-randomised controlled trial. Lancet, 375(9721), 1193-1202. doi:10.1016/S0140-6736(10)60142-0

Aziz, K. M., Hoque, B. A., Hasan, K. Z., Patwary, M. Y., Huttly, S. R., Rahaman, M. M., & Feachem, R. G. (1990). Reduction in diarrhoeal diseases in children in rural Bangladesh by environmental and behavioural modifications. Trans R Soc Trop Med Hyg, 84(3), 433-438. doi:http://dx.doi.org/10.1016/0035-9203(90)90353-g

Babalola, S., Sakolsky, N., Vondrasek, C., Mounlom, D., Brown, J., & Tchupo, J. P. (2001). The impact of a community mobilization project on health-related knowledge and practices in Cameroon. J Community Health, 26(6), 459-477.

Bachmann, M. O. (2009). Cost effectiveness of community-based therapeutic care for children with severe acute malnutrition in Zambia: decision tree model. Cost Eff Resour Alloc, 7, 2. doi:10.1186/1478-7547-7-2

Baek, C., Mathambo, V., Mkhize, S., Friedman, I., Apicella, L., & Rutenberg, N. (2007). Key findings from an evaluation of the mother2mothers programs in KwaZulu-Natal, South Africa.

Baker, E. J., Sanei, L. C., & Franklin, N. (2006). Early initiation of and exclusive breastfeeding in large-scale community-based programmes in Bolivia and Madagascar. J Health Popul Nutr, 24(4), 530-539.

Bang, A. T., Bang, R. A., & Sontakke, P. G. (1994). Management of childhood pneumonia by traditional birth attendants. The SEARCH Team. Bull World Health Organ, 72(6), 897-905.

Bang, A. T., Bang, R. A., Morankar, V. P., Sontakke, P. G., & Solanki, J. M. (1993). Pneumonia in neonates: can it be managed in the community? (Vol. 68).

Bang, A. T., Bang, R. A., Tale, O., Sontakke, P., Solanki, J., Wargantiwar, R., & Kelzarkar, P. (1990). Reduction in pneumonia mortality and total childhood mortality by means of community-based intervention trial in Gadchiroli, India. Lancet, 336(8709), 201-206. doi:http://dx.doi.org/10.1016/0140-6736(90)91733-q

Baqui, A. H., Ahmed, S., El Arifeen, S., Darmstadt, G. L., Rosecrans, A. M., Mannan, I., . . . Projahnmo 1 Study, G. (2009). Effect of timing of first postnatal care home visit on neonatal mortality in Bangladesh: a observational cohort study. BMJ, 339, b2826. doi:10.1136/bmj.b2826

Baqui, A. H., Black, R. E., El Arifeen, S., Yunus, M., Chakraborty, J., Ahmed, S., & Vaughan, J. P. (2002). Effect of zinc supplementation started during diarrhoea on morbidity and mortality in Bangladeshi children: community randomised trial. BMJ, 325(7372), 1059. doi:http://dx.doi.org/10.1136/bmj.325.7372.1059

Baqui, A. H., El-Arifeen, S., Darmstadt, G. L., Ahmed, S., Williams, E. K., Seraji, H. R., . . . Projahnmo Study, G. (2008). Effect of community-based newborn-care intervention package implemented through two service-delivery strategies in Sylhet district, Bangladesh: a cluster-randomised controlled trial. Lancet, 371(9628), 1936-1944. doi:10.1016/S0140-6736(08)60835-1

Baqui, A. H., Zaman, K., Persson, L. A., El Arifeen, S., Yunus, M., Begum, N., & Black, R. E. (2003). Simultaneous weekly supplementation of iron and zinc is associated with lower morbidity due to diarrhea and acute lower respiratory infection in Bangladeshi infants. J Nutr, 133(12), 4150-4157.

Baqui, A., Williams, E. K., Rosecrans, A. M., Agrawal, P. K., Ahmed, S., Darmstadt, G. L., . . . Santosham, M. (2008). Impact of an integrated nutrition and health programme on neonatal mortality in rural northern India. Bull World Health Organ, 86(10), 796-804, A. doi:http://dx.doi.org/10.2471/blt.07.042226

Bari, A., Sadruddin, S., Khan, A., Khan, I., Khan, A., Lehri, I. A., . . . Qazi, S. A. (2011). Community case management of severe pneumonia with oral amoxicillin in children aged 2-59 months in Haripur

district, Pakistan: a cluster randomised trial. Lancet, 378(9805), 1796-1803. doi:10.1016/S0140-6736(11)61140-9
Bari, S., Mannan, I., Rahman, M. A., Darmstadt, G. L., Serajil, M. H., Baqui, A. H., . . . Bangladesh Projahnmo, I. I. S. G. (2006). Trends in use of referral hospital services for care of sick newborns in a community-based intervention in Tangail District, Bangladesh. J Health Popul Nutr, 24(4), 519-529.
Barreto, M. L., Genser, B., Strina, A., Teixeira, M. G., Assis, A. M., Rego, R. F., . . . Cairncross, S. (2007). Effect of city-wide sanitation programme on reduction in rate of childhood diarrhoea in northeast Brazil: assessment by two cohort studies. Lancet, 370(9599), 1622-1628. doi:10.1016/S0140-6736(07)61638-9
Bashour, H. N., Kharouf, M. H., Abdulsalam, A. A., El Asmar, K., Tabbaa, M. A., & Cheikha, S. A. (2008). Effect of postnatal home visits on maternal/infant outcomes in Syria: a randomized controlled trial. Public Health Nurs, 25(2), 115-125. doi:10.1111/j.1525-1446.2008.00688.x
BASICS II/Madagascar (2004). Improving family health using an integrated community-based approach: Madagarcar Case Study.
BASICS II/Senegal (2004). CHWs in Senegal can appropriately treat pneumonia with cotrimoxazole.
BASICS II/Uganda (2003). Increasing immunization coverage in Uganda: The community problem solving and strategy development approach.
Basics/Rwanda. (2007). External evaluation of the pilot phase of the home-based management of malaria program in Rwanda: Final report.
Bawah, A. A., Philip, J. F., Asuming, P., Walega, P., Wak, G., Schmitt, M., & Oduro, A. (2012). Can Community Health Services Offset the Effect of Poverty and Low Maternal Educational Attainment on Childhood Mortality? Evidence from the Navrongo Experiment in Northern Ghana. Paper presented at the Second Global Symposium on Health Systems Research, Beijing, China.
Bawah, A. A., Philips, J. F., Adjuik, M., Vaughan-Smith, M., Macleod, B., & Binka, F. N. (2006). The impact of immunization on the association between poverty and child survival: evidence from Kassena-Nankana district of northern Ghana. Population Council Working Papers 2/8. New York, NY
Becker, S. R., Diop, F., & Thornton, J. N. (1993). Infant and child mortality in two counties of Liberia: results of a survey in 1988 and trends since 1984. Int J Epidemiol, 22 Suppl 1, S56-63. doi:http://dx.doi.org/10.1093/ije/22.supplement_1.s56
Benn, C. S., Diness, B. R., Roth, A., Nante, E., Fisker, A. B., Lisse, I. M., . . . Aaby, P. (2008). Effect of 50,000 IU vitamin A given with BCG vaccine on mortality in infants in Guinea-Bissau: randomised placebo controlled trial. BMJ, 336(7658), 1416-1420. doi:10.1136/bmj.39542.509444.AE
Bentley, C. (1989). Primary health care in northwestern Somalia: a case study. Soc Sci Med, 28(10), 1019-1030. doi:http://dx.doi.org/10.1016/0277-9536(89)90384-5
Berggren, G. (1997). Nutritional education and rehabilitation program: A Save the Children project. In O. Wollinka et al (Eds.), Vietnam Hearth Nutrition Model: Applications in Haiti, Vietnam, and Bangladesh (pp. 43-46). Arlington, VA: World Relief
Berggren, G. G., Berggren, W., Verly, A., Garnier, N., Peterson, W., Ewbank, D., & Dieudonne, W. (1983). Traditional midwives, tetanus immunization, and infant mortality in rural Haiti. Trop Doct, 13(2), 79-87.
Berggren, G. G., Hebert, J. R., & Waternaux, C. M. (1985). Comparison of Haitian children in a nutrition intervention programme with children in the Haitian national nutrition survey. Bull World Health Organ, 63(6), 1141-1150.
Berggren, W. L. (1974). [Control of neonatal tetanus in the rural region of Haiti by using medical auxiliaries]. Bol Oficina Sanit Panam, 77(1), 6-12.
Berggren, W. L., Ewbank, D. C., & Berggren, G. G. (1981). Reduction of mortality in rural Haiti through a primary-health-care program. N Engl J Med, 304(22), 1324-1330. doi:10.1056/NEJM198105283042203

Bhandari, N., Bahl, R., Mazumdar, S., Martines, J., Black, R. E., Bhan, M. K., & Infant Feeding Study, G. (2003). Effect of community-based promotion of exclusive breastfeeding on diarrhoeal illness and growth: a cluster randomised controlled trial. Lancet, 361(9367), 1418-1423. doi:10.1016/S0140-6736(03)13134-0

Bhandari, N., Bahl, R., Nayyar, B., Khokhar, P., Rohde, J. E., & Bhan, M. K. (2001). Food supplementation with encouragement to feed it to infants from 4 to 12 months of age has a small impact on weight gain. J Nutr, 131(7), 1946-1951.

Bhandari, N., Bahl, R., Taneja, S., Strand, T., Molbak, K., Ulvik, R. J., . . . Bhan, M. K. (2002). Effect of routine zinc supplementation on pneumonia in children aged 6 months to 3 years: randomised controlled trial in an urban slum. BMJ, 324(7350), 1358. doi:http://dx.doi.org/10.1136/bmj.324.7350.1358

Bhandari, N., Bahl, R., Taneja, S., Strand, T., Molbak, K., Ulvik, R. J., . . . Bhan, M. K. (2002). Substantial reduction in severe diarrheal morbidity by daily zinc supplementation in young north Indian children. Pediatrics, 109(6), e86. doi:http://dx.doi.org/10.1007/bf02725586

Bhandari, N., Mazumder, S., Bahl, R., Martines, J., Black, R. E., Bhan, M. K., & Infant Feeding Study, G. (2004). An educational intervention to promote appropriate complementary feeding practices and physical growth in infants and young children in rural Haryana, India. J Nutr, 134(9), 2342-2348.

Bhandari, N., Mazumder, S., Bahl, R., Martines, J., Black, R. E., Bhan, M. K., & Infant Feeding Study, G. (2005). Use of multiple opportunities for improving feeding practices in under-twos within child health programmes. Health Policy Plan, 20(5), 328-336. doi:10.1093/heapol/czi039

Bhandari, N., Mazumder, S., Taneja, S., Dube, B., Agarwal, R. C., Mahalanabis, D., . . . Bhan, M. K. (2008). Effectiveness of zinc supplementation plus oral rehydration salts compared with oral rehydration salts alone as a treatment for acute diarrhea in a primary care setting: a cluster randomized trial. Pediatrics, 121(5), e1279-1285. doi:10.1542/peds.2007-1939

Bhuiya, A., & Chowdhury, M. (2002). Beneficial effects of a woman-focused development programme on child survival: evidence from rural Bangladesh. Soc Sci Med, 55(9), 1553-1560. doi:http://dx.doi.org/10.1016/s0277-9536(01)00287-8

Bhutta, Z. A., Memon, Z. A., Soofi, S., Salat, M. S., Cousens, S., & Martines, J. (2008). Implementing community-based perinatal care: results from a pilot study in rural Pakistan. Bull World Health Organ, 86(6), 452-459. doi:http://dx.doi.org/10.2471/blt.07.045849

Bhutta, Z. A., Rizvi, A., Raza, F., Hotwani, S., Zaidi, S., Moazzam Hossain, S., . . . Bhutta, S. (2009). A comparative evaluation of multiple micronutrient and iron-folic acid supplementation during pregnancy in Pakistan: impact on pregnancy outcomes. Food Nutr Bull, 30(4 Suppl), S496-505. doi:http://dx.doi.org/10.1177/15648265090304s404

Bilous, J., Maher, C., Tangermann, R. H., Aylward, R. B., Schnur, A., Sanders, R., . . . Omi, S. (1997). The experience of countries in the Western Pacific Region in conducting national immunization days for poliomyelitis eradication. J Infect Dis, 175 Suppl 1, S194-197. doi:http://dx.doi.org/10.1093/infdis/175.supplement_1.s194

Binka, F. N., Bawah, A. A., Phillips, J. F., Hodgson, A., Adjuik, M., & MacLeod, B. (2007). Rapid achievement of the child survival millennium development goal: evidence from the Navrongo experiment in Northern Ghana. Trop Med Int Health, 12(5), 578-583. doi:10.1111/j.1365-3156.2007.01826.x

Binka, F. N., Kubaje, A., Adjuik, M., Williams, L. A., Lengeler, C., Maude, G. H., . . . Smith, P. G. (1996). Impact of permethrin impregnated bednets on child mortality in Kassena-Nankana district, Ghana: a randomized controlled trial. Trop Med Int Health, 1(2), 147-154. doi:http://dx.doi.org/10.1111/j.1365-3156.1996.tb00020.x

Bishai, D., Kumar, K. C. S., Waters, H., Koenig, M., Katz, J., Khatry, S. K., & West, K. P., Jr. (2005). The impact of vitamin A supplementation on mortality inequalities among children in Nepal. Health Policy Plan, 20(1), 60-66. doi:10.1093/heapol/czi007

Bisimwa, G., Mambo, T., Mitangala, P., Schirvel, C., Porignon, D., Dramaix, M., & Donnen, P. (2009). Nutritional monitoring of preschool-age children by community volunteers during armed conflict in the Democratic Republic of the Congo. Food Nutr Bull, 30(2), 120-127. doi:http://dx.doi.org/10.1177/156482650903000203

Bohler, E. (1994). Has primary health care reduced infant mortality in east Bhutan? The effects of primary health care and birth spacing on infant and child mortality patterns in east Bhutan. J Trop Pediatr, 40(5), 256-260. doi:http://dx.doi.org/10.1093/tropej/40.5.256

Bojang, K. A., Akor, F., Conteh, L., Webb, E., Bittaye, O., Conway, D. J., . . . Greenwood, B. (2011). Two strategies for the delivery of IPTc in an area of seasonal malaria transmission in the Gambia: a randomised controlled trial. PLoS Med, 8(2), e1000409. doi:10.1371/journal.pmed.1000409

Bosu, W. K., Ahelegbe, D., Edum-Fotwe, E., Bainson, K. A., & Turkson, P. K. (1997). Factors influencing attendance to immunization sessions for children in a rural district of Ghana. Acta Trop, 68(3), 259-267. doi:http://dx.doi.org/10.1016/s0001-706x(97)00094-6

Bowen, A., Agboatwalla, M., Luby, S., Tobery, T., Ayers, T., & Hoekstra, R. M. (2012). Association between intensive handwashing promotion and child development in Karachi, Pakistan: a cluster randomized controlled trial. Arch Pediatr Adolesc Med, 166(11), 1037-1044. doi:10.1001/archpediatrics.2012.1181

Brenner, J. L., Kabakyenga, J., Kyomuhangi, T., Wotton, K. A., Pim, C., Ntaro, M., . . . Singhal, N. (2011). Can volunteer community health workers decrease child morbidity and mortality in southwestern Uganda? An impact evaluation. PLoS One, 6(12), e27997. doi:10.1371/journal.pone.0027997

Brooks, W. A., Santosham, M., Naheed, A., Goswami, D., Wahed, M. A., Diener-West, M., . . . Black, R. E. (2005). Effect of weekly zinc supplements on incidence of pneumonia and diarrhoea in children younger than 2 years in an urban, low-income population in Bangladesh: randomised controlled trial. Lancet, 366(9490), 999-1004. doi:10.1016/S0140-6736(05)67109-7

Brugha, R. F., & Kevany, J. P. (1996). Maximizing immunization coverage through home visits: a controlled trial in an urban area of Ghana. Bull World Health Organ, 74(5), 517-524.

Bryce, J., Gilroy, K., Jones, G., Hazel, E., Black, R. E., & Victora, C. G. (2008). The Retrospective Evaluation of ACSD: Cross-site analyses and conclusions. Retrieved from

Burkhalter, B. R., & Northrup, R. S. (1997). Hearth Program at the Hôpital Albert Schweitzer in Haiti Hearth Nutrition Model: Applications in Haiti, Vietnam, and Bangladesh. USAID: Basic Support for Institutionalizing Child Survival (BASICS) Project. Arlington, VA.

Callaghan-Koru, J. A., Nonyane, B. A., Guenther, T., Sitrin, D., Ligowe, R., Chimbalanga, E., . . . Baqui, A. H. (2013). Contribution of community-based newborn health promotion to reducing inequities in healthy newborn care practices and knowledge: evidence of improvement from a three-district pilot program in Malawi. BMC Public Health, 13, 1052. doi:10.1186/1471-2458-13-1052

Canner, J., Brown, J., Barrows, J., & Foundation/Malawi, I. E. (1994, October 2-7, 1994). Reduction in Diarrheal mortality due to Vitamin A supplementation in Chikwawa district, Malawi. Paper presented at the Community Impact of PVO Child Survival Efforts: 1985-1994, Bangalore, Karnataka, India.

Care /Nicaragua (2008). Child Survival Project (CSP) XVIII: Phase two - Final Evaluation.

Care/Ethiopia. (2007). Farta child survival project, Amhara National state, South Gondar administrative zone, Farta Woreda, Ethiopia.

Care/Kenya. (1999). Community initiatives for child survival Siaya: Final evaluation.

Care/Mozambique. (2006). Child survial project: Final evaluation.

Carlo, W. A., Goudar, S. S., Pasha, O., Chomba, E., Wallander, J. L., Biasini, F. J., . . . Children's Health Research, I. (2013). Randomized trial of early developmental intervention on outcomes in children after birth asphyxia in developing countries. J Pediatr, 162(4), 705-712 e703. doi:10.1016/j.jpeds.2012.09.052

Catholic Relief Services/Nicaragua. (2012). Child Survival and Health Project: Final Evaluation.

CB-MNC Nepal (2007). Community-based maternal and neonatal care program (CB-MNC): Summative report on program activities and results in Banka, Jhapa and Kanchanpur districts from September 2005 - September 2007.

Cesar, J. A., Cavaleti, M. A., Holthausen, R. S., & de Lima, L. G. (2002). [Changes in child health indicators in a municipality with community health workers: the case of Itapirapua Paulista, Vale do Ribeira, Sao Paulo State, Brazil]. Cad Saude Publica, 18(6), 1647-1654. doi:http://dx.doi.org/10.1590/s0102-311x2002000600019

Cesar, J. A., Goncalves, T. S., Neumann, N. A., Oliveira Filho, J. A., & Diziekaniak, A. C. (2005). [Child health in poor areas of North and Northeast Brazil: a comparison of areas covered by the Children's Mission and control areas]. Cad Saude Publica, 21(6), 1845-1855. doi:/S0102-311X2005000600034

Cesar, J. A., Mendoza-Sassi, R. A., Ulmi, E. F., Dall'Agnol, M. M., & Neumann, N. A. (2008). [Effects of different home visit strategies on prenatal care in Southern Brazil]. Cad Saude Publica, 24(11), 2614-2622. doi:http://dx.doi.org/10.1590/s0102-311x200800110001

Chahnazarian, A., Ewbank, D. C., Makani, B., & Ekouevi, K. (1993). Impact of selective primary care on childhood mortality in a rural health zone of Zaire. Int J Epidemiol, 22 Suppl 1, S32-41. doi:http://dx.doi.org/10.1093/ije/22.supplement_1.s32

Chaiken, M. S., Deconinck, H., & Degefie, T. (2006). The promise of a community-based approach to managing severe malnutrition: A case study from Ethiopia. Food and Nutrition Bulletin, 27(2), 95-103. doi:http://dx.doi.org/10.1177/156482650602700201

Chen, L. C., Rahman, M., D'Souza, S., Chakraborty, J., Sardar, A. M., & Yunus, M. (1983). Mortality impact of an MCH-FP program in Matlab, Bangladesh. Stud Fam Plann, 14(8-9), 199-209. doi:http://dx.doi.org/10.2307/1966412

Chinbuah, M. A., Adjuik, M., Cobelens, F., Koram, K. A., Abbey, M., Gyapong, M., . . . Gyapong, J. O. (2013). Impact of treating young children with antimalarials with or without antibiotics on morbidity: a cluster-randomized controlled trial in Ghana. Int Health, 5(3), 228-235. doi:10.1093/inthealth/iht021

Chowdhury, A. M., Karim, F., Sarkar, S. K., Cash, R. A., & Bhuiya, A. (1997). The status of ORT (oral rehydration therapy) in Bangladesh: how widely is it used? Health Policy Plan, 12(1), 58-66. doi:http://dx.doi.org/10.1093/heapol/12.1.58

Christian, P., Stewart, C. P., LeClerq, S. C., Wu, L., Katz, J., West, K. P., Jr., & Khatry, S. K. (2009). Antenatal and postnatal iron supplementation and childhood mortality in rural Nepal: a prospective follow-up in a randomized, controlled community trial. Am J Epidemiol, 170(9), 1127-1136. doi:10.1093/aje/kwp253

Christian, P., West, K. P., Khatry, S. K., Leclerq, S. C., Pradhan, E. K., Katz, J., . . . Sommer, A. (2003). Effects of maternal micronutrient supplementation on fetal loss and infant mortality: a cluster-randomized trial in Nepal. Am J Clin Nutr, 78(6), 1194-1202.

Ciliberto, M. A., Sandige, H., Ndekha, M. J., Ashorn, P., Briend, A., Ciliberto, H. M., & Manary, M. J. (2005). Comparison of home-based therapy with ready-to-use therapeutic food with standard therapy in the treatment of malnourished Malawian children: a controlled, clinical effectiveness trial. Am J Clin Nutr, 81(4), 864-870.

Cisse, B., Cairns, M., Faye, E., O, N. D., Faye, B., Cames, C., . . . Milligan, P. (2009). Randomized trial of piperaquine with sulfadoxine-pyrimethamine or dihydroartemisinin for malaria intermittent preventive treatment in children. PLoS One, 4(9), e7164. doi:10.1371/journal.pone.0007164

Collins, S. (2007). Treating severe acute malnutrition seriously. Arch Dis Child, 92(5), 453-461. doi:10.1136/adc.2006.098327

Collins, S., & Sadler, K. (2002). Outpatient care for severely malnourished children in emergency relief programmes: a retrospective cohort study. Lancet, 360(9348), 1824-1830. doi:10.1016/S0140-6736(02)11770-3

Concern Worldwide/Bangladesh (2008). The end of magical thinking: Sustainability evaluation three years after the end of the Saidpur and Parbatipur urban health project.

Concern Worldwide/Rwanda (2011). Final evaluation of the Kabeho Mwana expanded impact child survival program.

Conroy, R. M., Elmore-Meegan, M., Joyce, T., McGuigan, K. G., & Barnes, J. (1996). Solar disinfection of drinking water and diarrhoea in Maasai children: a controlled field trial. Lancet, 348(9043), 1695-1697. doi:10.1016/S0140-6736(96)02309-4

Conroy, R. M., Meegan, M. E., Joyce, T., McGuigan, K., & Barnes, J. (1999). Solar disinfection of water reduces diarrhoeal disease: an update. Arch Dis Child, 81(4), 337-338. doi:http://dx.doi.org/10.1136/adc.81.4.337

Cumberland, P., Edwards, T., Hailu, G., Harding-Esch, E., Andreasen, A., Mabey, D., & Todd, J. (2008). The impact of community level treatment and preventative interventions on trachoma prevalence in rural Ethiopia. Int J Epidemiol, 37(3), 549-558. doi:10.1093/ije/dyn045

Cunningham, N. (1978). The under fives clinic--what difference does it make? J Trop Pediatr Environ Child Health, 24(6), 239-334. doi:http://dx.doi.org/10.1093/tropej/24.6.237

Curamericas Global/Liberia (2013). Final evaluation for Nehnwaa child survival project: Census-Based Impact-Oriented methodology for community-based primary health care in Nimba country, Liberia.

Curamericas/Bolivia. (2007). Providing child survival services to rural and peri-urban populations in Bolivia - Final Evaluation Report.

Curamericas/Guatemala (2007). Census-Based, Impact-Oriented Child Survival Project. October 1, 2002 - September 30, 2007: Final Evaluation Report

Curtale, F., Siwakoti, B., Lagrosa, C., LaRaja, M., & Guerra, R. (1995). Improving skills and utilization of community health volunteers in Nepal. Soc Sci Med, 40(8), 1117-1125. doi:http://dx.doi.org/10.1016/0277-9536(94)00172-p

D'Alessandro, U., Olaleye, B. O., McGuire, W., Langerock, P., Bennett, S., Aikins, M. K., . . . Greenwood, B. M. (1995). Mortality and morbidity from malaria in Gambian children after introduction of an impregnated bednet programme. Lancet, 345(8948), 479-483. doi:http://dx.doi.org/10.1016/s0140-6736(95)90582-0

Das, A., Friedman, J., Kandpal, E., Ramana, G. N., Gupta, R. K., Pradhan, M. M., & Govindaraj, R. (2014). Strengthening malaria service delivery through supportive supervision and community mobilization in an endemic Indian setting: an evaluation of nested delivery models. Malar J, 13, 482. doi:10.1186/1475-2875-13-482

Das, L. K., Jambulingam, P., & Sadanandane, C. (2008). Impact of community-based presumptive chloroquine treatment of fever cases on malaria morbidity and mortality in a tribal area in Orissa State, India. Malar J, 7, 75. doi:10.1186/1475-2875-7-75

Daulaire, N. M., Starbuck, E. S., Houston, R. M., Church, M. S., Stukel, T. A., & Pandey, M. R. (1992). Childhood mortality after a high dose of vitamin A in a high risk population. BMJ, 304(6821), 207-210. doi:http://dx.doi.org/10.1136/bmj.304.6821.207

Davies-Adetugbo, A. A. (1996). Promotion of breast feeding in the community: impact of health education programme in rural communities in Nigeria. J Diarrhoeal Dis Res, 14(1), 5-11.
Davis, T. P., Jr., Wetzel, C., Hernandez Avilan, E., de Mendoza Lopes, C., Chase, R. P., Winch, P. J., & Perry, H. B. (2013). Reducing child global undernutrition at scale in Sofala Province, Mozambique, using Care Group Volunteers to communicate health messages to mothers. Glob Health Sci Pract, 1(1), 35-51. doi:10.9745/GHSP-D-12-00045
Dawson, P., Pradhan, Y., Houston, R., Karki, S., Poudel, D., & Hodgins, S. (2008). From research to national expansion: 20 years' experience of community-based management of childhood pneumonia in Nepal. Bull World Health Organ, 86(5), 339-343. doi:http://dx.doi.org/10.2471/blt.07.047688
Dearden, K., Khan, N., & Children/Bangladesh, S. t. (1994, October 2-7, 1994). Do women's saving and credit programs affect fertility and health?: A case study from Bangladesh. Paper presented at the Community Impact of PVO Child Survival Efforts: 1985-1994, Bangalore, Karnataka, India.
Debpuur, C., Phillips, J. F., Jackson, E. F., Nazzar, A., Ngom, P., & Binka, F. N. (2002). The impact of the Navrongo Project on contraceptive knowledge and use, reproductive preferences, and fertility. Stud Fam Plann, 33(2), 141-164. doi:http://dx.doi.org/10.1111/j.1728-4465.2002.00141.x
Delacollette, C., Van der Stuyft, P., & Molima, K. (1996). Using community health workers for malaria control: experience in Zaire. Bull World Health Organ, 74(4), 423-430.
Deming, M. S., Gayibor, A., Murphy, K., Jones, T. S., & Karsa, T. (1989). Home treatment of febrile children with antimalarial drugs in Togo. Bull World Health Organ, 67(6), 695-700.
Deribew, A., Birhanu, Z., Sena, L., Dejene, T., Reda, A. A., Sudhakar, M., . . . Deribe, K. (2012). The effect of household heads training about the use of treated bed nets on the burden of malaria and anaemia in under-five children: a cluster randomized trial in Ethiopia. Malar J, 11, 8. doi:10.1186/1475-2875-11-8
Diallo, D. A., Cousens, S. N., Cuzin-Ouattara, N., Nebie, I., Ilboudo-Sanogo, E., & Esposito, F. (2004). Child mortality in a West African population protected with insecticide-treated curtains for a period of up to 6 years. Bull World Health Organ, 82(2), 85-91.
Diallo, I., Ndiaye, B., Pouye, A., Gaye, I. A., Sy, A., Sarr, R., & Tall-Dia, A. (1998). [Community nutrition strategy project: an innovation in community health]. Dakar Med, 43(2), 147-151.
Dibley, M. J., Sadjimin, T., Kjolhede, C. L., & Moulton, L. H. (1996). Vitamin A supplementation fails to reduce incidence of acute respiratory illness and diarrhea in preschool-age Indonesian children. J Nutr, 126(2), 434-442.
Dohn, A. L., Chavez, A., Dohn, M. N., Saturria, L., & Pimentel, C. (2004). Changes in health indicators related to health promotion and microcredit programs in the Dominican Republic. Rev Panam Salud Publica, 15(3), 185-193. doi:http://dx.doi.org/10.1590/s1020-49892004000300007
Doi, H., Kaneko, A., Panjaitan, W., & Ishii, A. (1989). Chemotherapeutic malaria control operation by single dose of Fansidar plus primaquine in North Sumatra, Indonesia. Southeast Asian J Trop Med Public Health, 20(3), 341-349.
Dongre, A. R., Deshmukh, P. R., & Garg, B. S. (2011). Community-led initiative for control of anemia among children 6 to 35 months of age and unmarried adolescent girls in rural Wardha, India. Food Nutr Bull, 32(4), 315-323. doi:http://dx.doi.org/10.1177/156482651103200402
Doocy, S., Teferra, S., Norell, D., & Burnham, G. (2005). Credit program outcomes: coping capacity and nutritional status in the food insecure context of Ethiopia. Soc Sci Med, 60(10), 2371-2382. doi:10.1016/j.socscimed.2004.10.025
du Preez, M., Conroy, R. M., Ligondo, S., Hennessy, J., Elmore-Meegan, M., Soita, A., & McGuigan, K. G. (2011). Randomized intervention study of solar disinfection of drinking water in the prevention of dysentery in Kenyan children aged under 5 years. Environ Sci Technol, 45(21), 9315-9323. doi:10.1021/es2018835
Dubowitz, T., Levinson, D., Peterman, J. N., Verma, G., Jacob, S., & Schultink, W. (2007). Intensifying efforts to reduce child malnutrition in India: an evaluation of the Dular program in Jharkhand, India. Food Nutr Bull, 28(3), 266-273. doi:http://dx.doi.org/10.1177/156482650702800302

Dubuisson, S. E., Ludzen, S., Zayan, A., Swedberg, E., & Haiti, S. t. C. (1994). Impact of sustainable behavior change on the nutritional status of children. Paper presented at the Community Impact of PVO Child Survival Efforts: 1985-1994, Bangalore, Karnataka, India.

Dutt, D., & Srinivasa, D. K. (1997). Impact of maternal and child health strategy on child survival in a rural community of Pondicherry. Indian Pediatr, 34(9), 785-792.

Edward, A., Ernst, P., Taylor, C., Becker, S., Mazive, E., & Perry, H. (2007). Examining the evidence of under-five mortality reduction in a community-based programme in Gaza, Mozambique. Trans R Soc Trop Med Hyg, 101(8), 814-822. doi:10.1016/j.trstmh.2007.02.025

Edwards, L., & Vision/India, W. (1994, Oct 2-7, 1994). Impact of PVO child survival interventions on the health of mother and child in 54 trival villages of India. Paper presented at the Community Impact of PVO Child Survival Efforts: 1985-1994, Bangalore, Karnataka, India.

Edwards, N. C., & Roelofs, S. M. (2006). Sustainability: the elusive dimension of international health projects. Can J Public Health, 97(1), 45-49.

Eklund, P., Imai, K., & Felloni, F. (2007). Women's organizations, maternal knowledge, and social capital to reduce prevalence of stunted children: evidence from rural Nepal. Journal of Development Studies, 43(3), 456-489. doi:http://dx.doi.org/10.1080/00220380701204406

Emond, A., Pollock, J., Da Costa, N., Maranhao, T., & Macedo, A. (2002). The effectiveness of community-based interventions to improve maternal and infant health in the Northeast of Brazil. Rev Panam Salud Publica, 12(2), 101-110. doi:http://dx.doi.org/10.1590/s1020-49892002000800005

Episcopal Relief & Development/Uganda. (2013). Ajula Pa Rwot (Child Survival) Project in Northern Uganda - Amuru and Gulu Districts.

Eriksen, J., Mujinja, P., Warsame, M., Nsimba, S., Kouyate, B., Gustafsson, L. L., . . . Tomson, G. (2010). Effectiveness of a community intervention on malaria in rural Tanzania - a randomised controlled trial. Afr Health Sci, 10(4), 332-340.

Escamilla, V., Wagner, B., Yunus, M., Streatfield, P. K., van Geen, A., & Emch, M. (2011). Effect of deep tube well use on childhood diarrhoea in Bangladesh. Bull World Health Organ, 89(7), 521-527. doi:10.2471/BLT.10.085530

Ewbank, D. C. (1993). Impact of health programmes on child mortality in Africa: evidence from Zaire and Liberia. Int J Epidemiol, 22 Suppl 1, S64-72. doi:http://dx.doi.org/10.1093/ije/22.supplement_1.s64

Fathima, F. N., Raju, M., Varadharajan, K. S., Krishnamurthy, A., Ananthkumar, S. R., & Mony, P. K. (2015). Assessment of 'accredited social health activists'-a national community health volunteer scheme in Karnataka State, India. J Health Popul Nutr, 33(1), 137-145.

Fatti, G., Shaikh, N., Eley, B., & Grimwood, A. (2014). Improved virological suppression in children on antiretroviral treatment receiving community-based adherence support: a multicentre cohort study from South Africa. AIDS Care, 26(4), 448-453. doi:10.1080/09540121.2013.855699

Fauveau, V., Stewart, M. K., Chakraborty, J., & Khan, S. A. (1992). Impact on mortality of a community-based programme to control acute lower respiratory tract infections. Bull World Health Organ, 70(1), 109-116.

Fauveau, V., Wojtyniak, B., Chakraborty, J., Sarder, A. M., & Briend, A. (1990). The effect of maternal and child health and family planning services on mortality: is prevention enough? BMJ, 301(6743), 103-107. doi:http://dx.doi.org/10.1136/bmj.301.6743.103

Fegan, G. W., Noor, A. M., Akhwale, W. S., Cousens, S., & Snow, R. W. (2007). Effect of expanded insecticide-treated bednet coverage on child survival in rural Kenya: a longitudinal study. Lancet, 370(9592), 1035-1039. doi:10.1016/S0140-6736(07)61477-9

Fernald, L. C., Gertler, P. J., & Neufeld, L. M. (2008). Role of cash in conditional cash transfer programmes for child health, growth, and development: an analysis of Mexico's Oportunidades. Lancet, 371(9615), 828-837. doi:10.1016/S0140-6736(08)60382-7

Fernald, L. C., Gertler, P. J., & Neufeld, L. M. (2009). 10-year effect of Oportunidades, Mexico's conditional cash transfer programme, on child growth, cognition, language, and behaviour: a longitudinal follow-up study. Lancet, 374(9706), 1997-2005. doi:10.1016/S0140-6736(09)61676-7
Fiedler, J. L. (2000). The Nepal National Vitamin A Program: prototype to emulate or donor enclave? Health Policy Plan, 15(2), 145-156. doi:http://dx.doi.org/10.1093/heapol/15.2.145
Fiedler, J. L., & Chuko, T. (2008). The cost of Child Health Days: a case study of Ethiopia's Enhanced Outreach Strategy (EOS). Health Policy Plan, 23(4), 222-233. doi:10.1093/heapol/czn015
Filoramo, L. (1997). Initiation of the Shishu Kabar Program in Southwestern Bangladesh Hearth Nutrition Model: Applications in Haiti, Vietnam, and Bangladesh. USAID: Basic Support for Institutionalizing Child Survival (BASICS) Project. Arlington, VA.
Food for the Hungry/Mozambique (2001). Food for the Hungry International/Mozambique Health/Nutrition Program: Final evaluation report.
Food for the Hungry/Mozambique (2008). Achieving equity, coverge, and impact through a care group network, Mozambique, Sofala Province.
Foster, S. O., Spiegel, R. A., Mokdad, A., Yeanon, S., Becker, S. R., Thornton, J. N., & Galakpai, M. K. (1993). Immunization, oral rehydration therapy and malaria chemotherapy among children under 5 in Bomi and Grand Cape Mount counties, Liberia, 1984 and 1988. Int J Epidemiol, 22 Suppl 1, S50-55. doi:http://dx.doi.org/10.1093/ije/22.supplement_1.s50
Fraser-Hurt, N., & Lyimo, E. O. (1998). Insecticide-treated nets and treatment service: a trial using public and private sector channels in rural United Republic of Tanzania. Bull World Health Organ, 76(6), 607-615.
Fraser-Hurt, N., Felger, I., Edoh, D., Steiger, S., Mashaka, M., Masanja, H., . . . Beck, H. P. (1999). Effect of insecticide-treated bed nets on haemoglobin values, prevalence and multiplicity of infection with Plasmodium falciparum in a randomized controlled trial in Tanzania. Trans R Soc Trop Med Hyg, 93 Suppl 1, 47-51. doi:http://dx.doi.org/10.1016/s0035-9203(99)90327-9
Future Generations/Afghanistan (2006). Community health worker training for women's empowerment in Afghanistan: Summary report.
Future Generations/Peru (2007). Internship Center Pilot Project The CLAS Las Moras-Huanuco: Mid-term evaluation.
Gabida, M., Chemhuru, M., Tshimanga, M., Gombe, N. T., Takundwa, L., & Bangure, D. (2015). Effect of distribution of educational material to mothers on duration and severity of diarrhoea and pneumonia, Midlands Province, Zimbabwe: a cluster randomized controlled trial. Int Breastfeed J, 10, 13. doi:10.1186/s13006-015-0037-6
Geissbuhler, Y., Kannady, K., Chaki, P. P., Emidi, B., Govella, N. J., Mayagaya, V., . . . Killeen, G. F. (2009). Microbial larvicide application by a large-scale, community-based program reduces malaria infection prevalence in urban Dar es Salaam, Tanzania. PLoS One, 4(3), e5107. doi:10.1371/journal.pone.0005107
Ghana VAST Study Team. (1993). Vitamin A supplementation in northern Ghana: effects on clinic attendances, hospital admissions, and child mortality. Ghana VAST Study Team. Lancet, 342(8862), 7-12. doi:http://dx.doi.org/10.1016/0140-6736(93)91879-q
Ghebreyesus, T. A., Alemayehu, T., Bosman, A., Witten, K. H., & Teklehaimanot, A. (1996). Community participation in malaria control in Tigray region Ethiopia. Acta Trop, 61(2), 145-156. doi:http://dx.doi.org/10.1016/0001-706x(95)00107-p
Ghimire, M., Pradhan, Y. V., & Maskey, M. K. (2010). Community-based interventions for diarrhoeal diseases and acute respiratory infections in Nepal. Bull World Health Organ, 88(3), 216-221. doi:10.2471/BLT.09.065649
Gilroy, K. E., Callaghan-Koru, J. A., Cardemil, C. V., Nsona, H., Amouzou, A., Mtimuni, A., . . . Group, C. C.-M. Q. o. C. W. (2013). Quality of sick child care delivered by Health Surveillance Assistants in Malawi. Health Policy Plan, 28(6), 573-585. doi:10.1093/heapol/czs095
Goal/Ethiopia. (2011). Sidama Child Survival Project/Awassa Zuriya and Boricha Waredas (districts), SidamanZone, SNNPR, Ethiopia: Final evaluation project.

Gopaldas, T., & Gujral, S. (2002). Empowering a tea-plantation community to improve its micronutrient health. Food and Nutrition Bulletin, 23(2), 143-152. doi:http://dx.doi.org/10.1177/156482650202300203

Gottlieb, J. (2007). Reducing child mortality with Vitamin A in Nepal.Case Studies in Global Health: Millions Saved. Levine, R (ed.) 2007 Jones and Bartlett

Grabowsky, M., Farrell, N., Hawley, W., Chimumbwa, J., Hoyer, S., Wolkon, A., & Selanikio, J. (2005). Integrating insecticide-treated bednets into a measles vaccination campaign achieves high, rapid and equitable coverage with direct and voucher-based methods. Trop Med Int Health, 10(11), 1151-1160. doi:10.1111/j.1365-3156.2005.01502.x

Greenwood, B. M., Bradley, A. K., Byass, P., Greenwood, A. M., Menon, A., Snow, R. W., . . . Hatib-N'Jie, A. B. (1990). Evaluation of a primary health care programme in The Gambia. II. Its impact on mortality and morbidity in young children. J Trop Med Hyg, 93(2), 87-97.

Greer, G., Akinpelumi, A., Madueke, L., Plowman, B., Fapohunda, B., Tawfik, Y., . . . Lennox, B. (2004). Improving management of chilhood malaria in Nigeria and Uganda by improving practices of patent medicine vendors. BASICS II/USAID

Gregson, S., Nyamukapa, C. A., Sherr, L., Mugurungi, O., & Campbell, C. (2013). Grassroots community organizations' contribution to the scale-up of HIV testing and counselling services in Zimbabwe. AIDS, 27(10), 1657-1666. doi:10.1097/QAD.0b013e3283601b90

Grimwood, A., Fatti, G., Mothibi, E., Malahlela, M., Shea, J., & Eley, B. (2012). Community adherence support improves programme retention in children on antiretroviral treatment: a multicentre cohort study in South Africa. J Int AIDS Soc, 15(2), 17381. doi:10.7448/IAS.15.2.17381

Grobusch, M. P., Lell, B., Schwarz, N. G., Gabor, J., Dornemann, J., Potschke, M., . . . Kremsner, P. G. (2007). Intermittent preventive treatment against malaria in infants in Gabon--a randomized, double-blind, placebo-controlled trial. J Infect Dis, 196(11), 1595-1602. doi:10.1086/522160

Gupta, D. N., Mondal, S. K., Ghosh, S., Rajendran, K., Sur, D., & Manna, B. (2003). Impact of zinc supplementation on diarrhoeal morbidity in rural children of West Bengal, India. Acta Paediatr, 92(5), 531-536. doi:http://dx.doi.org/10.1111/j.1651-2227.2003.tb02501.x

Gupta, D. N., Rajendran, K., Mondal, S. K., Ghosh, S., & Bhattacharya, S. K. (2007). Operational feasibility of implementing community-based zinc supplementation: impact on childhood diarrheal morbidity. Pediatr Infect Dis J, 26(4), 306-310. doi:10.1097/01.inf.0000258692.65485.d9

Gupta, N., Cyamatare, F. R., Niyigena, P., Niyigena, J. W., Stulac, S., Mugwaneza, P., . . . Franke, M. F. (2013). Clinical outcomes of a comprehensive integrated program for HIV-exposed infants: a 3-year experience promoting HIV-free survival in rural Rwanda. J Acquir Immune Defic Syndr, 62(4), e109-114. doi:10.1097/QAI.0b013e31827d5118

Guyon, A., Rambeloson, Z., Hainsworth, M., & Quinn, V. (2004). Assesing a behavior change strategy for The Essential Nutrition Actions, Immunization and Family Planning: Antananarivo and Fianarantsoa Provinces, Madagascar.

Habib, M. A., Soofi, S., Sadiq, K., Samejo, T., Hussain, M., Mirani, M., . . . Bhutta, Z. A. (2013). A study to evaluate the acceptability, feasibility and impact of packaged interventions ("Diarrhea Pack") for prevention and treatment of childhood diarrhea in rural Pakistan. BMC Public Health, 13, 922. doi:10.1186/1471-2458-13-922

Habluetzel, A., Diallo, D. A., Esposito, F., Lamizana, L., Pagnoni, F., Lengeler, C., . . . Cousens, S. N. (1997). Do insecticide-treated curtains reduce all-cause child mortality in Burkina Faso? Trop Med Int Health, 2(9), 855-862. doi:http://dx.doi.org/10.1046/j.1365-3156.1997.d01-413.x

Hadi, A. (2002). Integrating prevention of acute respiratory infections with micro-credit programme: experience of BRAC, Bangladesh. Public Health, 116(4), 238-244. doi:10.1038/sj.ph.1900863

Hadi, A. (2003). Management of acute respiratory infections by community health volunteers: experience of Bangladesh Rural Advancement Committee (BRAC). Bull World Health Organ, 81(3), 183-189.

Haggerty, P. A., Muladi, K., Kirkwood, B. R., Ashworth, A., & Manunebo, M. (1994). Community-based hygiene education to reduce diarrhoeal disease in rural Zaire: impact of the intervention on

diarrhoeal morbidity. Int J Epidemiol, 23(5), 1050-1059. doi:http://dx.doi.org/10.1093/ije/23.5.1050
Hale, L., DaVanzo, J., Razzaque, A., & Rahman, M. (2006). Why are infant and child mortality rates lower in the MCH-FP area of Matlab, Bangladesh? Stud Fam Plann, 37(4), 281-292. doi:http://dx.doi.org/10.1111/j.1728-4465.2006.00106.x
Hamainza, B., Moonga, H., Sikaala, C. H., Kamuliwo, M., Bennett, A., Eisele, T. P., . . . Killeen, G. F. (2014). Monitoring, characterization and control of chronic, symptomatic malaria infections in rural Zambia through monthly household visits by paid community health workers. Malar J, 13, 128. doi:10.1186/1475-2875-13-128
Hamer, D. H., Brooks, E. T., Semrau, K., Pilingana, P., MacLeod, W. B., Siazeele, K., . . . Yeboah-Antwi, K. (2012). Quality and safety of integrated community case management of malaria using rapid diagnostic tests and pneumonia by community health workers. Pathog Glob Health, 106(1), 32-39. doi:10.1179/1364859411Y.0000000042
Han, A. M., & Hlaing, T. (1989). Prevention of diarrhoea and dysentery by hand washing. Trans R Soc Trop Med Hyg, 83, 128-131. doi:http://dx.doi.org/10.1016/0035-9203(89)90737-2
Harkins, T., Drasbek, C., Arroyo, J., & McQuestion, M. (2008). The health benefits of social mobilization: experiences with community-based Integrated Management of Childhood Illness in Chao, Peru and San Luis, Honduras. Promot Educ, 15(2), 15-20. doi:10.1177/1025382308090340
Havemann, K., Pridmore, P., Tomkins, A., & Garn, K. D. (2013). What works and why? Evaluation of a community nutrition programme in Kenya. Public Health Nutr, 16(9), 1614-1621. doi:10.1017/S1368980012004880
Haver, J., Brieger, W., Zoungrana, J., Ansari, N., & Kagoma, J. (2015). Experiences engaging community health workers to provide maternal and newborn health services: implementation of four programs. Int J Gynaecol Obstet, 130 Suppl 2, S32-39. doi:10.1016/j.ijgo.2015.03.006
Hawkes, M., Katsuva, J. P., & Masumbuko, C. K. (2009). Use and limitations of malaria rapid diagnostic testing by community health workers in war-torn Democratic Republic of Congo. Malar J, 8, 308. doi:10.1186/1475-2875-8-308
Helen Keller International/Nepal (2012). Action against malnutrition through agriculture: Nepal Child Survival Project, Kailali and Baitadi districts, Far Western Region, Bajura expansion district.
Herrera, M. G., Nestel, P., el Amin, A., Fawzi, W. W., Mohamed, K. A., & Weld, L. (1992). Vitamin A supplementation and child survival. Lancet, 340(8814), 267-271. doi:http://dx.doi.org/10.1016/0140-6736(92)92357-I
Hodgins, S., McPherson, R., Suvedi, B. K., Shrestha, R. B., Silwal, R. C., Ban, B., . . . Baqui, A. H. (2010). Testing a scalable community-based approach to improve maternal and neonatal health in rural Nepal. J Perinatol, 30(6), 388-395. doi:10.1038/jp.2009.181
Holt, E. A., Boulos, R., Halsey, N. A., Boulos, L. M., & Boulos, C. (1990). Childhood survival in Haiti: protective effect of measles vaccination. Pediatrics, 85(2), 188-194.
Hossain, S. M., Duffield, A., & Taylor, A. (2005). An evaluation of the impact of a US$60 million nutrition programme in Bangladesh. Health Policy Plan, 20(1), 35-40. doi:10.1093/heapol/czi004
Houeto, D., & Deccache, A. (2007). Child malaria in sub-saharan Africa: effective control and prevention require a health promotion approach. Int Q Community Health Educ, 28(1), 51-62. doi:10.2190/IQ.28.1.e
Hung le, Q., Vries, P. J., Giao, P. T., Nam, N. V., Binh, T. Q., Chong, M. T., . . . Kager, P. A. (2002). Control of malaria: a successful experience from Viet Nam. Bull World Health Organ, 80(8), 660-666.
Husein, K., Adeyi, O., Bryant, J., & Cara, N. B. (1993). Developing a primary health care management information system that supports the pursuit of equity, effectiveness and affordability. Soc Sci Med, 36(5), 585-596. doi:http://dx.doi.org/10.1016/0277-9536(93)90055-9
Jakobsen, M. S., Sodemann, M., Biai, S., Nielsen, J., & Aaby, P. (2008). Promotion of exclusive breastfeeding is not likely to be cost effective in West Africa. A randomized intervention study from Guinea-Bissau. Acta Paediatr, 97(1), 68-75. doi:10.1111/j.1651-2227.2007.00532.x

Johnson, A. D., Thomson, D. R., Atwood, S., Alley, I., Beckerman, J. L., Kone, I., . . . Mukherjee, J. (2013). Assessing early access to care and child survival during a health system strengthening intervention in Mali: a repeated cross sectional survey. PLoS One, 8(12), e81304. doi:10.1371/journal.pone.0081304

Kachur, S. P., Phillips-Howard, P. A., Odhacha, A. M., Ruebush, T. K., Oloo, A. J., & Nahlen, B. L. (1999). Maintenance and sustained use of insecticide-treated bednets and curtains three years after a controlled trial in western Kenya. Trop Med Int Health, 4(11), 728-735. doi:http://dx.doi.org/10.1046/j.1365-3156.1999.00481.x

Kagaayi, J., Dreyfuss, M. L., Kigozi, G., Chen, M. Z., Wabwire-Mangen, F., Serwadda, D., . . . Gray, R. H. (2005). Maternal self-medication and provision of nevirapine to newborns by women in Rakai, Uganda. J Acquir Immune Defic Syndr, 39(1), 121-124. doi:http://dx.doi.org/10.1097/01.qai.0000148530.66587.7c

Kalyango, J. N., Alfven, T., Peterson, S., Mugenyi, K., Karamagi, C., & Rutebemberwa, E. (2013). Integrated community case management of malaria and pneumonia increases prompt and appropriate treatment for pneumonia symptoms in children under five years in Eastern Uganda. Malar J, 12, 340. doi:10.1186/1475-2875-12-340

Kalyango, J. N., Rutebemberwa, E., Alfven, T., Ssali, S., Peterson, S., & Karamagi, C. (2012). Performance of community health workers under integrated community case management of childhood illnesses in eastern Uganda. Malar J, 11, 282. doi:10.1186/1475-2875-11-282

Kapil, U., & Pradhan, R. (1999). Integrated Child Development Services scheme (ICDS) and its impact on nutritional status of children in India and recent initiatives. Indian J Public Health, 43(1), 21-25.

Kark, S. L., & Cassel, J. (1952). The Pholela Health Centre; a progress report. S Afr Med J, 26(7), 131-136; concl. doi:http://dx.doi.org/10.2105/ajph.92.11.1743

Kaye, K., Khan, N. H., Hossain, A., & Bangladesh, S. t. C. (1994, Oct 2-7, 1994). Effect of a nutrition education program on the weight of younger siblings of malnourished children in Bangladesh. Paper presented at the Community Impact of PVO Child Survival Efforts: 1985-1994, Bangalore, Karnataka, India.

Keating, J., Hutchinson, P., Miller, J. M., Bennett, A., Larsen, D. A., Hamainza, B., . . . Eisele, T. P. (2012). A quasi-experimental evaluation of an interpersonal communication intervention to increase insecticide-treated net use among children in Zambia. Malar J, 11, 313. doi:10.1186/1475-2875-11-313

Keoprasith, B., Kizuki, M., Watanabe, M., & Takano, T. (2013). The impact of community-based, workshop activities in multiple local dialects on the vaccination coverage, sanitary living and the health status of multiethnic populations in Lao PDR. Health Promot Int, 28(3), 453-465. doi:10.1093/heapro/das030

Khan, A. J., Khan, J. A., Akbar, M., & Addiss, D. G. (1990). Acute respiratory infections in children: a case management intervention in Abbottabad District, Pakistan. Bull World Health Organ, 68(5), 577-585.

Khan, M. U. (1982). Interruption of shigellosis by hand washing. Trans R Soc Trop Med Hyg, 76(2), 164-168. doi:http://dx.doi.org/10.1016/0035-9203(82)90266-8

Khan, N. C., Khoi, H. H., Giay, T., Nhan, N. T., Nhan, T. T., Dung, N. C., . . . Luy, H. T. (2002). Control of Vitamin A deficiency in Vietnam: Achievements and future orientation. Food and Nutrition Bulletin, 23(2). doi:http://dx.doi.org/10.1177/156482650202300202

Khandekar, R., Ton, T. K., & Do Thi, P. (2006). Impact of face washing and environmental improvement on reduction of active trachoma in Vietnam-a public health intervention study. Ophthalmic Epidemiol, 13(1), 43-52. doi:10.1080/09286580500477507

Kidane, G., & Morrow, R. H. (2000). Teaching mothers to provide home treatment of malaria in Tigray, Ethiopia: a randomised trial. Lancet, 356(9229), 550-555. doi:10.1016/S0140-6736(00)02580-0

Kielmann, A. A., Mobarak, A. B., Hammamy, M. T., Gomaa, A. I., Abou-el-Saad, S., Lotfi, R. K., . . . Nagaty, A. (1985). Control of deaths from diarrheal disease in rural communities. I. Design of an intervention study and effects on child mortality. Trop Med Parasitol, 36(4), 191-198.

Kielmann, A. A., Taylor, C. E., DeSweemer, C., Uberoi, I. S., Takulia, H. S., Masih, N., & Vohra, S. (1978). The Narangwal experiment on interactions of nutrition and infections : II. Morbidity and mortality effects. Indian J Med Res, 68 Suppl, 21-41.

Kim, M. H., Ahmed, S., Buck, W. C., Preidis, G. A., Hosseinipour, M. C., Bhalakia, A., . . . Kline, M. W. (2012). The Tingathe programme: a pilot intervention using community health workers to create a continuum of care in the prevention of mother to child transmission of HIV (PMTCT) cascade of services in Malawi. J Int AIDS Soc, 15 Suppl 2, 17389. doi:10.7448/IAS.15.4.17389

King, C., McCollum, E. D., Mankhambo, L., Colbourn, T., Beard, J., Hay Burgess, D. C., . . . Mukanga, D. (2015). Can We Predict Oral Antibiotic Treatment Failure in Children with Fast-Breathing Pneumonia Managed at the Community Level? A Prospective Cohort Study in Malawi. PLoS One, 10(8), e0136839. doi:10.1371/journal.pone.0136839

Kirchhoff, L. V., McClelland, K. E., Do Carmo Pinho, M., Araujo, J. G., De Sousa, M. A., & Guerrant, R. L. (1985). Feasibility and efficacy of in-home water chlorination in rural North-eastern Brazil. J Hyg (Lond), 94(2), 173-180. doi:http://dx.doi.org/10.1017/s0022172400061374

Kisia, J., Nelima, F., Otieno, D. O., Kiilu, K., Emmanuel, W., Sohani, S., . . . Akhwale, W. (2012). Factors associated with utilization of community health workers in improving access to malaria treatment among children in Kenya. Malar J, 11, 248. doi:10.1186/1475-2875-11-248

Kisinza, W. N., Kisoka, W. J., Mutalemwa, P. P., Njau, J., Tenu, F., Nkya, T., . . . Magesa, S. M. (2008). Community directed interventions for malaria, tuberculosis and vitamin A in onchocerciasis endemic districts of Tanzania. Tanzan J Health Res, 10(4), 232-239. doi:http://dx.doi.org/10.4314/thrb.v10i4.45079

Klemm, R. D., Labrique, A. B., Christian, P., Rashid, M., Shamim, A. A., Katz, J., . . . West, K. P., Jr. (2008). Newborn vitamin A supplementation reduced infant mortality in rural Bangladesh. Pediatrics, 122(1), e242-250. doi:10.1542/peds.2007-3448

Knippenberg, R., Alihonou, E., Soucat, A., Oyegbite, K., Calivis, M., Hopwood, I., . . . Ofosu-Amaah, S. (1997). Implementation of the Bamako Initiative: strategies in Benin and Guinea. Int J Health Plann Manage, 12 Suppl 1, S29-47. doi:10.1002/(SICI)1099-1751(199706)12:1+<S29::AID-HPM465>3.0.CO;2-U

Koenig, M. A., Fauveau, V., & Wojtyniak, B. (1991). Mortality reductions from health interventions: The case of immunization in Bangladesh. Population and Development Review, 17(1), 87-104. doi:http://dx.doi.org/10.2307/1972353

Kounnavong, S., Sunahara, T., Mascie-Taylor, C. G., Hashizume, M., Okumura, J., Moji, K., . . . Yamamoto, T. (2011). Effect of daily versus weekly home fortification with multiple micronutrient powder on haemoglobin concentration of young children in a rural area, Lao People's Democratic Republic: a randomised trial. Nutr J, 10, 129. doi:10.1186/1475-2891-10-129

Kouyate, B., Some, F., Jahn, A., Coulibaly, B., Eriksen, J., Sauerborn, R., . . . Mueller, O. (2008). Process and effects of a community intervention on malaria in rural Burkina Faso: randomized controlled trial. Malar J, 7, 50. doi:10.1186/1475-2875-7-50

Kroeger, A., Meyer, R., Mancheno, M., Gonzalez, M., & Pesse, K. (1997). Operational aspects of bednet impregnation for community-based malaria control in Nicaragua, Ecuador, Peru and Colombia. Trop Med Int Health, 2(6), 589-602. doi:http://dx.doi.org/10.1046/j.1365-3156.1997.d01-319.x

Lamb, W. H., Foord, F. A., Lamb, C. M., & Whitehead, R. G. (1984). Changes in maternal and child mortality rates in three isolated Gambian villages over ten years. Lancet, 2(8408), 912-914. doi:http://dx.doi.org/10.1016/s0140-6736(84)90664-0

Langston, A., Weiss, J., Landegger, J., Pullum, T., Morrow, M., Kabadege, M., . . . Sarriot, E. (2014). Plausible role for CHW peer support groups in increasing care-seeking in an integrated community case management project in Rwanda: a mixed methods evaluation. Glob Health Sci Pract, 2(3), 342-354. doi:10.9745/GHSP-D-14-00067

Lartey, A., Manu, A., Brown, K. H., Peerson, J. M., & Dewey, K. G. (1999). A randomized, community-based trial of the effects of improved, centrally processed complementary foods on growth and micronutrient status of Ghanaian infants from 6 to 12 mo of age. Am J Clin Nutr, 70(3), 391-404.

le Roux, I. M., Rotheram-Borus, M. J., Stein, J., & Tomlinson, M. (2014). The impact of paraprofessional home visitors on infants' growth and health at 18 months. Vulnerable Child Youth Stud, 9(4), 291-304. doi:10.1080/17450128.2014.940413

Lema, I. A., Sando, D., Magesa, L., Machumi, L., Mungure, E., Mwanyika Sando, M., . . . Barnighausen, T. (2014). Community health workers to improve antenatal care and PMTCT uptake in Dar es Salaam, Tanzania: a quantitative performance evaluation. J Acquir Immune Defic Syndr, 67 Suppl 4, S195-201. doi:10.1097/QAI.0000000000000371

Lemma, H., Byass, P., Desta, A., Bosman, A., Costanzo, G., Toma, L., . . . Barnabas, G. A. (2010). Deploying artemether-lumefantrine with rapid testing in Ethiopian communities: impact on malaria morbidity, mortality and healthcare resources. Trop Med Int Health, 15(2), 241-250. doi:10.1111/j.1365-3156.2009.02447.x

Lengeler, C., Armstrong-Schellenberg, J., D'Alessandro, U., Binka, F., & Cattani, J. (1998). Relative versus absolute risk of dying reduction after using insecticide-treated nets for malaria control in Africa. Trop Med Int Health, 3(4), 286-290. doi:http://dx.doi.org/10.1046/j.1365-3156.1998.00236.x

Levinson, F. J., Barney, J., Bassett, L., & Schultink, W. (2007). Utilization of positive deviance analysis in evaluating community-based nutrition programs: an application to the Dular program in Bihar, India. Food Nutr Bull, 28(3), 259-265. doi:http://dx.doi.org/10.1177/156482650702800301

Levy-Bruhl, D., Soucat, A., Osseni, R., Ndiaye, J. M., Dieng, B., De Bethune, X., . . . Knippenberg, R. (1997). The Bamako Initiative in Benin and Guinea: improving the effectiveness of primary health care. Int J Health Plann Manage, 12 Suppl 1, S49-79. doi:10.1002/(SICI)1099-1751(199706)12:1+<S49::AID-HPM466>3.0.CO;2-P

Lindblade, K. A., Eisele, T. P., Gimnig, J. E., Alaii, J. A., Odhiambo, F., ter Kuile, F. O., . . . Slutsker, L. (2004). Sustainability of reductions in malaria transmission and infant mortality in western Kenya with use of insecticide-treated bednets: 4 to 6 years of follow-up. JAMA, 291(21), 2571-2580. doi:10.1001/jama.291.21.2571

Linkins, R. W., Mansour, E., Wassif, O., Hassan, M. H., & Patriarca, P. A. (1995). Evaluation of house-to-house versus fixed-site oral poliovirus vaccine delivery strategies in a mass immunization campaign in Egypt. Bull World Health Organ, 73(5), 589-595.

Linn, A. M., Ndiaye, Y., Hennessee, I., Gaye, S., Linn, P., Nordstrom, K., & McLaughlin, M. (2015). Reduction in symptomatic malaria prevalence through proactive community treatment in rural Senegal. Trop Med Int Health, 20(11), 1438-1446. doi:10.1111/tmi.12564

Littrell, M., Moukam, L. V., Libite, R., Youmba, J. C., & Baugh, G. (2013). Narrowing the treatment gap with equitable access: mid-term outcomes of a community case management program in Cameroon. Health Policy Plan, 28(7), 705-716. doi:10.1093/heapol/czs110

Livingston, A., Tomedi, A., Campbell, A., Morales, C., & Mwanthi, M. A. (2013). A community health worker home visitation project to prevent neonatal deaths in Kenya. J Trop Pediatr, 59(1), 64-66. doi:10.1093/tropej/fms034

Lopes, S. C., Cabral, A. J., & de Sousa, B. (2014). Community health workers: to train or to restrain? A longitudinal survey to assess the impact of training community health workers in the Bolama Region, Guinea-Bissau. Hum Resour Health, 12, 8. doi:10.1186/1478-4491-12-8

Luabeya, K. K., Mpontshane, N., Mackay, M., Ward, H., Elson, I., Chhagan, M., . . . Bennish, M. L. (2007). Zinc or multiple micronutrient supplementation to reduce diarrhea and respiratory disease in South African children: a randomized controlled trial. PLoS One, 2(6), e541. doi:10.1371/journal.pone.0000541

Luby, S. P., Agboatwalla, M., Feikin, D. R., Painter, J., Billhimer, W., Altaf, A., & Hoekstra, R. M. (2005). Effect of handwashing on child health: a randomised controlled trial. Lancet, 366(9481), 225-233. doi:10.1016/S0140-6736(05)66912-7

Luby, S. P., Agboatwalla, M., Painter, J., Altaf, A., Billhimer, W. L., & Hoekstra, R. M. (2004). Effect of intensive handwashing promotion on childhood diarrhea in high-risk communities in Pakistan: a randomized controlled trial. JAMA, 291(21), 2547-2554. doi:10.1001/jama.291.21.2547

Lugada, E., Levin, J., Abang, B., Mermin, J., Mugalanzi, E., Namara, G., . . . Bunnell, R. (2010). Comparison of home and clinic-based HIV testing among household members of persons taking antiretroviral therapy in Uganda: results from a randomized trial. J Acquir Immune Defic Syndr, 55(2), 245-252. doi:10.1097/QAI.0b013e3181e9e069

Lundeen, E., Schueth, T., Toktobaev, N., Zlotkin, S., Hyder, S. M., & Houser, R. (2010). Daily use of Sprinkles micronutrient powder for 2 months reduces anemia among children 6 to 36 months of age in the Kyrgyz Republic: a cluster-randomized trial. Food Nutr Bull, 31(3), 446-460. doi:http://dx.doi.org/10.1177/156482651003100307

Lutter, C. K., Rodriguez, A., Fuenmayor, G., Avila, L., Sempertegui, F., & Escobar, J. (2008). Growth and micronutrient status in children receiving a fortified complementary food. J Nutr, 138(2), 379-388.

Lye, M. S., Nair, R. C., Choo, K. E., Kaur, H., & Lai, K. P. (1996). Acute respiratory tract infection: a community-based intervention study in Malaysia. J Trop Pediatr, 42(3), 138-143. doi:http://dx.doi.org/10.1093/tropej/42.3.138

Lynch, M., West, S., Munoz, B., Frick, K. D., & Mkocha, H. A. (2003). Azithromycin treatment coverage in Tanzanian children using community volunteers. Ophthalmic Epidemiol, 10(3), 167-175. doi:http://dx.doi.org/10.1076/opep.10.3.167.15082

Mackintosh, U. A. T., Marsh, D. R., & Schroder, D. G. (2002). Sustained positive deviant child care practices and their effects on child growth in Viet Nam. Food and Nutrition Bulletin, 23(4), 16-24. doi:http://dx.doi.org/10.1177/15648265020234s204

Magbity, E. B., Marbiah, N. T., Maude, G., Curtis, C. F., Bradley, D. J., Greenwood, B. M., . . . Lines, J. D. (1997). Effects of community-wide use of lambdacyhalothrin-impregnated bednets on malaria vectors in rural Sierra Leone. Med Vet Entomol, 11(1), 79-86. doi:http://dx.doi.org/10.1111/j.1365-2915.1997.tb00293.x

Magnani, R. J., Rice, J. C., Mock, N. B., Abdoh, A. A., Mercer, D. M., & Tankari, K. (1996). The impact of primary health care services on under-five mortality in rural Niger. Int J Epidemiol, 25(3), 568-577. doi:http://dx.doi.org/10.1093/ije/25.3.568

Management Sciences for Health/REACH (2006). Rural expansion of Afganistan's community-based healthcare program: Measuring program outcomes through household surveys.

Manandhar, D. S., Osrin, D., Shrestha, B. P., Mesko, N., Morrison, J., Tumbahangphe, K. M., . . . Members of the MIRA Makwanpur trial team. (2004). Effect of a participatory intervention with women's groups on birth outcomes in Nepal: cluster-randomised controlled trial. Lancet, 364(9438), 970-979. doi:10.1016/S0140-6736(04)17021-9

Marek, T., Diallo, I., Ndiaye, B., & Rakotosalama, J. (1999). Successful contracting of prevention services: fighting malnutrition in Senegal and Madagascar. Health Policy Plan, 14(4), 382-389. doi:http://dx.doi.org/10.1093/heapol/14.4.382

Marfin, A. A., Moore, J., Collins, C., Biellik, R., Kattel, U., Toole, M. J., & Moore, P. S. (1994). Infectious disease surveillance during emergency relief to Bhutanese refugees in Nepal. JAMA, 272(5), 377-381. doi:http://dx.doi.org/10.1001/jama.272.5.377

Marsh, V. M., Mutemi, W. M., Muturi, J., Haaland, A., Watkins, W. M., Otieno, G., & Marsh, K. (1999). Changing home treatment of childhood fevers by training shop keepers in rural Kenya. Trop Med Int Health, 4(5), 383-389. doi:http://dx.doi.org/10.1046/j.1365-3156.1999.00403.x

Martin, K. (2008). Overview of Asha/India

Matomora, M. K. (1989). A people-centered approach to primary health care implementation in Mvumi, Tanzania. Soc Sci Med, 28(10), 1031-1037. doi:http://dx.doi.org/10.1016/0277-9536(89)90385-7

Matovu, F., Nanyiti, A., & Rutebemberwa, E. (2014). Household health care-seeking costs: experiences from a randomized, controlled trial of community-based malaria and pneumonia treatment among under-fives in eastern Uganda. Malar J, 13, 222. doi:10.1186/1475-2875-13-222

Mayhew, M., Ickx, P., Stanekzai, H., Mashal, T., & Newbrander, W. (2014). Improving nutrition in Afghanistan through a community-based growth monitoring and promotion programme: a pre-post evaluation in five districts. Glob Public Health, 9 Suppl 1, S58-75. doi:10.1080/17441692.2014.917194

Mazumder, S., Taneja, S., Bahl, R., Mohan, P., Strand, T. A., Sommerfelt, H., . . . Childhood Illness Evaluation Study, G. (2014). Effect of implementation of integrated management of neonatal and childhood illness programme on treatment seeking practices for morbidities in infants: cluster randomised trial. BMJ, 349, g4988. doi:10.1136/bmj.g4988

Mazumder, S., Taneja, S., Bhandari, N., Dube, B., Agarwal, R. C., Mahalanabis, D., . . . Black, R. E. (2010). Effectiveness of zinc supplementation plus oral rehydration salts for diarrhoea in infants aged less than 6 months in Haryana state, India. Bull World Health Organ, 88(10), 754-760. doi:10.2471/BLT.10.075986

Mbonye, A. K., Bygbjerg, I. C., & Magnussen, P. (2008). Intermittent preventive treatment of malaria in pregnancy: a new delivery system and its effect on maternal health and pregnancy outcomes in Uganda. Bull World Health Organ, 86(2), 93-100. doi:http://dx.doi.org/10.2471/blt.07.041822

Mbonye, A. K., Bygbjerg, I., & Magnussen, P. (2007). Intermittent preventive treatment of malaria in pregnancy: evaluation of a new delivery approach and the policy implications for malaria control in Uganda. Health Policy, 81(2-3), 228-241. doi:10.1016/j.healthpol.2006.05.018

Mbonye, A. K., Hansen, K. S., Bygbjerg, I. C., & Magnussen, P. (2008). Intermittent preventive treatment of malaria in pregnancy: the incremental cost-effectiveness of a new delivery system in Uganda. Trans R Soc Trop Med Hyg, 102(7), 685-693. doi:10.1016/j.trstmh.2008.04.016

Mbonye, A. K., Schultz Hansen, K., Bygbjerg, I. C., & Magnussen, P. (2008). Effect of a community-based delivery of intermittent preventive treatment of malaria in pregnancy on treatment seeking for malaria at health units in Uganda. Public Health, 122(5), 516-525. doi:10.1016/j.puhe.2007.07.024

McCord, C., & Kielmann, A. A. (1978). A successful programme for medical auxiliaries treating childhood diarrhoea and pneumonia. Trop Doct, 8(4), 220-225.

McGuigan, K. G., Samaiyar, P., du Preez, M., & Conroy, R. M. (2011). High compliance randomized controlled field trial of solar disinfection of drinking water and its impact on childhood diarrhea in rural Cambodia. Environ Sci Technol, 45(18), 7862-7867. doi:10.1021/es201313x

Medical Team International/Liberia (2008). Grand Cape Mount child survival project: Improved child health in a transitional state through IMCI.

Mehta, S., Mugusi, F. M., Bosch, R. J., Aboud, S., Chatterjee, A., Finkelstein, J. L., . . . Fawzi, W. W. (2011). A randomized trial of multivitamin supplementation in children with tuberculosis in Tanzania. Nutr J, 10, 120. doi:10.1186/1475-2891-10-120

Melville, B., Fidler, T., Mehan, D., Bernard, E., & Mullings, J. (1995). Growth monitoring: the role of community health volunteers. Public Health, 109(2), 111-116. doi:http://dx.doi.org/10.1016/s0033-3506(05)80004-6

Mercer, A., Khan, M. H., Daulatuzzaman, M., & Reid, J. (2004). Effectiveness of an NGO primary health care programme in rural Bangladesh: evidence from the management information system. Health Policy Plan, 19(4), 187-198. doi:http://dx.doi.org/10.1093/heapol/czh024

Mermin, J., Were, W., Ekwaru, J. P., Moore, D., Downing, R., Behumbiize, P., . . . Bunnell, R. (2008). Mortality in HIV-infected Ugandan adults receiving antiretroviral treatment and survival of their HIV-uninfected children: a prospective cohort study. Lancet, 371(9614), 752-759. doi:10.1016/S0140-6736(08)60345-1

Migele, J., Ombeki, S., Ayalo, M., Biggerstaff, M., & Quick, R. (2007). Diarrhea prevention in a Kenyan school through the use of a simple safe water and hygiene intervention. Am J Trop Med Hyg, 76(2), 351-353.

Miller, L. C., Joshi, N., Lohani, M., Rogers, B., Loraditch, M., Houser, R., . . . Mahato, S. (2014). Community development and livestock promotion in rural Nepal: effects on child growth and health. Food Nutr Bull, 35(3), 312-326. doi:http://dx.doi.org/10.1177/156482651403500304

Miller, P., & Hirschhorn, N. (1995). The effect of a national control of diarrheal diseases program on mortality: the case of Egypt. Soc Sci Med, 40(10), S1-S30. doi:http://dx.doi.org/10.1016/0277-9536(95)00001-n

Minnesota International Health Volunteers/Uganda (2004). Improving malaria case management in Uganda communities: Lessons from the field.

Minnesota International Health Volunteers/Uganda (2004). Uganda family planning programs: Lessons from the field.

MkNelly, B., & Dunford, C. (1998). Impact of credit with education on mother and their young children's nutrition: Lower Pra rural bank credit with education program in Ghana. Freedom from hunger Research Paper no. 4

Mockenhaupt, F. P., Reither, K., Zanger, P., Roepcke, F., Danquah, I., Saad, E., . . . Bienzle, U. (2007). Intermittent preventive treatment in infants as a means of malaria control: a randomized, double-blind, placebo-controlled trial in northern Ghana. Antimicrob Agents Chemother, 51(9), 3273-3281. doi:10.1128/AAC.00513-07

Moir, J. S., Tulloch, J. L., Vrbova, H., Jolley, D. J., Heywood, P. F., & Alpers, M. P. (1985). The role of voluntary village aides in the control of malaria by presumptive treatment of fever. 1. Selection, training and practice. P N G Med J, 28(4), 257-266.
Morris, S. S., Flores, R., Olinto, P., & Medina, J. M. (2004). Monetary incentives in primary health care and effects on use and coverage of preventive health care interventions in rural Honduras: cluster randomised trial. Lancet, 364(9450), 2030-2037. doi:10.1016/S0140-6736(04)17515-6
Moseson, H., Hamad, R., & Fernald, L. (2014). Microcredit participation and child health: results from a cross-sectional study in Peru. J Epidemiol Community Health, 68(12), 1175-1181. doi:10.1136/jech-2014-204071
Moyo, S., Verver, S., Hawkridge, A., Geiter, L., Hatherill, M., Workman, L., . . . South African Tuberculosis Vaccine Initiative Neonatal Study, T. (2012). Tuberculosis case finding for vaccine trials in young children in high-incidence settings: a randomised trial. Int J Tuberc Lung Dis, 16(2), 185-191. doi:10.5588/ijtld.11.0348
Mtango, F. D., & Neuvians, D. (1986). Acute respiratory infections in children under five years. Control project in Bagamoyo District, Tanzania. Trans R Soc Trop Med Hyg, 80(6), 851-858. doi:http://dx.doi.org/10.1016/0035-9203(86)90241-5
Mubi, M., Janson, A., Warsame, M., Martensson, A., Kallander, K., Petzold, M. G., . . . Bjorkman, A. (2011). Malaria rapid testing by community health workers is effective and safe for targeting malaria treatment: randomised cross-over trial in Tanzania. PLoS One, 6(7), e19753. doi:10.1371/journal.pone.0019753
Mugeni, C., Levine, A. C., Munyaneza, R. M., Mulindahabi, E., Cockrell, H. C., Glavis-Bloom, J., . . . Binagwaho, A. (2014). Nationwide implementation of integrated community case management of childhood illness in Rwanda. Glob Health Sci Pract, 2(3), 328-341. doi:10.9745/GHSP-D-14-00080
Muhilal, Permeisih, D., Idjradinata, Y. R., Muherdiyantiningsih, & Karyadi, D. (1988). Vitamin A-fortified monosodium glutamate and health, growth, and survival of children: a controlled field trial. Am J Clin Nutr, 48(5), 1271-1276.
Mukanga, D., Babirye, R., Peterson, S., Pariyo, G. W., Ojiambo, G., Tibenderana, J. K., . . . Kallander, K. (2011). Can lay community health workers be trained to use diagnostics to distinguish and treat malaria and pneumonia in children? Lessons from rural Uganda. Trop Med Int Health, 16(10), 1234-1242. doi:10.1111/j.1365-3156.2011.02831.x
Muller, O., Traore, C., Kouyate, B., Ye, Y., Frey, C., Coulibaly, B., & Becher, H. (2006). Effects of insecticide-treated bednets during early infancy in an African area of intense malaria transmission: a randomized controlled trial. Bull World Health Organ, 84(2), 120-126. doi:/S0042-96862006000200012
Mustaphi, P., & Dobe, M. (2005). Positive deviance--the West Bengal experience. Indian J Public Health, 49(4), 207-213.
Naheed, A., Walker Fischer, C. L., Mondal, D., Ahmed, S., Arifeen, S. E., Yunus, M., . . . Baqui, A. H. (2009). Zinc therapy for diarrhoea improves growth among Bangladeshi infants 6 to 11 months of age. J Pediatr Gastroenterol Nutr, 48(1), 89-93. doi:10.1097/MPG.0b013e31817f0182
Nanan, D., White, F., Azam, I., Afsar, H., & Hozhabri, S. (2003). Evaluation of a water, sanitation, and hygiene education intervention on diarrhoea in northern Pakistan. Bull World Health Organ, 81(3), 160-165.
Nankabirwa, V., Tylleskar, T., Nankunda, J., Engebretsen, I. M., Sommerfelt, H., Tumwine, J. K., & Consortium, P. E. R. (2011). Malaria parasitaemia among infants and its association with breastfeeding peer counselling and vitamin A supplementation: a secondary analysis of a cluster randomized trial. PLoS One, 6(7), e21862. doi:10.1371/journal.pone.0021862
Nanyonjo, A., Makumbi, F., Etou, P., Tomson, G., Kallander, K., & in, S. S. G. (2013). Perceived quality of care for common childhood illnesses: facility versus community based providers in Uganda. PLoS One, 8(11), e79943. doi:10.1371/journal.pone.0079943

Navarro, J. I., Sigulem, D. M., Ferraro, A. A., Polanco, J. J., & Barros, A. J. (2013). The double task of preventing malnutrition and overweight: a quasi-experimental community-based trial. BMC Public Health, 13, 212. doi:10.1186/1471-2458-13-212

Nevill, C. G., Some, E. S., Mung'ala, V. O., Mutemi, W., New, L., Marsh, K., . . . Snow, R. W. (1996). Insecticide-treated bednets reduce mortality and severe morbidity from malaria among children on the Kenyan coast. Trop Med Int Health, 1(2), 139-146. doi:http://dx.doi.org/10.1111/j.1365-3156.1996.tb00019.x

Newell, K. W., Duenas Lehmann, A., LeBlanc, D. R., & Garces Osorio, N. (1966). The use of toxoid for the prevention of tetanus neonatorum. Final report of a double-blind controlled field trial. Bull World Health Organ, 35(6), 863-871.

Ngasala, B. E., Malmberg, M., Carlsson, A. M., Ferreira, P. E., Petzold, M. G., Blessborn, D., . . . Martensson, A. (2011). Effectiveness of artemether-lumefantrine provided by community health workers in under-five children with uncomplicated malaria in rural Tanzania: an open label prospective study. Malar J, 10, 64. doi:10.1186/1475-2875-10-64

Nonvignon, J., Chinbuah, M. A., Gyapong, M., Abbey, M., Awini, E., Gyapong, J. O., & Aikins, M. (2012). Is home management of fevers a cost-effective way of reducing under-five mortality in Africa? The case of a rural Ghanaian District. Trop Med Int Health, 17(8), 951-957. doi:10.1111/j.1365-3156.2012.03018.x

Noor, A. M., Amin, A. A., Akhwale, W. S., & Snow, R. W. (2007). Increasing coverage and decreasing inequity in insecticide-treated bed net use among rural Kenyan children. PLoS Med, 4(8), e255. doi:10.1371/journal.pmed.0040255

Nyonator, F., Jones, T. C., Miller, R. A., Phillips, J. F., & Awoonor-Williams, J. K. (2004-2005). Guiding the Ghana community-based health planning and services approach to scaling up with qualitative systems appraisal. Int Quaterly of Community Health Education, 23(3), 189-213. doi:http://dx.doi.org/10.2190/ngm3-fydt-5827-ml1p

O'Connor, J., Lynch, M., Vitale, S., & West, S. (1999). Characteristics of effective village treatment assistants: the Kongwa Trachoma Project. Ophthalmic Epidemiol, 6(4), 257-265. doi:http://dx.doi.org/10.1076/opep.6.4.257.4186

Ohnmar, Tun, M., San, S., Than, W., & Chongsuvivatwong, V. (2012). Effects of malaria volunteer training on coverage and timeliness of diagnosis: a cluster randomized controlled trial in Myanmar. Malar J, 11, 309. doi:10.1186/1475-2875-11-309

Olupona, O. G., & Vision/Nigeria, W. (1994, Oct 2 -7, 1994). The impact of a child survival project on the health and nutrition of mothers and children in a rural nigerian local government area. Paper presented at the Community Impact of PVO Child Survival Efforts: 1985 - 1994, Bangalore, Karnataka, India.

Omer, K., Mhatre, S., Ansari, N., Laucirica, J., & Andersson, N. (2008). Evidence-based training of frontline health workers for door-to-door health promotion: a pilot randomized controlled cluster trial with Lady Health Workers in Sindh Province, Pakistan. Patient Educ Couns, 72(2), 178-185. doi:10.1016/j.pec.2008.02.018

Opryszko, M. C., Majeed, S. W., Hansen, P. M., Myers, J. A., Baba, D., Thompson, R. E., & Burnham, G. (2010). Water and hygiene interventions to reduce diarrhoea in rural Afghanistan: a randomized controlled study. J Water Health, 8(4), 687-702. doi:10.2166/wh.2010.121

O'Rourke, K., Howard-Grabman, L., & Seoane, G. (1998). Impact of community organization of women on perinatal outcomes in rural Bolivia. Rev Panam Salud Publica, 3(1), 9-14. doi:http://dx.doi.org/10.1590/s1020-49891998000100002

Osrin, D., Vaidya, A., Shrestha, Y., Baniya, R. B., Manandhar, D. S., Adhikari, R. K., . . . Costello, A. M. (2005). Effects of antenatal multiple micronutrient supplementation on birthweight and gestational duration in Nepal: double-blind, randomised controlled trial. Lancet, 365(9463), 955-962. doi:10.1016/S0140-6736(05)71084-9

Otten, M., Aregawi, M., Were, W., Karema, C., Medin, A., Bekele, W., . . . Grabowsky, M. (2009). Initial evidence of reduction of malaria cases and deaths in Rwanda and Ethiopia due to rapid scale-up of malaria prevention and treatment. Malar J, 8, 14. doi:10.1186/1475-2875-8-14

Pagnoni, F., Convelbo, N., Tiendrebeogo, J., Cousens, S., & Esposito, F. (1997). A community-based programme to provide prompt and adequate treatment of presumptive malaria in children. Trans R Soc Trop Med Hyg, 91(5), 512-517. doi:http://dx.doi.org/10.1016/s0035-9203(97)90006-7

Pandey, M. R., Daulaire, N. M., Starbuck, E. S., Houston, R. M., & McPherson, K. (1991). Reduction in total under-five mortality in western Nepal through community-based antimicrobial treatment of pneumonia. Lancet, 338(8773), 993-997. doi:http://dx.doi.org/10.1016/0140-6736(91)91847-n

Pandey, M. R., Sharma, P. R., Gubhaju, B. B., Shakya, G. M., Neupane, R. P., Gautam, A., & Shrestha, I. B. (1989). Impact of a pilot acute respiratory infection (ARI) control programme in a rural community of the hill region of Nepal. Ann Trop Paediatr, 9(4), 212-220. doi:http://dx.doi.org/10.1080/02724936.1989.11748635

Parashar, M., Singh, S., Kishore, J., Kumar, A., & Bhardwaj, M. (2013). Effect of Community-based Behavior Change Communication on Delivery and Newborn Health Care Practices in a Resettlement Colony of Delhi. Indian J Community Med, 38(1), 42-48. doi:10.4103/0970-0218.106627

Pasha, O., Del Rosso, J., Mukaka, M., & Marsh, D. (2003). The effect of providing fansidar (sulfadoxine-pyrimethamine) in schools on mortality in school-age children in Malawi. Lancet, 361(9357), 577-578. doi:http://dx.doi.org/10.1016/s0140-6736(03)12511-1

Patouillard, E., Conteh, L., Webster, J., Kweku, M., Chandramohan, D., & Greenwood, B. (2011). Coverage, adherence and costs of intermittent preventive treatment of malaria in children employing different delivery strategies in Jasikan, Ghana. PLoS One, 6(11), e24871. doi:10.1371/journal.pone.0024871

Paxman, J. M., Sayeed, A., Buxbaum, A., Huber, S. C., & Stover, C. (2005). The India Local Initiatives Program: a model for expanding reproductive and child health services. Stud Fam Plann, 36(3), 203-220. doi:http://dx.doi.org/10.1111/j.1728-4465.2005.00062.x

Paxson, C., & Schady, N. (2007). Does money matter?: The effects of cash transfers on child health and development in rural Ecuador. Retrieved from World Bank Policy Research Working Paper 4426:

Peletz, R., Simunyama, M., Sarenje, K., Baisley, K., Filteau, S., Kelly, P., & Clasen, T. (2012). Assessing water filtration and safe storage in households with young children of HIV-positive mothers: a randomized, controlled trial in Zambia. PLoS One, 7(10), e46548. doi:10.1371/journal.pone.0046548

Pence, B. W., Nyarko, P., Phillips, J. F., & Debpuur, C. (2007). The effect of community nurses and health volunteers on child mortality: the Navrongo Community Health and Family Planning Project. Scand J Public Health, 35(6), 599-608. doi:10.1080/14034940701349225

Penny, M. E., Marin, R. M., Duran, A., Peerson, J. M., Lanata, C. F., Lonnerdal, B., . . . Brown, K. H. (2004). Randomized controlled trial of the effect of daily supplementation with zinc or multiple micronutrients on the morbidity, growth, and micronutrient status of young Peruvian children. Am J Clin Nutr, 79(3), 457-465.

Penny, M. E., Peerson, J. M., Marin, R. M., Duran, A., Lanata, C. F., Lonnerdal, B., . . . Brown, K. H. (1999). Randomized, community-based trial of the effect of zinc supplementation, with and without other micronutrients, on the duration of persistent childhood diarrhea in Lima, Peru. J Pediatr, 135(2 Pt 1), 208-217. doi:http://dx.doi.org/10.1016/s0022-3476(99)70024-7

Perez, F., Ba, H., Dastagire, S. G., & Altmann, M. (2009). The role of community health workers in improving child health programmes in Mali. BMC Int Health Hum Rights, 9, 28. doi:10.1186/1472-698X-9-28

Perez-Cuevas, R., Reyes, H., Pego, U., Tome, P., Ceja, K., Flores, S., & Gutierrez, G. (1999). Immunization promotion activities: are they effective in encouraging mothers to immunize their children? Soc Sci Med, 49(7), 921-932. doi:http://dx.doi.org/10.1016/s0277-9536(99)00178-1

Perry, H. B., Shanklin, D. S., & Schroeder, D. G. (2003). Impact of a community-based comprehensive primary healthcare programme on infant and child mortality in Bolivia. J Health Popul Nutr, 21(4), 383-395.

Perry, H., Cayemittes, M., Philippe, F., Dowell, D., Dortonne, J. R., Menager, H., . . . Berggren, G. (2006). Reducing under-five mortality through Hopital Albert Schweitzer's integrated system in Haiti. Health Policy Plan, 21(3), 217-230. doi:10.1093/heapol/czl005

Perry, H., Robison, N., Chavez, D., Taja, O., Hilari, C., Shanklin, D., & Wyon, J. (1998). The census-based, impact-oriented approach: its effectiveness in promoting child health in Bolivia. Health Policy Plan, 13(2), 140-151. doi:http://dx.doi.org/10.1093/heapol/13.2.140

Phillips, J. F., Bawah, A. A., & Binka, F. N. (2006). Accelerating reproductive and child health programme impact with community-based services: the Navrongo experiment in Ghana. Bull World Health Organ, 84(12), 949-955. doi:http://dx.doi.org/10.2471/blt.06.030064

Phillips-Howard, P. A., Nahlen, B. L., Alaii, J. A., ter Kuile, F. O., Gimnig, J. E., Terlouw, D. J., . . . Hawley, W. A. (2003). The efficacy of permethrin-treated bed nets on child mortality and morbidity in western Kenya I. Development of infrastructure and description of study site. Am J Trop Med Hyg, 68(4 Suppl), 3-9.

Pitt, M. M., Khandker, S. R., Chowdhury, O. H., & Millimet, D. L. (2003). Credit programs for te poor and the health status of children in rural Bangladesh. International Economic Review, 44(1), 87-113. doi:http://dx.doi.org/10.1111/1468-2354.t01-1-00063

Plan/Cameroon. (2004). Child survival project: Final evaluation report.

Plan/Ecuador. (2003). IMCI strategy and its impact on child mortality.

Plan/Mali. (2006). Child Reach: Child Survival XVII Project, Kita District, Kayes region, Mali.

Plan/Nepal. (2011). Local innovation for better outcomes for neonates (LIBON) project: Plan Nepal Child Survival Project XXVII, Sunsari, Parsa, and Bara districts in Nepal.

Porco, T. C., Gebre, T., Ayele, B., House, J., Keenan, J., Zhou, Z., . . . Lietman, T. M. (2009). Effect of mass distribution of azithromycin for trachoma control on overall mortality in Ethiopian children: a randomized trial. JAMA, 302(9), 962-968. doi:10.1001/jama.2009.1266

Quick, R. E., Kimura, A., Thevos, A., Tembo, M., Shamputa, I., Hutwagner, L., & Mintz, E. (2002). Diarrhea prevention through household-level water disinfection and safe storage in Zambia. Am J Trop Med Hyg, 66(5), 584-589.

Quick, R. E., Venczel, L. V., Mintz, E. D., Soleto, L., Aparicio, J., Gironaz, M., . . . Tauxe, R. V. (1999). Diarrhoea prevention in Bolivia through point-of-use water treatment and safe storage: a promising new strategy. Epidemiol Infect, 122(1), 83-90. doi:http://dx.doi.org/10.1017/s0950268898001782

Rabbani, G. H., Larson, C. P., Islam, R., Saha, U. R., & Kabir, A. (2010). Green banana-supplemented diet in the home management of acute and prolonged diarrhoea in children: a community-based

trial in rural Bangladesh. Trop Med Int Health, 15(10), 1132-1139. doi:10.1111/j.1365-3156.2010.02608.x
Radhakrishna, K. V., Hemalatha, R., Geddam, J. J., Kumar, P. A., Balakrishna, N., & Shatrugna, V. (2013). Effectiveness of zinc supplementation to full term normal infants: a community based double blind, randomized, controlled, clinical trial. PLoS One, 8(5), e61486. doi:10.1371/journal.pone.0061486
Rahaman, M. M., Aziz, K. M., Patwari, Y., & Munshi, M. H. (1979). Diarrhoeal mortality in two Bangladeshi villages with and without community-based oral rehydration therapy. Lancet, 2(8147), 809-812. doi:http://dx.doi.org/10.1016/s0140-6736(79)92172-x
Rahman, F., Bose, S., Linnan, M., Rahman, A., Mashreky, S., Haaland, B., & Finkelstein, E. (2012). Cost-effectiveness of an injury and drowning prevention program in Bangladesh. Pediatrics, 130(6), e1621-1628. doi:10.1542/peds.2012-0757
Rahman, M. M., Tofail, F., Wahed, M. A., Fuchs, G. J., Baqui, A. H., & Alvarez, J. O. (2002). Short-term supplementation with zinc and vitamin A has no significant effect on the growth of undernourished Bangladeshi children. Am J Clin Nutr, 75(1), 87-91.
Rahman, M. M., Vermund, S. H., Wahed, M. A., Fuchs, G. J., Baqui, A. H., & Alvarez, J. O. (2001). Simultaneous zinc and vitamin A supplementation in Bangladeshi children: randomised double blind controlled trial. BMJ, 323(7308), 314-318. doi:http://dx.doi.org/10.1136/bmj.323.7308.314
Rahman, M., Jhohura, F. T., Mistry, S. K., Chowdhury, T. R., Ishaque, T., Shah, R., & Afsana, K. (2015). Assessing Community Based Improved Maternal Neonatal Child Survival (IMNCS) Program in Rural Bangladesh. PLoS One, 10(9), e0136898. doi:10.1371/journal.pone.0136898
Rahman, S. (1982). The effect of traditional birth attendants and tetanus toxoid in reduction of neo-natal mortality. J Trop Pediatr, 28(4), 163-165. doi:http://dx.doi.org/10.1093/tropej/28.4.163-a
Rahmathullah, L., Tielsch, J. M., Thulasiraj, R. D., Katz, J., Coles, C., Devi, S., . . . Kamaraj, C. (2003). Impact of supplementing newborn infants with vitamin A on early infant mortality: community based randomised trial in southern India. BMJ, 327(7409), 254. doi:10.1136/bmj.327.7409.254
Rahmathullah, L., Underwood, B. A., Thulasiraj, R. D., Milton, R. C., Ramaswamy, K., Rahmathullah, R., & Babu, G. (1990). Reduced mortality among children in southern India receiving a small weekly dose of vitamin A. N Engl J Med, 323(14), 929-935. doi:10.1056/NEJM199010043231401
Ram, E. R. (1977). [Integrated health services, the Miraj project in India]. Carnets Enfance, 39, 15-32.
Rasooly, M. H., Govindasamy, P., Aqil, A., Rutstein, S., Arnold, F., Noormal, B., . . . Shadoul, A. (2014). Success in reducing maternal and child mortality in Afghanistan. Glob Public Health, 9 Suppl 1, S29-42. doi:10.1080/17441692.2013.827733
Ratsimbasoa, A., Ravony, H., Vonimpaisomihanta, J. A., Raherinjafy, R., Jahevitra, M., Rapelanoro, R., . . . Menard, D. (2012). Compliance, safety, and effectiveness of fixed-dose artesunate-amodiaquine for presumptive treatment of non-severe malaria in the context of home management of malaria in Madagascar. Am J Trop Med Hyg, 86(2), 203-210. doi:10.4269/ajtmh.2012.11-0047
Ratsimbasoa, A., Ravony, H., Vonimpaisomihanta, J. A., Raherinjafy, R., Jahevitra, M., Rapelanoro, R., . . . Menard, D. (2012). Management of uncomplicated malaria in febrile under five-year-old children by community health workers in Madagascar: reliability of malaria rapid diagnostic tests. Malar J, 11, 85. doi:10.1186/1475-2875-11-85
Razzaque, A., Streatfield, P. K., & Gwatkin, D. R. (2007). Does health intervention improve socioeconomic inequalities of neonatal, infant and child mortality? Evidence from Matlab, Bangladesh. Int J Equity Health, 6, 4. doi:10.1186/1475-9276-6-4
Rescue International/Niger (2011). Health start child survival program: Child survival and health grants programs (CSHGP).
Robbins, A., & International/Bolivia, F. f. t. H. (1994). Community impact of PVO child survival project in Bolivian Altipiano. Paper presented at the Community impact of PVO child survival efforts: 1985-1994, Bangalore, Karnataka, India.

Robinson, S. J., & International/Indonesia, P. C. (1994). Innovations for Increasing Immunization Coverage. Paper presented at the Community Impact of PVO Child Survial Efforts: 1985 - 1994, Bangalore, Karnataka, India, October 2 -7, 1994.
Rowland, M., Hewitt, S., Durrani, N., Saleh, P., Bouma, M., & Sondorp, E. (1997). Sustainability of pyrethroid-impregnated bednets for malaria control in Afghan communities. Bull World Health Organ, 75(1), 23-29. doi:http://dx.doi.org/10.2458/azu_acku_pamphlet_ra644_m2_r653_1997
Roy, S. K., Jolly, S. P., Shafique, S., Fuchs, G. J., Mahmud, Z., Chakraborty, B., & Roy, S. (2007). Prevention of malnutrition among young children in rural Bangladesh by a food-health-care educational intervention: a randomized, controlled trial. Food Nutr Bull, 28(4), 375-383. doi:http://dx.doi.org/10.1177/156482650702800401
Roy, S. S., Mahapatra, R., Rath, S., Bajpai, A., Singh, V., Rath, S., . . . Prost, A. (2013). Improved neonatal survival after participatory learning and action with women's groups: a prospective study in rural eastern India. Bull World Health Organ, 91(6), 426-433B. doi:10.2471/BLT.12.105171
Ruel, M. T., Menon, P., Habicht, J. P., Loechl, C., Bergeron, G., Pelto, G., . . . Hankebo, B. (2008). Age-based preventive targeting of food assistance and behaviour change and communication for reduction of childhood undernutrition in Haiti: a cluster randomised trial. Lancet, 371(9612), 588-595. doi:10.1016/S0140-6736(08)60271-8
Rutherford, M. E., Dockerty, J. D., Jasseh, M., Howie, S. R., Herbison, P., Jeffries, D. J., . . . Hill, P. C. (2009). Preventive measures in infancy to reduce under-five mortality: a case-control study in The Gambia. Trop Med Int Health, 14(2), 149-155. doi:10.1111/j.1365-3156.2008.02204.x
Ryman, T. K., Trakroo, A., Wallace, A., Gupta, S. K., Wilkins, K., Mehta, P., & Dietz, V. (2011). Implementation and evaluation of the Reaching Every District (RED) strategy in Assam, India, 2005-2008. Vaccine, 29(14), 2555-2560. doi:10.1016/j.vaccine.2011.01.061
Ryman, T., Macauley, R., Nshimirimana, D., Taylor, P., Shimp, L., & Wilkins, K. (2010). Reaching every district (RED) approach to strengthen routine immunization services: evaluation in the African region, 2005. J Public Health (Oxf), 32(1), 18-25. doi:10.1093/pubmed/fdp048
Sadler, K., Myatt, M., Feleke, T., & Collins, S. (2007). A comparison of the programme coverage of two therapeutic feeding interventions implemented in neighbouring districts of Malawi. Public Health Nutr, 10(9), 907-913. doi:10.1017/S1368980007711035
Saleem, A. F., Mahmud, S., Baig-Ansari, N., & Zaidi, A. K. (2014). Impact of maternal education about complementary feeding on their infants' nutritional outcomes in low- and middle-income households: a community-based randomized interventional study in Karachi, Pakistan. J Health Popul Nutr, 32(4), 623-633.
SANRU III (2006). Final Evaluation.
Satti, H., McLaughlin, M. M., Omotayo, D. B., Keshavjee, S., Becerra, M. C., Mukherjee, J. S., & Seung, K. J. (2012). Outcomes of comprehensive care for children empirically treated for multidrug-resistant tuberculosis in a setting of high HIV prevalence. PLoS One, 7(5), e37114. doi:10.1371/journal.pone.0037114
Save the Children/Australia (2004). Evaluation of Sayaboury primary health care project, Phase IV: Lao People's Democratic Republic.
Save the Children/Bolivia (2008). Food security program: Assessment survey report.
Save the Children/Ethiopia (2006). Essential Services for Maternal and Child Survival in Ethiopia: Mobilizing the Traditional and Public Health Sectors and Informing Programming for Pastoralist Populations.
Save the Children/Ethiopia (2013). Innovation for Scale- Enhancing Ethiopia's health extentions package in the Southerm Nations and Nationalities People's Region (SNNPR): Report of the final evaluation.
Save the Children/Guinea (2006). Child Survival 18-Guinea final evaluation report: Community health Initiative for the districts of Kouroussa and Mandiana Guinea.

Sazawal, S., Black, R. E., Ramsan, M., Chwaya, H. M., Dutta, A., Dhingra, U., . . . Kabole, F. M. (2007). Effect of zinc supplementation on mortality in children aged 1-48 months: a community-based randomised placebo-controlled trial. Lancet, 369(9565), 927-934. doi:10.1016/S0140-6736(07)60452-8

Sazawal, S., Black, R. E., Ramsan, M., Chwaya, H. M., Stoltzfus, R. J., Dutta, A., . . . Kabole, F. M. (2006). Effects of routine prophylactic supplementation with iron and folic acid on admission to hospital and mortality in preschool children in a high malaria transmission setting: community-based, randomised, placebo-controlled trial. Lancet, 367(9505), 133-143. doi:10.1016/S0140-6736(06)67962-2

Schellenberg, J. R., Abdulla, S., Nathan, R., Mukasa, O., Marchant, T. J., Kikumbih, N., . . . Lengeler, C. (2001). Effect of large-scale social marketing of insecticide-treated nets on child survival in rural Tanzania. Lancet, 357(9264), 1241-1247. doi:10.1016/S0140-6736(00)04404-4

Schemann, J. F., Guinot, C., Traore, L., Zefack, G., Dembele, M., Diallo, I., . . . Malvy, D. (2007). Longitudinal evaluation of three azithromycin distribution strategies for treatment of trachoma in a sub-Saharan African country, Mali. Acta Trop, 101(1), 40-53. doi:10.1016/j.actatropica.2006.12.003

Schroeder, D. G., Pachon, H., Dearden, K. A., Ha, T. T., Lang, T. T., & Marsh, D. R. (2002). An integrated child nutrition intervention improved growth of younger more malnourished children in northern Viet Nam. Food and Nutrition Bulletin, vol 23 supplement 50-58

Schultink, W., Mitra, K., & Mustaphi, P. (2007). Cost-effective reduction in under nutrition in India through community based interventions.

Schumann, K., Longfils, P., Monchy, D., von Xylander, S., Weinheimer, H., & Solomons, N. W. (2009). Efficacy and safety of twice-weekly administration of three RDAs of iron and folic acid with and without complement of 14 essential micronutrients at one or two RDAs: a placebo-controlled intervention trial in anemic Cambodian infants 6 to 24 months of age. Eur J Clin Nutr, 63(3), 355-368. doi:10.1038/sj.ejcn.1602930

Sepulveda, J., Bustreo, F., Tapia, R., Rivera, J., Lozano, R., Olaiz, G., . . . Valdespino, J. L. (2006). Improvement of child survival in Mexico: the diagonal approach. Lancet, 368(9551), 2017-2027. doi:10.1016/S0140-6736(06)69569-X

Sesay, F. F., Hodges, M. H., Kamara, H. I., Turay, M., Wolfe, A., Samba, T. T., . . . Jambai, A. (2015). High coverage of vitamin A supplementation and measles vaccination during an integrated Maternal and Child Health Week in Sierra Leone. Int Health, 7(1), 26-31. doi:10.1093/inthealth/ihu073

Sesay, S., Milligan, P., Touray, E., Sowe, M., Webb, E. L., Greenwood, B. M., & Bojang, K. A. (2011). A trial of intermittent preventive treatment and home-based management of malaria in a rural area of The Gambia. Malar J, 10, 2. doi:10.1186/1475-2875-10-2

Shahid, N. S., Greenough, W. B., 3rd, Samadi, A. R., Huq, M. I., & Rahman, N. (1996). Hand washing with soap reduces diarrhoea and spread of bacterial pathogens in a Bangladesh village. J Diarrhoeal Dis Res, 14(2), 85-89.

Sharma, V. P. (1987). Community-based malaria control in India. Parasitol Today, 3(7), 222-226. doi:http://dx.doi.org/10.1016/0169-4758(87)90066-4

Shaw, B., Amouzou, A., Miller, N. P., Tsui, A. O., Bryce, J., Tafesse, M., & Surkan, P. J. (2015). Determinants of Utilization of Health Extension Workers in the Context of Scale-Up of Integrated Community Case Management of Childhood Illnesses in Ethiopia. Am J Trop Med Hyg, 93(3), 636-647. doi:10.4269/ajtmh.14-0660

Shewade, H. D., Patro, B. K., Bharti, B., Soundappan, K., Kaur, A., & Taneja, N. (2013). Effectiveness of indigenous ready-to-use therapeutic food in community-based management of uncomplicated severe acute malnutrition: a randomized controlled trial from India. J Trop Pediatr, 59(5), 393-398. doi:10.1093/tropej/fmt039

Shiff, C., Checkley, W., Winch, P., Premji, Z., Minjas, J., & Lubega, P. (1996). Changes in weight gain and anaemia attributable to malaria in Tanzanian children living under holoendemic conditions. Trans R Soc Trop Med Hyg, 90(3), 262-265. doi:http://dx.doi.org/10.1016/s0035-9203(96)90240-0

Siekmans, K., Sohani, S., Kisia, J., Kiilu, K., Wamalwa, E., Nelima, F., . . . Ngindu, A. (2013). Community case management of malaria: a pro-poor intervention in rural Kenya. Int Health, 5(3), 196-204. doi:10.1093/inthealth/iht017

Sievers, A. C., Lewey, J., Musafiri, P., Franke, M. F., Bucyibaruta, B. J., Stulac, S. N., . . . Daily, J. P. (2008). Reduced paediatric hospitalizations for malaria and febrile illness patterns following implementation of community-based malaria control programme in rural Rwanda. Malar J, 7, 167. doi:10.1186/1475-2875-7-167

Sing, K., Mathew, M., & Bhalerao, V. R. (1986). Impact of community-based immunization services. J Postgrad Med, 32(3), 131-133.

Sircar, B. K., Sengupta, P. G., Mondal, S. K., Gupta, D. N., Saha, N. C., Ghosh, S., . . . Pal, S. C. (1987). Effect of handwashing on the incidence of diarrhoea in a Calcutta slum. J Diarrhoeal Dis Res, 5(2), 112-114.

Sirima, S. B., Konate, A., Tiono, A. B., Convelbo, N., Cousens, S., & Pagnoni, F. (2003). Early treatment of childhood fevers with pre-packaged antimalarial drugs in the home reduces severe malaria morbidity in Burkina Faso. Trop Med Int Health, 8(2), 133-139. doi: http://dx.doi.org/10.1046/j.1365-3156.2003.00997.x

Sivhaga, K., Hlabano, B., & Odhiambo, P. O. (2012). Using partnership approach to reduce mortality and morbidity among children under five in Limpopo province, South Africa. Pan Afr Med J, 13 Suppl 1, 14.

Skarbinski, J., Massaga, J. J., Rowe, A. K., & Kachur, S. P. (2007). Distribution of free untreated bednets bundled with insecticide via an integrated child health campaign in Lindi Region, Tanzania: lessons for future campaigns. Am J Trop Med Hyg, 76(6), 1100-1106.

Snow, R. W., Lindsay, S. W., Hayes, R. J., & Greenwood, B. M. (1988). Permethrin-treated bed nets (mosquito nets) prevent malaria in Gambian children. Trans R Soc Trop Med Hyg, 82(6), 838-842. doi:http://dx.doi.org/10.1016/0035-9203(88)90011-9

Snow, R. W., Rowan, K. M., Lindsay, S. W., & Greenwood, B. M. (1988). A trial of bed nets (mosquito nets) as a malaria control strategy in a rural area of The Gambia, West Africa. Trans R Soc Trop Med Hyg, 82(2), 212-215. doi:http://dx.doi.org/10.1016/0035-9203(88)90414-2

Sobsey, M. D., Handzel, T., & Venczel, L. (2003). Chlorination and safe storage of household drinking water in developing countries to reduce waterborne disease. Water Sci Technol, 47(3), 221-228.

Sommer, A., Tarwotjo, I., Djunaedi, E., West, K. P., Jr., Loeden, A. A., Tilden, R., & Mele, L. (1986). Impact of vitamin A supplementation on childhood mortality. A randomised controlled community trial. Lancet, 1(8491), 1169-1173.

Soofi, S., Ahmed, S., Fox, M. P., MacLeod, W. B., Thea, D. M., Qazi, S. A., & Bhutta, Z. A. (2012). Effectiveness of community case management of severe pneumonia with oral amoxicillin in children aged 2-59 months in Matiari district, rural Pakistan: a cluster-randomised controlled trial. Lancet, 379(9817), 729-737. doi:10.1016/S0140-6736(11)61714-5

Soucat, A., Levy-Bruhl, D., De Bethune, X., Gbedonou, P., Lamarque, J. P., Bangoura, O., . . . Knippenberg, R. (1997). Affordability, cost-effectiveness and efficiency of primary health care: the Bamako Initiative experience in Benin and Guinea. Int J Health Plann Manage, 12 Suppl 1, S81-108. doi:10.1002/(SICI)1099-1751(199706)12:1+<S81::AID-HPM467>3.0.CO;2-5

Sripaipan, T., Schroeder, D. G., Marsh, D. R., Pachon, H., Dearden, K. A., Ha, T. T., & Lang, T. T. (2002). Effect of an integrated nutrition program on child morbidity due to respiratory infection and diarrhea in northen Viet Nam. Food and Nutrition Bulletin, vol 23 supplement 67-74

Stanton, B. F., & Clemens, J. D. (1987). An educational intervention for altering water-sanitation behaviors to reduce childhood diarrhea in urban Bangladesh. II. A randomized trial to assess the impact of the intervention on hygienic behaviors and rates of diarrhea. Am J Epidemiol, 125(2), 292-301. doi:http://dx.doi.org/10.1016/0277-9536(87)90054-2

Stauber, C. E., Kominek, B., Liang, K. R., Osman, M. K., & Sobsey, M. D. (2012). Evaluation of the impact of the plastic BioSand filter on health and drinking water quality in rural Tamale, Ghana. Int J Environ Res Public Health, 9(11), 3806-3823. doi:10.3390/ijerph9113806

Sternin, M., Sternin, J., & Marsh, D. L. (1999). Scaling up a poverty alleviation and nutrition program in Vietnam. In T. Marchione (Ed.), Scaling up, scaling down: Overcoming malnutrition in developing countries (pp. 97-117). Amsterdam, The Netherlands: Gordon and Breach.

Stewart, C. P., Christian, P., LeClerq, S. C., West, K. P., Jr., & Khatry, S. K. (2009). Antenatal supplementation with folic acid + iron + zinc improves linear growth and reduces peripheral adiposity in school-age children in rural Nepal. Am J Clin Nutr, 90(1), 132-140. doi:10.3945/ajcn.2008.27368

Suchdev, P. S., Ruth, L. J., Woodruff, B. A., Mbakaya, C., Mandava, U., Flores-Ayala, R., . . . Quick, R. (2012). Selling Sprinkles micronutrient powder reduces anemia, iron deficiency, and vitamin A deficiency in young children in Western Kenya: a cluster-randomized controlled trial. Am J Clin Nutr, 95(5), 1223-1230. doi:10.3945/ajcn.111.030072

Supplementation with Multiple Micronutrients Intervention Trial Study, G., Shankar, A. H., Jahari, A. B., Sebayang, S. K., Aditiawarman, Apriatni, M., . . . Sofia, G. (2008). Effect of maternal multiple micronutrient supplementation on fetal loss and infant death in Indonesia: a double-blind cluster-randomised trial. Lancet, 371(9608), 215-227. doi:10.1016/S0140-6736(08)60133-6

Sur, D., Gupta, D. N., Mondal, S. K., Ghosh, S., Manna, B., Rajendran, K., & Bhattacharya, S. K. (2003). Impact of zinc supplementation on diarrheal morbidity and growth pattern of low birth weight infants in kolkata, India: a randomized, double-blind, placebo-controlled, community-based study. Pediatrics, 112(6 Pt 1), 1327-1332. doi:http://dx.doi.org/10.1542/peds.112.6.1327

Sur, D., Manna, B., Niyogi, S. K., Ramamurthy, T., Palit, A., Nomoto, K., . . . Bhattacharya, S. K. (2011). Role of probiotic in preventing acute diarrhoea in children: a community-based, randomized, double-blind placebo-controlled field trial in an urban slum. Epidemiol Infect, 139(6), 919-926. doi:10.1017/S0950268810001780

Sutter, E., & Maphorogo, S. (1996). Integration of community-based trachoma control in primary health care in South Africa. Rev Int Trach Pathol Ocul Trop Subtrop Sante Publique, 73, 19-50.

Tandon, B. N. (1989). Nutritional interventions through primary health care: impact of the ICDS projects in India. Bull World Health Organ, 67(1), 77-80.

Taylor, W. R., Chahnazarian, A., Weinman, J., Wernette, M., Roy, J., Pebley, A. R., . . . Ma-Disu, M. (1993). Mortality and use of health services surveys in rural Zaire. Int J Epidemiol, 22 Suppl 1, S15-19. doi:http://dx.doi.org/10.1093/ije/22.supplement_1.s15

Tekce, B. (1982). Oral rehydration therapy: an assessment of mortality effects in rural Egypt. Stud Fam Plann, 13(11), 315-327. doi:http://dx.doi.org/10.2307/1965803

Thang, N. D., Erhart, A., Hung le, X., Thuan le, K., Xa, N. X., Thanh, N. N., . . . D'Alessandro, U. (2009). Rapid decrease of malaria morbidity following the introduction of community-based monitoring in a rural area of central Vietnam. Malar J, 8, 3. doi:10.1186/1475-2875-8-3

Thapa, S., Choe, M. K., & Retherford, R. D. (2005). Effects of vitamin A supplementation on child mortality: evidence from Nepal's 2001 Demographic and Health Survey. Trop Med Int Health, 10(8), 782-789. doi:10.1111/j.1365-3156.2005.01448.x

Thompson, M. E., & Harutyunyan, T. L. (2009). Impact of a community-based integrated management of childhood illnesses (IMCI) programme in Gegharkunik, Armenia. Health Policy Plan, 24(2), 101-107. doi:10.1093/heapol/czn048

Tielsch, J. M., Khatry, S. K., Stoltzfus, R. J., Katz, J., LeClerq, S. C., Adhikari, R., . . . Black, R. E. (2006). Effect of routine prophylactic supplementation with iron and folic acid on preschool child mortality in southern Nepal: community-based, cluster-randomised, placebo-controlled trial. Lancet, 367(9505), 144-152. doi:10.1016/S0140-6736(06)67963-4

Tielsch, J. M., Khatry, S. K., Stoltzfus, R. J., Katz, J., LeClerq, S. C., Adhikari, R., . . . Shresta, S. (2007). Effect of daily zinc supplementation on child mortality in southern Nepal: a community-based, cluster randomised, placebo-controlled trial. Lancet, 370(9594), 1230-1239. doi:10.1016/S0140-6736(07)61539-6

Tielsch, J. M., Rahmathullah, L., Thulasiraj, R. D., Katz, J., Coles, C., Sheeladevi, S., . . . Prakash, K. (2007). Newborn vitamin A dosing reduces the case fatality but not incidence of common childhood morbidities in South India. J Nutr, 137(11), 2470-2474.

Tine, R. C., Faye, B., Ndour, C. T., Ndiaye, J. L., Ndiaye, M., Bassene, C., . . . Gaye, O. (2011). Impact of combining intermittent preventive treatment with home management of malaria in children less than 10 years in a rural area of Senegal: a cluster randomized trial. Malar J, 10, 358. doi:10.1186/1475-2875-10-358

Tine, R. C., Ndour, C. T., Faye, B., Cairns, M., Sylla, K., Ndiaye, M., . . . Gaye, O. (2014). Feasibility, safety and effectiveness of combining home based malaria management and seasonal malaria chemoprevention in children less than 10 years in Senegal: a cluster-randomised trial. Trans R Soc Trop Med Hyg, 108(1), 13-21. doi:10.1093/trstmh/trt103

Tiono, A. B., Kabore, Y., Traore, A., Convelbo, N., Pagnoni, F., & Sirima, S. B. (2008). Implementation of Home based management of malaria in children reduces the work load for peripheral health facilities in a rural district of Burkina Faso. Malar J, 7, 201. doi:10.1186/1475-2875-7-201

Tomlinson, M., Doherty, T., Ijumba, P., Jackson, D., Lawn, J., Persson, L. A., . . . Chopra, M. (2014). Goodstart: a cluster randomised effectiveness trial of an integrated, community-based package for maternal and newborn care, with prevention of mother-to-child transmission of HIV in a South African township. Trop Med Int Health, 19(3), 256-266. doi:10.1111/tmi.12257

Tomlinson, M., Rotheram-Borus, M. J., Harwood, J., le Roux, I. M., O'Connor, M., & Worthman, C. (2015). Community health workers can improve child growth of antenatally-depressed, South African mothers: a cluster randomized controlled trial. BMC Psychiatry, 15, 225. doi:10.1186/s12888-015-0606-7

Tripathy, P., Nair, N., Barnett, S., Mahapatra, R., Borghi, J., Rath, S., . . . Costello, A. (2010). Effect of a participatory intervention with women's groups on birth outcomes and maternal depression in Jharkhand and Orissa, India: a cluster-randomised controlled trial. Lancet, 375(9721), 1182-1192. doi:10.1016/S0140-6736(09)62042-0

Tumwine, J. K., & Mackenzie, S. (1992). Child survival in a rural area in Zimbabwe: are we winning? Cent Afr J Med, 38(1), 30-36.

Turan, J. M., & Say, L. (2003). Community-based antenatal education in Istanbul, Turkey: effects on health behaviours. Health Policy Plan, 18(4), 391-398. doi:http://dx.doi.org/10.1093/heapol/czg047

UNICEF/Bihar&Jhar. (2007). Dular: An integrated community based child development and nutrition project Bihar & Jharkhand India.

UNICEF/Rajasthan. (2007). Anchal se angan tak: Community based integrated nutrition strategy, Rajasthan.

USAID/Burundi (2013). Community health systems strengthening in Cibitoke province, Burundi: Mabayi child survival project final evaluation report.

Vazir, S., Engle, P., Balakrishna, N., Griffiths, P. L., Johnson, S. L., Creed-Kanashiro, H., . . . Bentley, M. E. (2013). Cluster-randomized trial on complementary and responsive feeding education to caregivers found improved dietary intake, growth and development among rural Indian toddlers. Matern Child Nutr, 9(1), 99-117. doi:10.1111/j.1740-8709.2012.00413.x

Velema, J. P., Alihonou, E. M., Gandaho, T., & Hounye, F. H. (1991). Childhood mortality among users and non-users of primary health care in a rural west African community. Int J Epidemiol, 20(2), 474-479. doi:http://dx.doi.org/10.1093/ije/20.2.474

Vijayaraghavan, K., Radhaiah, G., Prakasam, B. S., Sarma, K. V., & Reddy, V. (1990). Effect of massive dose vitamin A on morbidity and mortality in Indian children. Lancet, 336(8727), 1342-1345. doi:http://dx.doi.org/10.1016/0140-6736(90)92895-o

Vir, S. C., Kalita, A., Mondal, S., & Malik, R. (2014). Impact of community-based mitanin programme on undernutrition in rural Chhattisgarh State, India. Food Nutr Bull, 35(1), 83-91. doi:http://dx.doi.org/10.1177/156482651403500110

Wafula, E. M., Kinyanjui, M. M., Nyabola, L., & Tenambergen, E. D. (2000). Effect of improved stoves on prevalence of acute respiration infection and conjunctivitis among children and women in a rural community in Kenya. East Afr Med J, 77(1), 37-41. doi:http://dx.doi.org/10.4314/eamj.v77i1.46379

Webster, J., Lines, J., Bruce, J., Armstrong Schellenberg, J. R., & Hanson, K. (2005). Which delivery systems reach the poor? A review of equity of coverage of ever-treated nets, never-treated nets, and immunisation to reduce child mortality in Africa. Lancet Infect Dis, 5(11), 709-717. doi:10.1016/S1473-3099(05)70269-3

West, K. P., Jr., Pokhrel, R. P., Katz, J., LeClerq, S. C., Khatry, S. K., Shrestha, S. R., . . . Sommer, A. (1991). Efficacy of vitamin A in reducing preschool child mortality in Nepal. Lancet, 338(8759), 67-71. doi:http://dx.doi.org/10.1016/0140-6736(91)90070-6

Wilford, R., Golden, K., & Walker, D. G. (2012). Cost-effectiveness of community-based management of acute malnutrition in Malawi. Health Policy Plan, 27(2), 127-137. doi:10.1093/heapol/czr017

Williamson, N. E. (1982). An attempt to reduce infant and child mortality in Bohol, Philippines. Stud Fam Plann, 13(4), 106-117. doi:http://dx.doi.org/10.2307/1965706

Wilson, J. M., & Chandler, G. N. (1993). Sustained improvements in hygiene behaviour amongst village women in Lombok, Indonesia. Trans R Soc Trop Med Hyg, 87(6), 615-616. doi:http://dx.doi.org/10.1016/0035-9203(93)90260-w

Winch, P. J., Bagayoko, A., Diawara, A., Kane, M., Thiero, F., Gilroy, K., . . . Swedberg, E. (2003). Increases in correct administration of chloroquine in the home and referral of sick children to health facilities through a community-based intervention in Bougouni District, Mali. Trans R Soc Trop Med Hyg, 97(5), 481-490. doi:http://dx.doi.org/10.1016/s0035-9203(03)80001-9

Wogi, A., Teno, D., Bulto, T., Deressa, W., Alemu, H., & Nigussie, M. (2014). Effect of integrated community case management of common childhood illnesses on the quality of malaria case management provided by health extension workers at health posts. Ethiop Med J, 52 Suppl 3, 99-108.

World Relief/Burundi (2012). Ramba Kibondo "Live Long Child" Child Survival Project: Final evaluation project, Kibuye Health District, Gitega Province, Burundi.

World Relief/Malawi (2004). Final evaluation of the Tiweko Tose child survival project.

World Relief/Rwanda (2006). Rwanda "Umucyo" (Illumination) Child Survival project: Final evaluation report.

World Renew/India (2012). FInal report on the Parivartan ("Transformation") Child Survival Project, Sahibganj district, Jarkhand state, India.

World Vision/Afghanistan (2013). Better Health for Afghan Mother and Children (BHAMC) Project: Final Evaluation project, Karukh, Zindajan, Kohsan and Chisht-e-Sharif districts, Herat province, Afghanistan.

World Vision/India (2008). Pragati Child Survival Project, Uttar Pradesh, India: Final Evaluation.

Wuehler, S. E., Sempertegui, F., & Brown, K. H. (2008). Dose-response trial of prophylactic zinc supplements, with or without copper, in young Ecuadorian children at risk of zinc deficiency. Am J Clin Nutr, 87(3), 723-733.

Yach, D., Hoogendoorn, L., & Von Schirnding, Y. E. (1987). Village health workers are able to teach mothers how to safely prepare sugar/salt solutions. Paediatr Perinat Epidemiol, 1(2), 153-161. doi:http://dx.doi.org/10.1111/j.1365-3016.1987.tb00105.x

Yansaneh, A. I., Moulton, L. H., George, A. S., Rao, S. R., Kennedy, N., Bangura, P., . . . Diaz, T. (2014). Influence of community health volunteers on care seeking and treatment coverage for common childhood illnesses in the context of free health care in rural Sierra Leone. Trop Med Int Health, 19(12), 1466-1476. doi:10.1111/tmi.12383

Yeboah-Antwi, K., Pilingana, P., Macleod, W. B., Semrau, K., Siazeele, K., Kalesha, P., . . . Hamer, D. H. (2010). Community case management of fever due to malaria and pneumonia in children under five in Zambia: a cluster randomized controlled trial. PLoS Med, 7(9), e1000340. doi:10.1371/journal.pmed.1000340

Zeba, A. N., Sorgho, H., Rouamba, N., Zongo, I., Rouamba, J., Guiguemde, R. T., . . . Ouedraogo, J. B. (2008). Major reduction of malaria morbidity with combined vitamin A and zinc supplementation in young children in Burkina Faso: a randomized double blind trial. Nutr J, 7, 7. doi:10.1186/1475-2891-7-7

Appendix S2.
References for Assessments Cited in the Child Health Analysis

S1. Perry, H., Cayemittes, M., Philippe, F., Dowell, D., Dortonne, J. R., Menager, H., . . . Berggren, G. (2006). Reducing under-five mortality through Hopital Albert Schweitzer's integrated system in Haiti. Health Policy Plan, 21(3), 217-230. doi:10.1093/heapol/czl005

S2. Mtango, F. D., & Neuvians, D. (1986). Acute respiratory infections in children under five years. Control project in Bagamoyo District, Tanzania. Trans R Soc Trop Med Hyg, 80(6), 851-858. doi:http://dx.doi.org/10.1016/0035-9203(86)90241-5

S3. Pence, B. W., Nyarko, P., Phillips, J. F., & Debpuur, C. (2007). The effect of community nurses and health volunteers on child mortality: the Navrongo Community Health and Family Planning Project. Scand J Public Health, 35(6), 599-608. doi:10.1080/14034940701349225

S4. Pandey, M. R., Daulaire, N. M., Starbuck, E. S., Houston, R. M., & McPherson, K. (1991). Reduction in total under-five mortality in western Nepal through community-based antimicrobial treatment of pneumonia. Lancet, 338(8773), 993-997. doi:http://dx.doi.org/10.1016/0140-6736(91)91847-n

S5. Bari A, Sadruddin S, Khan A, Khan IH, Khan A, Lehri IA, et.al. Community case management of severe pneumonia with oral Amoxicillin in children aged 2-59 months in Haripur district, Pakistan:a cluster randomized trial. Lancet, 2011;378: 1796-803.

S6. Pandey, M. R., Sharma, P. R., Gubhaju, B. B., Shakya, G. M., Neupane, R. P., Gautam, A., & Shrestha, I. B. (1989). Impact of a pilot acute respiratory infection (ARI) control programme in a rural community of the hill region of Nepal. Ann Trop Paediatr, 9(4), 212-220. doi:http://dx.doi.org/10.1080/02724936.1989.11748635

S7. Bang, A. T., Bang, R. A., & Sontakke, P. G. (1994). Management of childhood pneumonia by traditional birth attendants. The SEARCH Team. Bull World Health Organ, 72(6), 897-905.

S8. Khan, A. J., Khan, J. A., Akbar, M., & Addiss, D. G. (1990). Acute respiratory infections in children: a case management intervention in Abbottabad District, Pakistan. Bull World Health Organ, 68(5), 577-585.

S9. Fauveau, V., Stewart, M. K., Chakraborty, J., & Khan, S. A. (1992). Impact on mortality of a community-based programme to control acute lower respiratory tract infections. Bull World Health Organ, 70(1), 109-116.

S10. Ali, M., Emch, M., Tofail, F., & Baqui, A. H. (2001). Implications of health care provision on acute lower respiratory infection mortality in Bangladeshi children. Soc Sci Med, 52(2), 267-277. doi:http://dx.doi.org/10.1016/s0277-9536(00)00120-9

S11. Agarwal, D. K., Bhatia, B. D., & Agarwal, K. N. (1993). Simple approach to acute respiratory infection in rural under five children. Indian Pediatr, 30(5), 629-635.

S12. Lye, M. S., Nair, R. C., Choo, K. E., Kaur, H., & Lai, K. P. (1996). Acute respiratory tract infection: a community-based intervention study in Malaysia. J Trop Pediatr, 42(3), 138-143. doi:http://dx.doi.org/10.1093/tropej/42.3.138

S13. Mazumder, S., Taneja, S., Bahl, R., Mohan, P., Strand, T. A., Sommerfelt, H., . . . Childhood Illness Evaluation Study, G. (2014). Effect of implementation of integrated management of neonatal and childhood illness programme on treatment seeking practices for morbidities in infants: cluster randomised trial. BMJ, 349, g4988. doi:10.1136/bmj.g4988

S14. Daulaire, N. M., Starbuck, E. S., Houston, R. M., Church, M. S., Stukel, T. A., & Pandey, M. R. (1992). Childhood mortality after a high dose of vitamin A in a high risk population. BMJ, 304(6821), 207-210. doi:http://dx.doi.org/10.1136/bmj.304.6821.207

S15. Brooks, W. A., Santosham, M., Naheed, A., Goswami, D., Wahed, M. A., Diener-West, M., . . . Black, R. E. (2005). Effect of weekly zinc supplements on incidence of pneumonia and diarrhoea in children younger than 2 years in an urban, low-income population in

Bangladesh: randomised controlled trial. Lancet, 366(9490), 999-1004. doi:10.1016/S0140-6736(05)67109-7
S16. Luby, S. P., Agboatwalla, M., Feikin, D. R., Painter, J., Billhimer, W., Altaf, A., & Hoekstra, R. M. (2005). Effect of handwashing on child health: a randomised controlled trial. Lancet, 366(9481), 225-233. doi:10.1016/S0140-6736(05)66912-7
S17. Mazumder, S., Taneja, S., Bhandari, N., Dube, B., Agarwal, R. C., Mahalanabis, D., . . . Black, R. E. (2010). Effectiveness of zinc supplementation plus oral rehydration salts for diarrhoea in infants aged less than 6 months in Haryana state, India. Bull World Health Organ, 88(10), 754-760. doi:10.2471/BLT.10.075986
S18. Sripaipan, T., Schroeder, D. G., Marsh, D. R., Pachon, H., Dearden, K. A., Ha, T. T., & Lang, T. T. (2002). Effect of an integrated nutrition program on child morbidity due to respiratory infection and diarrhea in northen Viet Nam. Food and Nutrition Bulletin, vol 23 supplement 67-74
S19. Bhandari, N., Mazumder, S., Taneja, S., Dube, B., Agarwal, R. C., Mahalanabis, D., . . . Bhan, M. K. (2008). Effectiveness of zinc supplementation plus oral rehydration salts compared with oral rehydration salts alone as a treatment for acute diarrhea in a primary care setting: a cluster randomized trial. Pediatrics, 121(5), e1279-1285. doi:10.1542/peds.2007-1939
S20. Bhandari, N., Bahl, R., Taneja, S., Strand, T., Molbak, K., Ulvik, R. J., . . . Bhan, M. K. (2002). Effect of routine zinc supplementation on pneumonia in children aged 6 months to 3 years: randomised controlled trial in an urban slum. BMJ, 324(7350), 1358. doi:http://dx.doi.org/10.1136/bmj.324.7350.1358
S21. Baqui, A. H., Black, R. E., El Arifeen, S., Yunus, M., Chakraborty, J., Ahmed, S., & Vaughan, J. P. (2002). Effect of zinc supplementation started during diarrhoea on morbidity and mortality in Bangladeshi children: community randomised trial. BMJ, 325(7372), 1059. doi:http://dx.doi.org/10.1136/bmj.325.7372.1059
S22. Sur, D., Gupta, D. N., Mondal, S. K., Ghosh, S., Manna, B., Rajendran, K., & Bhattacharya, S. K. (2003). Impact of zinc supplementation on diarrheal morbidity and growth pattern of low birth weight infants in kolkata, India: a randomized, double-blind, placebo-controlled, community-based study. Pediatrics, 112(6 Pt 1), 1327-1332. doi:http://dx.doi.org/10.1542/peds.112.6.1327
S23. Gupta, D. N., Rajendran, K., Mondal, S. K., Ghosh, S., & Bhattacharya, S. K. (2007). Operational feasibility of implementing community-based zinc supplementation: impact on childhood diarrheal morbidity. Pediatr Infect Dis J, 26(4), 306-310. doi:10.1097/01.inf.0000258692.65485.d9
S24. Penny, M. E., Peerson, J. M., Marin, R. M., Duran, A., Lanata, C. F., Lonnerdal, B., . . . Brown, K. H. (1999). Randomized, community-based trial of the effect of zinc supplementation, with and without other micronutrients, on the duration of persistent childhood diarrhea in Lima, Peru. J Pediatr, 135(2 Pt 1), 208-217. doi:http://dx.doi.org/10.1016/s0022-3476(99)70024-7
S25. Haggerty, P. A., Muladi, K., Kirkwood, B. R., Ashworth, A., & Manunebo, M. (1994). Community-based hygiene education to reduce diarrhoeal disease in rural Zaire: impact of the intervention on diarrhoeal morbidity. Int J Epidemiol, 23(5), 1050-1059. doi:http://dx.doi.org/10.1093/ije/23.5.1050

S26. Roy, S. K., Jolly, S. P., Shafique, S., Fuchs, G. J., Mahmud, Z., Chakraborty, B., & Roy, S. (2007). Prevention of malnutrition among young children in rural Bangladesh by a food-health-care educational intervention: a randomized, controlled trial. Food Nutr Bull, 28(4), 375-383. doi:http://dx.doi.org/10.1177/156482650702800401
S27. Arifeen, S. E., Blum, L. S., Hoque, D. M., Chowdhury, E. K., Khan, R., Black, R. E., . . . Bryce, J. (2004). Integrated Management of Childhood Illness (IMCI) in Bangladesh: early findings

from a cluster-randomised study. Lancet, 364(9445), 1595-1602. doi:10.1016/S0140-6736(04)17312-1

S28. Stanton, B. F., & Clemens, J. D. (1987). An educational intervention for altering water-sanitation behaviors to reduce childhood diarrhea in urban Bangladesh. II. A randomized trial to assess the impact of the intervention on hygienic behaviors and rates of diarrhea. Am J Epidemiol, 125(2), 292-301. doi:http://dx.doi.org/10.1016/0277-9536(87)90054-2

S29. Khan, M. U. (1982). Interruption of shigellosis by hand washing. Trans R Soc Trop Med Hyg, 76(2), 164-168. doi:http://dx.doi.org/10.1016/0035-9203(82)90266-8

S30. Han, A. M., & Hlaing, T. (1989). Prevention of diarrhoea and dysentery by hand washing. Trans R Soc Trop Med Hyg, 83, 128-131. doi:http://dx.doi.org/10.1016/0035-9203(89)90737-2

S31. Shahid, N. S., Greenough, W. B., 3rd, Samadi, A. R., Huq, M. I., & Rahman, N. (1996). Hand washing with soap reduces diarrhoea and spread of bacterial pathogens in a Bangladesh village. J Diarrhoeal Dis Res, 14(2), 85-89.

S32. Luby, S. P., Agboatwalla, M., Painter, J., Altaf, A., Billhimer, W. L., & Hoekstra, R. M. (2004). Effect of intensive handwashing promotion on childhood diarrhea in high-risk communities in Pakistan: a randomized controlled trial. JAMA, 291(21), 2547-2554. doi:10.1001/jama.291.21.2547

S33. Alderman, H., Ndiaye, B., Linnemayr, S., Ka, A., Rokx, C., Dieng, K., & Mulder-Sibanda, M. (2008). Effectiveness of a community-based intervention to improve nutrition in young children in Senegal: a difference in difference analysis. Public Health Nutr, 12(5), 667-673. doi:10.1017/S1368980008002619

S34. Yach, D., Hoogendoorn, L., & Von Schirnding, Y. E. (1987). Village health workers are able to teach mothers how to safely prepare sugar/salt solutions. Paediatr Perinat Epidemiol, 1(2), 153-161. doi:http://dx.doi.org/10.1111/j.1365-3016.1987.tb00105.x

S35. Naheed, A., Walker Fischer, C. L., Mondal, D., Ahmed, S., Arifeen, S. E., Yunus, M., . . . Baqui, A. H. (2009). Zinc therapy for diarrhoea improves growth among Bangladeshi infants 6 to 11 months of age. J Pediatr Gastroenterol Nutr, 48(1), 89-93. doi:10.1097/MPG.0b013e31817f0182

S36. Sobsey, M. D., Handzel, T., & Venczel, L. (2003). Chlorination and safe storage of household drinking water in developing countries to reduce waterborne disease. Water Sci Technol, 47(3), 221-228.

S37. Quick, R. E., Kimura, A., Thevos, A., Tembo, M., Shamputa, I., Hutwagner, L., & Mintz, E. (2002). Diarrhea prevention through household-level water disinfection and safe storage in Zambia. Am J Trop Med Hyg, 66(5), 584-589.

S38. Quick, R. E., Venczel, L. V., Mintz, E. D., Soleto, L., Aparicio, J., Gironaz, M., . . . Tauxe, R. V. (1999). Diarrhoea prevention in Bolivia through point-of-use water treatment and safe storage: a promising new strategy. Epidemiol Infect, 122(1), 83-90. doi:http://dx.doi.org/10.1017/s0950268898001782

S39. McGuigan, K.G., Samaiyar, P., du Preez, M., Conroy, R.M. High Compliance Randomized Controlled Field Trial of Solar Disinfection of Drinking Water and Its Impact on Childhood Diarrhea in Rural Cambodia. Environ. Sci. Technol. 2011(45): 7862-7867

S40. du Preez, M., Conroy, R.M., Ligondo, S., Hennessy, J., Elmore-Meegan, M., Soita, A., McGuigan, K.G. Randomized Intervention Study of Solar Disinfection of Drinking Water in the Prevention of Dysentery in Kenyan Children Aged under 5 Years. Environ. Sci. Technol. 2011(45): 9315 -9323

S41. Conroy, R. M., Meegan, M. E., Joyce, T., McGuigan, K., & Barnes, J. (1999). Solar disinfection of water reduces diarrhoeal disease: an update. Arch Dis Child, 81(4), 337-338. doi:http://dx.doi.org/10.1136/adc.81.4.337

S42. Peletz R, Simunyama M, Sarenje K, Baisley K, Filteau S, Kelly P, Clasen T. Assessing water filtration and safe storage in households with young children of HIV positive mothers: a randomized controlled trial in Zambia. PLoS ONE 7(10): e46548

S43. Stauber, C. E., Kominek, B., Liang, K. R., Osman, M. K., & Sobsey, M. D. (2012). Evaluation of the impact of the plastic BioSand filter on health and drinking water quality in rural Tamale, Ghana. International journal of environmental research and public health, 9(11), 3806-3823

S44. D'Alessandro, U., Olaleye, B. O., McGuire, W., Langerock, P., Bennett, S., Aikins, M. K., . . . Greenwood, B. M. (1995). Mortality and morbidity from malaria in Gambian children after introduction of an impregnated bednet programme. Lancet, 345(8948), 479-483. doi:http://dx.doi.org/10.1016/s0140-6736(95)90582-0

S45. Binka, F. N., Kubaje, A., Adjuik, M., Williams, L. A., Lengeler, C., Maude, G. H., . . . Smith, P. G. (1996). Impact of permethrin impregnated bednets on child mortality in Kassena-Nankana district, Ghana: a randomized controlled trial. Trop Med Int Health, 1(2), 147-154. doi:http://dx.doi.org/10.1111/j.1365-3156.1996.tb00020.x

S46. Nevill, C. G., Some, E. S., Mung'ala, V. O., Mutemi, W., New, L., Marsh, K., . . . Snow, R. W. (1996). Insecticide-treated bednets reduce mortality and severe morbidity from malaria among children on the Kenyan coast. Trop Med Int Health, 1(2), 139-146. doi:http://dx.doi.org/10.1111/j.1365-3156.1996.tb00019.x

S47. Alonso, P. L., Lindsay, S. W., Armstrong, J. R., Conteh, M., Hill, A. G., David, P. H., . . . et al. (1991). The effect of insecticide-treated bed nets on mortality of Gambian children. Lancet, 337(8756), 1499-1502. doi:http://dx.doi.org/10.1016/0140-6736(91)93194-e

S48. Phillips-Howard, P. A., Nahlen, B. L., Alaii, J. A., ter Kuile, F. O., Gimnig, J. E., Terlouw, D. J., . . . Hawley, W. A. (2003). The efficacy of permethrin-treated bed nets on child mortality and morbidity in western Kenya I. Development of infrastructure and description of study site. Am J Trop Med Hyg, 68(4 Suppl), 3-9

S49. Magbity, E. B., Marbiah, N. T., Maude, G., Curtis, C. F., Bradley, D. J., Greenwood, B. M., . . . Lines, J. D. (1997). Effects of community-wide use of lambdacyhalothrin-impregnated bednets on malaria vectors in rural Sierra Leone. Med Vet Entomol, 11(1), 79-86. doi:http://dx.doi.org/10.1111/j.1365-2915.1997.tb00293.x

S50. Deribew, A., Birhanu, Z., Sena, L., Dejene, T., Reda, A.A., Sudhakar, M., Alemseged, F. et. al. (2012). The effect of household heads training about the use of treated bed nets on the burden of malaria and anemia in under-five children: a cluster randomized controlled trial in Ethiopia. Malaria Journal, 11:8

S51. Snow, R. W., Rowan, K. M., Lindsay, S. W., & Greenwood, B. M. (1988). A trial of bed nets (mosquito nets) as a malaria control strategy in a rural area of The Gambia, West Africa. Trans R Soc Trop Med Hyg, 82(2), 212-215. doi:http://dx.doi.org/10.1016/0035-9203(88)90414-2

S52. Keating, J., Hutchinson, P., Miller, J.M., Bennett, A., Larsen, D.A., Hamainza, B., Changufu, C., Shiliya, N. & Eisele, T.P. (2012). A quasi-experimental evaluation of an interpersonal communication intervention to increase insecticide-treated net use among children in Zambia. Malaria Journal, 11:313

S53. Deribew, A., Birhanu, Z., Sena, L., Dejene, T., Reda, A.A., Sudhakar, M., Alemseged, F. et. al. (2012).The effect of household heads training on long lasting insecticide-treated bed nets utilization: a cluster randomized controlled trial in Ethiopia. Malaria Journal, 11:99

S54. Das, A., Friedman, J., Kandpal, E., Ramana, G.N.V., Gupta, R.K.D., Pradhan, M.M., Govindaraj, R. (2015). Strengthening malaria service delivery through support supervision and community mobilization in an endemic Indian setting: an evaluation of nested delivery models. Malaria Journal, 13:482

S55. Kidane, G., & Morrow, R. H. (2000). Teaching mothers to provide home treatment of malaria in Tigray, Ethiopia: a randomised trial. Lancet, 356(9229), 550-555. doi:10.1016/S0140-6736(00)02580-0

S56. Mubi, M., Janson, A., Warsame, M., Martensson, A., Kallander, K., Petzold, M.G., Ngasala, B., Maganga, G., Gustafsson, L.L., Massele, A., Tomson, G., Premji, Z., Bjorkman, A. (2011). Malaria Rapid Testing by Community Health Workers Is Effective and Safe for Targeting Malaria Treatment: Randomised Cross-Over Trial in Tanzania. PlosOne. (6) 7: e19753
S57. Yeboah-Antwi, K., Pilingana, P., Macleod, W. B., Semrau, K., Siazeele, K., Kalesha, P., & Hamer, D. H. (2010). Community case management of fever due to malaria and pneumonia in children under five in Zambia: a cluster randomized controlled trial. PLoS medicine, 7(9), e1000340
S58. Kalyango, J., Alfven, T., Peterson, S., Mugenyi, K., Karamagi, C., Rutebemberwa. (2013). Integrated community case management of malaria and pneumonia increases prompt and appropriate treatment for pneumonia symptoms in children under five years in Eastern Uganda. Malar J 12.1: 340
S59. Tinea, R.C.K., Ndourb, C.T., Fayea, B., Cairnsc, M., Syllaa, K., Ndiayea, M., Ndiayea, J.L., Sowa, D., Cissea, B., Magnussend, P., Bygbjergd, I.C., & Gayea, O. (2014). Feasibility, safety and effectiveness of combining home based malaria management and seasonal malaria chemoprevention in children less than 10 years in Senegal: a cluster-randomised trial. Trans R Soc Trop Med Hyg 2014; 108: 13-21
S60. Tine, R.C.K., Faye, B., Ndour, C.T., Ndiaye, J.L., Ndiaye, M., Bassene, C., Magnussen, P., Bygbjerg, I.C. et.al. (2011). Impact of combining intermittent preventive treatment with home management of malaria in children less than 10 years in rural area of Senegal: a cluster randomized trial. Malaria Journal, 2011; 10: 358
S61. Mockenhaupt, F. P., Reither, K., Zanger, P., Roepcke, F., Danquah, I., Saad, E., . . . Bienzle, U. (2007). Intermittent preventive treatment in infants as a means of malaria control: a randomized, double-blind, placebo-controlled trial in northern Ghana. Antimicrob Agents Chemother, 51(9), 3273-3281. doi:10.1128/AAC.00513-07
S62. Fraser-Hurt, N., Felger, I., Edoh, D., Steiger, S., Mashaka, M., Masanja, H., . . . Beck, H. P. (1999). Effect of insecticide-treated bed nets on haemoglobin values, prevalence and multiplicity of infection with Plasmodium falciparum in a randomized controlled trial in Tanzania. Trans R Soc Trop Med Hyg, 93 Suppl 1, 47-51. doi:http://dx.doi.org/10.1016/s0035-9203(99)90327-9
S63. Deribew, A., Birhanu, Z., Sena, L., Dejene, T., Reda, A.A., Sudhakar, M., Alemseged, F. et. al. (2012). The effect of household heads training on long lasting insecticide-treated bed nets utilization: a cluster randomized controlled trial in Ethiopia. Malaria Journal, 11:99
S64. Rowland, M., Hewitt, S., Durrani, N., Saleh, P., Bouma, M., & Sondorp, E. (1997). Sustainability of pyrethroid-impregnated bednets for malaria control in Afghan communities. Bull World Health Organ, 75(1), 23-29. doi:http://dx.doi.org/10.2458/azu_acku_pamphlet_ra644_m2_r653_1997
S65. Snow, R. W., Lindsay, S. W., Hayes, R. J., & Greenwood, B. M. (1988). Permethrin-treated bed nets (mosquito nets) prevent malaria in Gambian children. Trans R Soc Trop Med Hyg, 82(6), 838-842. doi:http://dx.doi.org/10.1016/0035-9203(88)90011-9
S66. Schellenberg, J. R., Abdulla, S., Nathan, R., Mukasa, O., Marchant, T. J., Kikumbih, N., . . . Lengeler, C. (2001). Effect of large-scale social marketing of insecticide-treated nets on child survival in rural Tanzania. Lancet, 357(9264), 1241-1247. doi:10.1016/S0140-6736(00)04404-4
S67. Hung le, Q., Vries, P. J., Giao, P. T., Nam, N. V., Binh, T. Q., Chong, M. T., . . . Kager, P. A. (2002). Control of malaria: a successful experience from Viet Nam. Bull World Health Organ, 80(8), 660-666
S68. Houeto, D., & Deccache, A. (2007). Child malaria in sub-saharan Africa: effective control and prevention require a health promotion approach. Int Q Community Health Educ, 28(1), 51-62. doi:10.2190/IQ.28.1.e

S69. Grabowsky, M., Farrell, N., Hawley, W., Chimumbwa, J., Hoyer, S., Wolkon, A., & Selanikio, J. (2005). Integrating insecticide-treated bednets into a measles vaccination campaign achieves high, rapid and equitable coverage with direct and voucher-based methods. Trop Med Int Health, 10(11), 1151-1160. doi:10.1111/j.1365-3156.2005.01502.x
S70. Skarbinski, J., Massaga, J. J., Rowe, A. K., & Kachur, S. P. (2007). Distribution of free untreated bednets bundled with insecticide via an integrated child health campaign in Lindi Region, Tanzania: lessons for future campaigns. Am J Trop Med Hyg, 76(6), 1100-1106.
S71. Fegan, G. W., Noor, A. M., Akhwale, W. S., Cousens, S., & Snow, R. W. (2007). Effect of expanded insecticide-treated bednet coverage on child survival in rural Kenya: a longitudinal study. Lancet, 370(9592), 1035-1039. doi:10.1016/S0140-6736(07)61477-9
S72. Houeto, D., & Deccache, A. (2007). Child malaria in sub-saharan Africa: effective control and prevention require a health promotion approach. Int Q Community Health Educ, 28(1), 51-62. doi:10.2190/IQ.28.1.e
S73. Shiff, C., Checkley, W., Winch, P., Premji, Z., Minjas, J., & Lubega, P. (1996). Changes in weight gain and anaemia attributable to malaria in Tanzanian children living under holoendemic conditions. Trans R Soc Trop Med Hyg, 90(3), 262-265. doi:http://dx.doi.org/10.1016/s0035-9203(96)90240-0
S74. Otten, M., Aregawi, M., Were, W., Karema, C., Medin, A., Bekele, W., . . . Grabowsky, M. (2009). Initial evidence of reduction of malaria cases and deaths in Rwanda and Ethiopia due to rapid scale-up of malaria prevention and treatment. Malar J, 8, 14. doi:10.1186/1475-2875-8-14
S75. Sievers, A. C., Lewey, J., Musafiri, P., Franke, M. F., Bucyibaruta, B. J., Stulac, S. N., . . . Daily, J. P. (2008). Reduced paediatric hospitalizations for malaria and febrile illness patterns following implementation of community-based malaria control programme in rural Rwanda. Malar J, 7, 167. doi:10.1186/1475-2875-7-167
S76. Diallo, D. A., Cousens, S. N., Cuzin-Ouattara, N., Nebie, I., Ilboudo-Sanogo, E., & Esposito, F. (2004). Child mortality in a West African population protected with insecticide-treated curtains for a period of up to 6 years. Bull World Health Organ, 82(2), 85-91.
S77. Habluetzel, A., Diallo, D. A., Esposito, F., Lamizana, L., Pagnoni, F., Lengeler, C., . . . Cousens, S. N. (1997). Do insecticide-treated curtains reduce all-cause child mortality in Burkina Faso? Trop Med Int Health, 2(9), 855-862. doi:http://dx.doi.org/10.1046/j.1365-3156.1997.d01-413.x
S78. Fraser-Hurt, N., & Lyimo, E. O. (1998). Insecticide-treated nets and treatment service: a trial using public and private sector channels in rural United Republic of Tanzania. Bull World Health Organ, 76(6), 607-615.
S79. Kachur, S. P., Phillips-Howard, P. A., Odhacha, A. M., Ruebush, T. K., Oloo, A. J., & Nahlen, B. L. (1999). Maintenance and sustained use of insecticide-treated bednets and curtains three years after a controlled trial in western Kenya. Trop Med Int Health, 4(11), 728-735. doi:http://dx.doi.org/10.1046/j.1365-3156.1999.00481.x
S80. Kroeger, A., Meyer, R., Mancheno, M., Gonzalez, M., & Pesse, K. (1997). Operational aspects of bednet impregnation for community-based malaria control in Nicaragua, Ecuador, Peru and Colombia. Trop Med Int Health, 2(6), 589-602. doi:http://dx.doi.org/10.1046/j.1365-3156.1997.d01-319.x
S81. Lengeler, C., Armstrong-Schellenberg, J., D'Alessandro, U., Binka, F., & Cattani, J. (1998). Relative versus absolute risk of dying reduction after using insecticide-treated nets for malaria control in Africa. Trop Med Int Health, 3(4), 286-290. doi:http://dx.doi.org/10.1046/j.1365-3156.1998.00236.x
S82. du Preez, M., Conroy, R. M., Ligondo, S., Hennessy, J., Elmore-Meegan, M., Soita, A., & McGuigan, K. G. (2011). Randomized intervention study of solar disinfection of drinking water in the prevention of dysentery in Kenyan children aged under 5 years. Environ Sci Technol, 45(21), 9315-9323. doi:10.1021/es2018835

S83. Greer, G., Akinpelumi, A., Madueke, L., Plowman, B., Fapohunda, B., Tawfik, Y., . . . Lennox, B. (2004). Improving management of chilhood malaria in Nigeria and Uganda by improving practices of patent medicine vendors. BASICS II/USAID
S84. Thang, N. D., Erhart, A., Hung le, X., Thuan le, K., Xa, N. X., Thanh, N. N., . . . D'Alessandro, U. (2009). Rapid decrease of malaria morbidity following the introduction of community-based monitoring in a rural area of central Vietnam. Malar J, 8, 3. doi:10.1186/1475-2875-8-3
S85. Mukanga, D., Babirye, R., Peterson, S., Pariyo, G.W., Ojiambo, G., Tibenderana, J.K., Nsubuga, P. & Kallander, K. (2011). Can lay community health workers be trained to use diagnostics to distinguish and treat malaria and pneumonia in children? Lessons from rural Uganda. Tropical Medicine and International Health. 2011(16)10: 1324-1242
S86. Ngasala, B.E., Malmberg, M., Carlsson, A.M., Ferreira, P.E., Petzold, M.G., Blessborn, D., Bergqvist, Y., Gil1, J.P., Premji, Z., Mårtensson, A. (2011). Effectiveness of artemether-lumefantrine provided by community health workers in underfive children with uncomplicated malaria in rural Tanzania: an open label prospective study. Malaria Journal 2011, 10:64
S87. Ratsimbasoa, A., Ravony, H., Vonimpaisomihanta, J., Raherinjafy, R., Jahevitra, M., Rapelanoro, R., Rakotomanga, J.D.M., Malvy, D., Millet, P., & Me´nard, D. (2012). Compliance, Safety, and Effectiveness of Fixed-Dose Artesunate-Amodiaquine for Presumptive Treatment of Non-Severe Malaria in the Context of Home Management of Malaria in Madagascar. Am. J. Trop. Med. Hyg., 86(2), 2012: 203-210
S88. Kisia, J., Nelima, F., Otieno, D.O., Kiilu, K., Emmanuel, W., Sohai, S., Siekmans, K., Nyandigisi, A., Akhwale, W. Factors associated with utilization of community health workesr in improving access to malaria treatment among children in Kenya. Malaria Journal. 2012. 11:248, 1-7. Retrieved from <http://www.malariajournal.com/content/11/1/248>
S89. Littrell, M., Moukam, L.V., Libite, R., Youmba, J.C., & Baugh, G. (2013). Narrowing the treatment gap with equitable access: mid-term outcomes of a community case management program in Cameroon. Health policy and planning 28.7 (2013): 705-716.
S90. Kouyate, B., Some, F., Jahn, A., Coulibaly, B., Eriksen, J., Sauerborn, R., . . . Mueller, O. (2008). Process and effects of a community intervention on malaria in rural Burkina Faso: randomized controlled trial. Malar J, 7, 50. doi:10.1186/1475-2875-7-50
S91. Ohnmar, Tun-Min, San-Shwe, Chongsuvivatwong, V. Effects of malaria volunteer training on coverage and timeliness of diagnosis: a cluster randomized controlled trial in Myanmar. Malaria Journal 2012; 11: 309
S92. Yeboah-Antwi, K., Pilingana, P., Macleod, W. B., Semrau, K., Siazeele, K., Kalesha, P., & Hamer, D. H. (2010). Community case management of fever due to malaria and pneumonia in children under five in Zambia: a cluster randomized controlled trial. PLoS medicine, 7(9), e1000340
S93. Johnson, A., Thomson, D., Atwood, S., Alley, I., Beckerman, J., Kone, I., Diakite, D., Diallo, H., Traore, B., Traore, K., Farmer, P., Murray, M., Mukherjee, J. (2013). Assessing Early Access to Care and Child Survival during a Health System Strengthening Intervention in Mali : A Repeated Cross Sectional Survey. PloS One. December 2013, Volume 8, Issue 12, e81304
S94. Lemma, H., Byass, P., Desta, A., Bosman, A., Costanzo, G., Toma, L., Fottrell, E., Marrast, A., Ambachew, Y., Getachew, A., Mulure, N., Morrone, A., Bianchi, A., Ab Barnabas, G. (2010). Deploying artemether-lumefantrine with rapid testing in Ethiopian communities: impact on malaria morbidity, mortality and healthcare resources. Tropial Medicine and International Health, volume 15 no 2 pp 241-250
S95. Nonvignon, J. Chinbuah, M.A., Gyapong, M., Abbey, M., Awini, E., Gyapong, J.O., and Aikins, M. (2012). Is home management of fevers a cost-effective way of reducing under-five mortality in Africa? The case of a rural Ghanaian District. Tropical Medicine and International Health: Volume 17, No. 8 pp. 951-957

S96. Pasha, O., Del Rosso, J., Mukaka, M., & Marsh, D. (2003). The effect of providing fansidar (sulfadoxine-pyrimethamine) in schools on mortality in school-age children in Malawi. Lancet, 361(9357), 577-578. doi:http://dx.doi.org/10.1016/s0140-6736(03)12511-1
S97. Greer, G., Akinpelumi, A., Madueke, L., Plowman, B., Fapohunda, B., Tawfik, Y., . . . Lennox, B. (2004). Improving management of chilhood malaria in Nigeria and Uganda by improving practices of patent medicine vendors. BASICS II/USAID
S98. Minnesota International Health Volunteers/Uganda (2004). Improving malaria case management in Uganda communities: Lessons from the field.
S99. Sesay, S., Milligan, P., Touray, E., Sowe, M., Webb, E.L., Greenwood, B.M., Bojang, K.A. (2011). A trial of intermittent preventative treatment and home-based management of malaria in a rural area of The Gambia. Malaria Journal. 2011, 10:2.
S100. Bojang, K.A., Akor, F., Conteh, L., Web, E., Bittaye, O., Conway, D.J., Jasseh, M., Wiseman, V., Milligan, P.J., Greenwood, B. (2011). Two Strategies for the Delivery of IPTc in an Area of Seasonal Malaria Transmission in The Gambia: A Randomised Controlled Trial. PLoS Medicine. February 2011(8) 2; e1000409
S101. Kalyango, J., Alfven, T., Peterson, S., Mugenyi, K., Karamagi, C., Rutebemberwa. (2013). Integrated community case management of malaria and pneumonia increases prompt and appropriate treatment for pneumonia symptoms in children under five years in Eastern Uganda. Malar J 12.1: 340
S102. Cisse, B., Cairns, M., Faye, E., NDiaye, O., Faye, B., et al. (2009) Randomized Trial of Piperaquine with Sulfadoxine-Pyrimethamine or Dihydroartemisinin for Malaria Intermittent Preventive Treatment in Children. PLoS ONE 4(9): e7164. doi:10.1371/journal.pone.0007164
S103. Pagnoni, F., Convelbo, N., Tiendrebeogo, J., Cousens, S., & Esposito, F. (1997). A community-based programme to provide prompt and adequate treatment of presumptive malaria in children. Trans R Soc Trop Med Hyg, 91(5), 512-517. doi:http://dx.doi.org/10.1016/s0035-9203(97)90006-7
S104. Ajayi, I. O., Browne, E. N., Garshong, B., Bateganya, F., Yusuf, B., Agyei-Baffour, P., . . . Pagnoni, F. (2008). Feasibility and acceptability of artemisinin-based combination therapy for the home management of malaria in four African sites. Malar J, 7, 6. doi:10.1186/1475-2875-7-6
S105. Nanyonjo, A., Makumbi, F., Etou, P., Tomson, G., Källander, K., and inSCALE Study Group. Perceived quality of care for common childhood illnesses: facility versus community based providers in Uganda. PLoS ONE 8(11): e79943. doi:10.1371/journal.pone.0079943
S106. Moir, J. S., Tulloch, J. L., Vrbova, H., Jolley, D. J., Heywood, P. F., & Alpers, M. P. (1985). The role of voluntary village aides in the control of malaria by presumptive treatment of fever. 1. Selection, training and practice. P N G Med J, 28(4), 257-266.
S107. Linn, A.M., Ndiaye, Y., Hennessee, I., Gaye, S., Linn, P., Nordstrom, K., and Mc Laughkin, M. (2015). Reduction in symptomatic malaria prevalence through proactive community treatment in rural Senegal. Tropical Medicine and International Health, volume 20 no 11 pp 1438 – 1446
S108. Seikmans, K., Sohani, S., Kisia, J., Kiilu, K., Wamalwa, E., Nelima, F., Otieno, D.O., Nyandigisi, A., Akhwale, W., Ngindu, A. (2013). Community case management of malaria: a pro-poor intervention in rural Kenya. International health 5.3 (2013): 196-204.
S109. Care/Kenya. (1999). Community initiatives for child survival Siaya: Final evaluation
S110. Patouillard, E., Conteh, L., Webster, J., Kweku, M., Chandramohan, D., Greenwood, B. (2011). Coverage, Adherence and Costs of Intermittent Preventive Treatment of Malaria in Children Employing Different Delivery Strategies in Jasikan, Ghana. PlosONE. 2011(6)11: e24871
S111. Matovu, F., Nanyiti, A., Rutebemberwa, E. (2014). Household health care-seeking costs: experiences from a randomized, controlled trial of community-based malaria and

pneumonia treatment among under-fives in eastern Uganda. Malaria Journal 2014 13:222
S112. Mermin, J., Were, W., Ekwaru, J. P., Moore, D., Downing, R., Behumbiize, P., . . . Bunnell, R. (2008). Mortality in HIV-infected Ugandan adults receiving antiretroviral treatment and survival of their HIV-uninfected children: a prospective cohort study. Lancet, 371(9614), 752-759. doi:10.1016/S0140-6736(08)60345-1
S113. Lugada, E., Levin, J., Abang, B., Mermin, J., Mugalanzi, E., Namara, G., Gupta, S., Grosskurth, H., Jaffar, S., Coutinho, A., and Bunnell, R. (2010). Comparison of Home and Clinic-Based HIV Testing Among Household Members of Persons Taking Antiretroviral Therapy in Uganda: Results From a Randomized Trial. J Acquir Immune Defic Syndr, Volume 55, Number 2, October 1, 2010
S114. Gupta, N., Cyamatare, F.R., Niyigena, P., Niyigena, J.W., Stulac, S., Mugwaneza, P., Drobac, P., Rich, M., and Franke -Tittle, M.F. (2013). Clinical Outcomes of a Comprehensive Integrated Program for HIV-Exposed Infants: A 3-Year Experience Promoting HIV-Free Survival in Rural Rwanda. J Acquir Immune Defic Syndr _ Volume 62, Number 4
S115. Kim, M., Ahmed, S., Buck, W.C., Preidis, G.A., Hosseinipour, M.C., Bhalakia, A., Nanthuru, D., Kazembe, P.N., Chimbwandira, F., Giordano, T.P., Chiao, E.Y., Shutze, G.E., Kline, M.W. (2012). The Tingathe programme: a pilot intervention using community health workers to create a continuum of care in the prevention of mother to child transmission of HIV (PMTCT) cascade of services in Malawi. Journal of the International AIDS Society. 15(Suppl 2):17389, 1-11. Retrieved from: http://www.jiasociety.org/index.php/jias/article/view/17389 ¦ http://dx.doi.org/10.7448/IAS.15.4.17389
S116. Ahmed, S., Kim, M.H., Dave, A.C., Sabelli, R., Kanjelo, K., Preidis, G.A., Giordano, T.P., Chiao, E., Hosseinipour, M., Kazembel, P.N., Chimbwandira, F., Abrams, E.J. (2015). Improved identification and enrollment into care of HIV-exposed and -infected infants and children following a community health worker intervention in Lilongwe, Malawi. Journal of the International AIDS Society, 2015; http://dx.doi.org/10.7448/IAS.18.1.19305
S117. Kagaayi, J., Dreyfuss, M. L., Kigozi, G., Chen, M. Z., Wabwire-Mangen, F., Serwadda, D., . . . Gray, R. H. (2005). Maternal self-medication and provision of nevirapine to newborns by women in Rakai, Uganda. J Acquir Immune Defic Syndr, 39(1), 121-124. doi:http://dx.doi.org/10.1097/01.qai.0000148530.66587.7c
S118. Fatti, G., Shaikh, N., Eley, B., & Grimwood, A. (2014). Improved virological suppression in children on antiretroviral treatment receiving community-based adherence support: a multicentre cohort study from South Africa. AIDS Care, 26(4), 448-453. doi:10.1080/09540121.2013.855699
S119. Brugha, R. F., & Kevany, J. P. (1996). Maximizing immunization coverage through home visits: a controlled trial in an urban area of Ghana. Bull World Health Organ, 74(5), 517-524.
S120. Uzondu, C. A., Doctor, H. V., Findley, S. E., Afenyadu, G. Y., & Ager, A. (2015). Female health workers at the doorstep: a pilot of community-based maternal, newborn, and child health service delivery in northern Nigeria. Glob Health Sci Pract, 3(1), 97-108. doi:10.9745/GHSP-D-14-00117
S121. Tumwine, J. K., & Mackenzie, S. (1992). Child survival in a rural area in Zimbabwe: are we winning? Cent Afr J Med, 38(1), 30-36.
S122. Perry, H., Robison, N., Chavez, D., Taja, O., Hilari, C., Shanklin, D., & Wyon, J. (1998). The census-based, impact-oriented approach: its effectiveness in promoting child health in Bolivia. Health Policy Plan, 13(2), 140-151. doi:http://dx.doi.org/10.1093/heapol/13.2.140

S123. Perry, H. B., Shanklin, D. S., & Schroeder, D. G. (2003). Impact of a community-based comprehensive primary healthcare programme on infant and child mortality in Bolivia. J Health Popul Nutr, 21(4), 383-395.
S124. World Vision/Senegal & Thiam, L., (1994, Oct 2-7, 1994). Impact of the Thies CSP on the Health Knowledge and Practices of Mothers Living in the Sub-district of Niakhene (Thies region). Paper presented at the Community Impact of PVO Child Survival Efforts: 1995 - 1994, Bangalore, Karnataka, India.
S125. Matomora, M. K. (1989). A people-centered approach to primary health care implementation in Mvumi, Tanzania. Soc Sci Med, 28(10), 1031-1037. doi:http://dx.doi.org/10.1016/0277-9536(89)90385-7
S126. Perez-Cuevas, R., Reyes, H., Pego, U., Tome, P., Ceja, K., Flores, S., & Gutierrez, G. (1999). Immunization promotion activities: are they effective in encouraging mothers to immunize their children? Soc Sci Med, 49(7), 921-932. doi:http://dx.doi.org/10.1016/s0277-9536(99)00178-1
S127. Keoprasith, Bounserth, Kizuki M, Watanabe M, Takano T. (2013). The impact of community-based, workshop activities in multiple local dialects on the vaccination coverage, sanitary living and the health status of multiethnic populations in Lao PDR. Health promotion international 28.3 (2013): 453-465

S128. Dohn, A. L., Chavez, A., Dohn, M. N., Saturria, L., & Pimentel, C. (2004). Changes in health indicators related to health promotion and microcredit programs in the Dominican Republic. Rev Panam Salud Publica, 15(3), 185-193. doi:http://dx.doi.org/10.1590/s1020-49892004000300007
S129. Bilous, J., Maher, C., Tangermann, R. H., Aylward, R. B., Schnur, A., Sanders, R., . . . Omi, S. (1997). The experience of countries in the Western Pacific Region in conducting national immunization days for poliomyelitis eradication. J Infect Dis, 175 Suppl 1, S194-197. doi:http://dx.doi.org/10.1093/infdis/175.supplement_1.s194
S130. Foster, S. O., Spiegel, R. A., Mokdad, A., Yeanon, S., Becker, S. R., Thornton, J. N., & Galakpai, M. K. (1993). Immunization, oral rehydration therapy and malaria chemotherapy among children under 5 in Bomi and Grand Cape Mount counties, Liberia, 1984 and 1988. Int J Epidemiol, 22 Suppl 1, S50-55. doi:http://dx.doi.org/10.1093/ije/22.supplement_1.s50
S131. Perry, H., Cayemittes, M., Philippe, F., Dowell, D., Dortonne, J. R., Menager, H., . . . Berggren, G. (2006). Reducing under-five mortality through Hopital Albert Schweitzer's integrated system in Haiti. Health Policy Plan, 21(3), 217-230. doi:10.1093/heapol/czl005
S132. Linkins, R. W., Mansour, E., Wassif, O., Hassan, M. H., & Patriarca, P. A. (1995). Evaluation of house-to-house versus fixed-site oral poliovirus vaccine delivery strategies in a mass immunization campaign in Egypt. Bull World Health Organ, 73(5), 589-595
S133. Ciliberto, M. A., Sandige, H., Ndekha, M. J., Ashorn, P., Briend, A., Ciliberto, H. M., & Manary, M. J. (2005). Comparison of home-based therapy with ready-to-use therapeutic food with standard therapy in the treatment of malnourished Malawian children: a controlled, clinical effectiveness trial. Am J Clin Nutr, 81(4), 864-870.
S134. Bhandari, N., Bahl, R., Nayyar, B., Khokhar, P., Rohde, J. E., & Bhan, M. K. (2001). Food supplementation with encouragement to feed it to infants from 4 to 12 months of age has a small impact on weight gain. J Nutr, 131(7), 1946-1951.
S135. Mackintosh, U. A. T., Marsh, D. R., & Schroder, D. G. (2002). Sustained positive deviant child care practices and their effects on child growth in Viet Nam. Food and Nutrition Bulletin, 23(4), 16-24. doi:http://dx.doi.org/10.1177/15648265020234s204

S136. Awasthi, S., Pande, V. K., & Fletcher, R. H. (2000). Effectiveness and cost-effectiveness of albendazole in improving nutritional status of pre-school children in urban slums. Indian Pediatr, 37(1), 19-29.
S137. Tomlinson, M., Rotheram-Borus, M.J., Harwood, J., le Roux, I.M., O'Connor, M., Worthman, C. (2015). Community health workers can improve child growth of antenatally-depressed, South African mothers: a cluster randomized controlled trial. BMC psychiatry 15, no. 1 (2015): 1
S138. le Roux, I. M., Rotheram-Borus, M. J., Stein, J., & Tomlinson, M. (2014). The impact of paraprofessional home visitors on infants' growth and health at 18 months. Vulnerable Child Youth Stud, 9(4), 291-304. doi:10.1080/17450128.2014.940413
S139. Future Generations/Peru (2007). Internship Center Pilot Project The CLAS Las Moras-Huanuco: Mid-term evaluation.
S140. Christian, P., Khatry, S. K., & West, K. P., Jr. (2004). Antenatal anthelmintic treatment, birthweight, and infant survival in rural Nepal. Lancet, 364(9438), 981-983. doi:10.1016/S0140-6736(04)17023-2
S141. SAVE/HAITI (1994). Impact of sustainable behavior change on the nutritional status of children. Paper presented at the Community Impact of PVO Child Survival Efforts: 1985-1994, Bangalore, Karnataka, India.
S142. MkNelly, B., & Dunford, C. (1998). Impact of credit with education on mother and their young children's nutrition: Lower Pra rural bank credit with education program in Ghana. Freedom from hunger Research Paper no. 4
S143. Schroeder, D. G., Pachon, H., Dearden, K. A., Ha, T. T., Lang, T. T., & Marsh, D. R. (2002). An integrated child nutrition intervention improved growth of younger more malnourished children in northern Viet Nam. Food and Nutrition Bulletin, vol 23 supplement 50-58
S144. SAVE/Bangladesh, & Kaye, K., Khan, N. H., Hossain, A. (1994, Oct 2-7, 1994). Effect of a nutrition education program on the weight of younger siblings of malnourished children in Bangladesh. Paper presented at the Community Impact of PVO Child Survival Efforts: 1985-1994, Bangalore, Karnataka, India.
S145. Helen Keller International in collaboration with the Nepali Technical Assistance Group. (2013). Action Against Malnutrition Through Agriculture: Nepal Child Survival Project. Final Evaluation Report. October 1, 2008-December 31, 2012. Judiann McNulty, External consultant
S146. Miller, L., Josh,i N., Lohani, M., Rogers, B., Loraditch, M., Houser, R., Singh, P., Mahato, S. (2014). Community development and livestock promotion in rural Nepal: Effects on child growth and health. Food and Nutrition Bulletin, Vol. 35 No. 3
S147. Fernald, L.C.H., Gertler, P.J., Neufeld, L.M. . 10-year effect of Oportunidades, Mexico's conditional cash transfer programme, on child growth, cognition, language, and behaviour: a longitudinal follow-up study Lancet 2009:374:1997- 2005
S148. Morris, S. S., Flores, R., Olinto, P., & Medina, J. M. (2004). Monetary incentives in primary health care and effects on use and coverage of preventive health care interventions in rural Honduras: cluster randomised trial. Lancet, 364(9450), 2030-2037. doi:10.1016/S0140-6736(04)17515-6
S149. Paxson, C., & Schady, N. (2007). Does money matter?: The effects of cash transfers on child health and development in rural Ecuador. Retrieved from World Bank Policy Research Working Paper 4426:
S150. Fernald, L. C., Gertler, P. J., & Neufeld, L. M. (2008). Role of cash in conditional cash transfer programmes for child health, growth, and development: an analysis of Mexico's Oportunidades. Lancet, 371(9615), 828-837. doi:10.1016/S0140-6736(08)60382-7
S151. Bhandari, N., Bahl, R., Mazumdar, S., Martines, J., Black, R. E., Bhan, M. K., & Infant Feeding Study, G. (2003). Effect of community-based promotion of exclusive

breastfeeding on diarrhoeal illness and growth: a cluster randomised controlled trial. Lancet, 361(9367), 1418-1423. doi:10.1016/S0140-6736(03)13134-0

S152. Kiman-Murage, E., Norris, S., Mutua, M., Wekesah, F., Wanjoh,i M., Muhia, N., Muriuki, P., Egondi, T., Kyobutungi, C., Ezeh, A., Musoke, R., McGarvey, S., Madise, N., Griffiths. (2015). Potential effectiveness of Community Health Strategy to promote exclusive breastfeeding in urban poor settings in Nairobi, Kenya: a quasi-experimental study. Journal of developmental origins of health and disease, p.1-13.

S153. Coutinho, S. B., de Lira, P. I., de Carvalho Lima, M., & Ashworth, A. (2005). Comparison of the effect of two systems for the promotion of exclusive breastfeeding. Lancet, 366(9491), 1094-1100. doi:10.1016/S0140-6736(05)67421-1

S154. Haider, R., Ashworth, A., Kabir, I., & Huttly, S. R. (2000). Effect of community-based peer counsellors on exclusive breastfeeding practices in Dhaka, Bangladesh: a randomised controlled trial [see commments]. Lancet, 356(9242), 1643-1647. doi:http://dx.doi.org/10.1016/s0140-6736(00)03159-7

S155. Saleem, A. F., Mahmud, S., Baig-Ansari, N., & Zaidi, A. K. (2014). Impact of maternal education about complementary feeding on their infants' nutritional outcomes in low- and middle-income households: a community-based randomized interventional study in Karachi, Pakistan. J Health Popul Nutr, 32(4), 623-633.

S156. Guyon, A., Rambeloson, Z., Hainsworth, M., & Quinn, V. (2004). Assesing a behavior change strategy for The Essential Nutrition Actions, Immunization and Family Planning: Antananarivo and Fianarantsoa Provinces, Madagascar.

S157. Lutter, C. K., Rodriguez, A., Fuenmayor, G., Avila, L., Sempertegui, F., & Escobar, J. (2008). Growth and micronutrient status in children receiving a fortified complementary food. J Nutr, 138(2), 379-388.

S158. Care/Sierra Leone (2008). Child survival project ‘For Di Pikin Dem Wel Bodi’, Koinadugu District, Northern Region, Sierra Leone: Final evaluation.

S159. Sternin, M., Sternin, J., & Marsh, D. L. (1999). Scaling up a poverty alleviation and nutrition program in Vietnam. In T. Marchione (Ed.), Scaling up, scaling down: Overcoming malnutrition in developing countries (pp. 97-117). Amsterdam, The Netherlands: Gordon and Breach.

S160. Berggren, G. (1997). Nutritional education and rehabilitation program: A Save the Children project. In O. Wollinka et al (Eds.), Vietnam Hearth Nutrition Model: Applications in Haiti, Vietnam, and Bangladesh (pp. 43-46). Arlington, VA: World Relief

S161. Rahmathullah, L., Underwood, B. A., Thulasiraj, R. D., Milton, R. C., Ramaswamy, K., Rahmathullah, R., & Babu, G. (1990). Reduced mortality among children in southern India receiving a small weekly dose of vitamin A. N Engl J Med, 323(14), 929-935. doi:10.1056/NEJM199010043231401

S162. Christian, P., Khatry, S. K., Katz, J., Pradhan, E. K., LeClerq, S. C., Shrestha, S. R., . . . West, K. P., Jr. (2003). Effects of alternative maternal micronutrient supplements on low birth weight in rural Nepal: double blind randomised community trial. BMJ, 326(7389), 571. doi:10.1136/bmj.326.7389.571

S163. West, K. P., Jr., Pokhrel, R. P., Katz, J., LeClerq, S. C., Khatry, S. K., Shrestha, S. R., . . . Sommer, A. (1991). Efficacy of vitamin A in reducing preschool child mortality in Nepal. Lancet, 338(8759), 67-71. doi:http://dx.doi.org/10.1016/0140-6736(91)90070-6

S164. Herrera, M. G., Nestel, P., el Amin, A., Fawzi, W. W., Mohamed, K. A., & Weld, L. (1992). Vitamin A supplementation and child survival. Lancet, 340(8814), 267-271. doi:http://dx.doi.org/10.1016/0140-6736(92)92357-l

S165. Ghana VAST Study Team (1993). Vitamin A supplementation in northern Ghana: effects on clinic attendances, hospital admissions, and child mortality. Lancet, 342(8862), 7-12. doi:http://dx.doi.org/10.1016/0140-6736(93)91879-q

S166. Bishai, D., Kumar, K. C. S., Waters, H., Koenig, M., Katz, J., Khatry, S. K., & West, K. P., Jr. (2005). The impact of vitamin A supplementation on mortality inequalities among children in Nepal. Health Policy Plan, 20(1), 60-66. doi:10.1093/heapol/czi007
S167. Sommer, A., Tarwotjo, I., Djunaedi, E., West, K. P., Jr., Loeden, A. A., Tilden, R., & Mele, L. (1986). Impact of vitamin A supplementation on childhood mortality. A randomised controlled community trial. Lancet, 1(8491), 1169-1173.
S168. Rahmathullah, L., Tielsch, J. M., Thulasiraj, R. D., Katz, J., Coles, C., Devi, S., . . . Kamaraj, C. (2003). Impact of supplementing newborn infants with vitamin A on early infant mortality: community based randomised trial in southern India. BMJ, 327(7409), 254. doi:10.1136/bmj.327.7409.254
S169. Klemm, R. D., Labrique, A. B., Christian, P., Rashid, M., Shamim, A. A., Katz, J., . . . West, K. P., Jr. (2008). Newborn vitamin A supplementation reduced infant mortality in rural Bangladesh. Pediatrics, 122(1), e242-250. doi:10.1542/peds.2007-3448
S170. Dibley, M. J., Sadjimin, T., Kjolhede, C. L., & Moulton, L. H. (1996). Vitamin A supplementation fails to reduce incidence of acute respiratory illness and diarrhea in preschool-age Indonesian children. J Nutr, 126(2), 434-442.
S171. UNICEF/Rajasthan. (2007). Anchal se angan tak: Community based integrated nutrition strategy, Rajasthan.
S172. Zeba, A. N., Sorgho, H., Rouamba, N., Zongo, I., Rouamba, J., Guiguemde, R. T., . . . Ouedraogo, J. B. (2008). Major reduction of malaria morbidity with combined vitamin A and zinc supplementation in young children in Burkina Faso: a randomized double blind trial. Nutr J, 7, 7. doi:10.1186/1475-2891-7-7
S173. Sazawal, S., Black, R. E., Ramsan, M., Chwaya, H. M., Dutta, A., Dhingra, U., . . . Kabole, F. M. (2007). Effect of zinc supplementation on mortality in children aged 1-48 months: a community-based randomised placebo-controlled trial. Lancet, 369(9565), 927-934. doi:10.1016/S0140-6736(07)60452-8
S174. Gupta, D. N., Rajendran, K., Mondal, S. K., Ghosh, S., & Bhattacharya, S. K. (2007). Operational feasibility of implementing community-based zinc supplementation: impact on childhood diarrheal morbidity. Pediatr Infect Dis J, 26(4), 306-310. doi:10.1097/01.inf.0000258692.65485.d9
S175. Penny, M. E., Peerson, J. M., Marin, R. M., Duran, A., Lanata, C. F., Lonnerdal, B., . . . Brown, K. H. (1999). Randomized, community-based trial of the effect of zinc supplementation, with and without other micronutrients, on the duration of persistent childhood diarrhea in Lima, Peru. J Pediatr, 135(2 Pt 1), 208-217. doi:http://dx.doi.org/10.1016/s0022-3476(99)70024-7
S176. Sazawal, S., Black, R. E., Ramsan, M., Chwaya, H. M., Stoltzfus, R. J., Dutta, A., . . . Kabole, F. M. (2006). Effects of routine prophylactic supplementation with iron and folic acid on admission to hospital and mortality in preschool children in a high malaria transmission setting: community-based, randomised, placebo-controlled trial. Lancet, 367(9505), 133-143. doi:10.1016/S0140-6736(06)67962-2
S177. Suchdev, P. S., Ruth, L. J., Woodruff, B. A., Mbakaya, C., Mandava, U., Flores-Ayala, R., . . . Quick, R. (2012). Selling Sprinkles micronutrient powder reduces anemia, iron deficiency, and vitamin A deficiency in young children in Western Kenya: a cluster-randomized controlled trial. Am J Clin Nutr, 95(5), 1223-1230. doi:10.3945/ajcn.111.030072
S178. Lundeen, E. (Kyrgyz-Swiss-Swedish Health Project, Bishkek, Kyrgyz Republic); Schueth, T. (Kyrgyz-Swiss-Swedish Health Project, Bishkek, Kyrgyz Republic); Toktobaev, N. (Kyrgyz-Swiss-Swedish Health Project, Bishkek, Kyrgyz Republic); Zlotkin, S. (Hospital for Sick Children, Toronto); Hyder, S.M.Z. (World Bank and Dalla Lana School of Public Halth, Toronto); Houser, R. (Tufts university Friedman School of Nutrition Science and Policy, Boston). Daily use of Sprinkles micronutrient powder for 2 months reduces anemia

among children 6 to 36 months of age in the Kyrgyz Republic: A cluster-randomized trial. Food and Nutrition Bulletin, 2010(31)3:446-460

S179. Kounnavong, S. (National Institute of Public Health, Vientiane, Lao People's Democratic Republic and Department of International Health, Institute of Tropical Medicine and Global Centre of Excellence Program, Nagasaki, Japan); Sunahara, T. (Department of International Health, Institute of Tropical Medicine and Global Centre of Excellence Program, Nagasaki, Japan); Mascie-Taylor, C.G.N. (Division of Biological Anthropology, Department of Archaeology and Anthropology, University of Cambridge, UK); Hashizume, M. (Department of International Health, Institute of Tropical Medicine and Global Centre of Excellence Program, Nagasaki, Japan); Okumura, J. (Department of International Health, Institute of Tropical Medicine and Global Centre of Excellence Program, Nagasaki, Japan); Moji, K. (Research Institute for Humanity and Nature, Kyoto, Japan); Boupha, B. (National Institute of Public Health, Vientiane, Lao People's Democratic Republic); Yamamoto, T. (Department of International Health, Institute of Tropical Medicine and Global Centre of Excellence Program, Nagasaki, Japan). Effect of daily versus weekly home fortification with multiple micronutrient powder on haemoglobin concentration of young children in a rural area, Lao People's Democratic Republic: a randomised trial. Nutrition Journal 2011, 10:129.
S180. Muhilal, Permeisih, D., Idjradinata, Y. R., Muherdiyantiningsih, & Karyadi, D. (1988). Vitamin A-fortified monosodium glutamate and health, growth, and survival of children: a controlled field trial. Am J Clin Nutr, 48(5), 1271-1276.
S181. Fauveau, V., Wojtyniak, B., Chakraborty, J., Sarder, A. M., & Briend, A. (1990). The effect of maternal and child health and family planning services on mortality: is prevention enough? BMJ, 301(6743), 103-107. doi:http://dx.doi.org/10.1136/bmj.301.6743.103
S182. Arifeen, S. E., Hoque, D. M., Akter, T., Rahman, M., Hoque, M. E., Begum, K., . . . Black, R. E. (2009). Effect of the Integrated Management of Childhood Illness strategy on childhood mortality and nutrition in a rural area in Bangladesh: a cluster randomised trial. Lancet, 374(9687), 393-403. doi:10.1016/S0140-6736(09)60828-X
S183. BASICS II/Madagascar (2004). Improving family health using an integrated community-based approach: Madagarcar Case Study.
S184. Matomora, M. K. (1989). A people-centered approach to primary health care implementation in Mvumi, Tanzania. Soc Sci Med, 28(10), 1031-1037. doi:http://dx.doi.org/10.1016/0277-9536(89)90385-7
S185. Mugeni, C., Levine, A. C., Munyaneza, R. M., Mulindahabi, E., Cockrell, H. C., Glavis-Bloom, J., . . . Binagwaho, A. (2014). Nationwide implementation of integrated community case management of childhood illness in Rwanda. Glob Health Sci Pract, 2(3), 328-341. doi:10.9745/GHSP-D-14-00080
S186. Ameha, A., Karim, A. M., Erbo, A., Ashenafi, A., Hailu, M., Hailu, B., . . . Betemariam, W. (2014). Effectiveness of supportive supervision on the consistency of integrated community cases management skills of the health extension workers in 113 districts of Ethiopia. Ethiop Med J, 52 Suppl 3, 65-71.
S187. Wogi, A., Teno, D., Bulto, T., Deressa, W., Alemu, H., & Nigussie, M. (2014). Effect of integrated community case management of common childhood illnesses on the quality of malaria case management provided by health extension workers at health posts. Ethiop Med J, 52 Suppl 3, 99-108.

S188. Awor, P., Wamani, H., Tylleskar, T., & Peterson, S. (2015). Drug seller adherence to clinical protocols with integrated management of malaria, pneumonia and diarrhoea at drug shops in Uganda. Malar J, 14, 277. doi:10.1186/s12936-015-0798-9

S189. Langston, A., Weiss, J., Landegger, J., Pullum, T., Morrow, M., Kabadege, M., . . . Sarriot, E. (2014). Plausible role for CHW peer support groups in increasing care-seeking in an integrated community case management project in Rwanda: a mixed methods evaluation. Glob Health Sci Pract, 2(3), 342-354. doi:10.9745/GHSP-D-14-00067

S190. Sarriot, E. (2011). Final Evaluation of the Kabeho Mwana Expanded Impact Child Survival Program. December 22, 2011.

S191. Plan/Cameroon. (2004). Child survival project: Final evaluation report.

S192. Mugeni, C., Levine, A. C., Munyaneza, R. M., Mulindahabi, E., Cockrell, H. C., Glavis-Bloom, J., . . . Binagwaho, A. (2014). Nationwide implementation of integrated community case management of childhood illness in Rwanda. Glob Health Sci Pract, 2(3), 328-341. doi:10.9745/GHSP-D-14-00080

S193. Davis, T. P., Jr., Wetzel, C., Hernandez Avilan, E., de Mendoza Lopes, C., Chase, R. P., Winch, P. J., & Perry, H. B. (2013). Reducing child global undernutrition at scale in Sofala Province, Mozambique, using Care Group Volunteers to communicate health messages to mothers. Glob Health Sci Pract, 1(1), 35-51. doi:10.9745/GHSP-D-12-00045

S194. Edward, A., Ernst, P., Taylor, C., Becker, S., Mazive, E., & Perry, H. (2007). Examining the evidence of under-five mortality reduction in a community-based programme in Gaza, Mozambique. Trans R Soc Trop Med Hyg, 101(8), 814-822. doi:10.1016/j.trstmh.2007.02.025

S195. World Relief/Burundi. (2012). ”Ramba Kibondo “Live Long Child” Child Survival Project Final Evaluation Report 2012.” Paulette Chaponniere, external consultant along with World Relief staff authored this report. Project dates: September 2007-2012. World Relief Burundi.

S196. Pence, B. W., Nyarko, P., Phillips, J. F., & Debpuur, C. (2007). The effect of community nurses and health volunteers on child mortality: the Navrongo Community Health and Family Planning Project. Scand J Public Health, 35(6), 599-608. doi:10.1080/14034940701349225

S197. Binka, F. N., Bawah, A. A., Phillips, J. F., Hodgson, A., Adjuik, M., & MacLeod, B. (2007). Rapid achievement of the child survival millennium development goal: evidence from the Navrongo experiment in Northern Ghana. Trop Med Int Health, 12(5), 578-583. doi:10.1111/j.1365-3156.2007.01826.x

S198. Phillips, J. F., Bawah, A. A., & Binka, F. N. (2006). Accelerating reproductive and child health programme impact with community-based services: the Navrongo experiment in Ghana. Bull World Health Organ, 84(12), 949-955. doi:http://dx.doi.org/10.2471/blt.06.030064

S199. Perry, H., Robison, N., Chavez, D., Taja, O., Hilari, C., Shanklin, D., & Wyon, J. (1998). The census-based, impact-oriented approach: its effectiveness in promoting child health in Bolivia. Health Policy Plan, 13(2), 140-151. doi:http://dx.doi.org/10.1093/heapol/13.2.140

S200. Perry, H. B., Shanklin, D. S., & Schroeder, D. G. (2003). Impact of a community-based comprehensive primary healthcare programme on infant and child mortality in Bolivia. J Health Popul Nutr, 21(4), 383-395.

S201. Perry, H., Cayemittes, M., Philippe, F., Dowell, D., Dortonne, J. R., Menager, H., . . . Berggren, G. (2006). Reducing under-five mortality through Hopital Albert Schweitzer's integrated system in Haiti. Health Policy Plan, 21(3), 217-230. doi:10.1093/heapol/czl005

S202. Berggren, W. L., Ewbank, D. C., & Berggren, G. G. (1981). Reduction of mortality in rural Haiti through a primary health-care program. N Engl J Med, 304(22), 1324-1330. doi:10.1056/NEJM198105283042203
S203. Berggren, G. G., Hebert, J. R., & Waternaux, C. M. (1985). Comparison of Haitian children in a nutrition intervention programme with children in the Haitian national nutrition survey. Bull World Health Organ, 63(6), 1141-1150.
S204. Kark, S. L., & Cassel, J. (1952). The Pholela Health Centre; a progress report. S Afr Med J, 26(7), 131-136; concl. doi:http://dx.doi.org/10.2105/ajph.92.11.1743
S205. Kielmann, A.A., Taylor, C.E., DeSweemer, C., et al. (1983). Volume 1. Child and Maternal Health Services in Rural India: the Narangwal Experiment. Integrated Nutrition and Health Care. Baltimore: Published for the World Bank [by] Johns Hopkins University Press
S206. Taylor, C.E., Sarma, R.S.S., Parker, R.L., Reinke, W.A., Faruqee, R. (1983). Volume 2. Child and Maternal Health Services in Rural India: the Narangwal Experiment. Integrated Family Planning and Health Care. Baltimore, MD: The Johns Hopkins University Press
S207. Kielmann, A.A., Taylor, C.E., DeSweemer, C., et al.(1983) Volume 1. Child and Maternal Health Services in Rural India: the Narangwal Experiment. Integrated Nutrition and Health Care. Baltimore: Published for the World Bank [by] Johns Hopkins University Press
